# Supplementary material for: Ketogenic diet but not free-sugar restriction alters glucose tolerance, lipid metabolism, peripheral tissue phenotype, and gut microbiome: RCT
Source: Cell Rep Med. 2024 Aug 5;5(8):101667. doi: 10.1016/j.xcrm.2024.101667 (PMC11384946; doi:10.1016/j.xcrm.2024.101667)
Supplement: Document S1. Figures S1–S6 and Tables S1–S7, S9, and S10 [file mmc1.pdf]

Cell Reports Medicine, Volume *5*

Supplemental information

Ketogenic diet but not free-sugar restriction alters glucose tolerance, lipid metabolism, peripheral tissue phenotype, and gut microbiome: RCT

Aaron Hengist, Russell G. Davies, Jean-Philippe Walhin, Jariya Buniam, Lucy H. Merrell, Lucy Rogers, Louise Bradshaw, Alfonso Moreno-Cabañas, Peter J. Rogers, Jeff M. Brunstrom, Leanne Hodson, Luc J.C. van Loon, Wiley Barton, Ciara O'Donovan, Fiona Crispie, Orla O'Sullivan, Paul D. Cotter, Kathryn Proctor, James A. Betts, Françoise Koumanov, Dylan Thompson, and Javier T. Gonzalez

SUPPLEMENTAL INFORMATION

Title: Ketogenic diet (but not free-sugar restriction) alters glucose tolerance, lipid metabolism, peripheral tissue phenotype and gut microbiome: RCT.

Author list: Aaron Hengist*[#a], Russell G Davies[#a], Jean-Philippe Walhin[a], Jariya Buniam[a,b], Lucy H Merrell[a], Lucy Rogers[a], Louise Bradshaw[a], Alfonso Moreno-Cabañas[a], Peter J Rogers[c], Jeff M Brunstrom[c], Leanne Hodson[d], Luc JC van Loon[e], Wiley Barton[f,g,h], Ciara O'Donovan[f,g], Fiona Crispie[f,g], Orla O'Sullivan[f,g,h], Paul D Cotter[f,g,h], Kathryn Proctor[a], James A Betts[a], Françoise Koumanov[a], Dylan Thompson[a], Javier T Gonzalez*[a]

This document contains 6 supplemental figures and 9 supplemental tables.

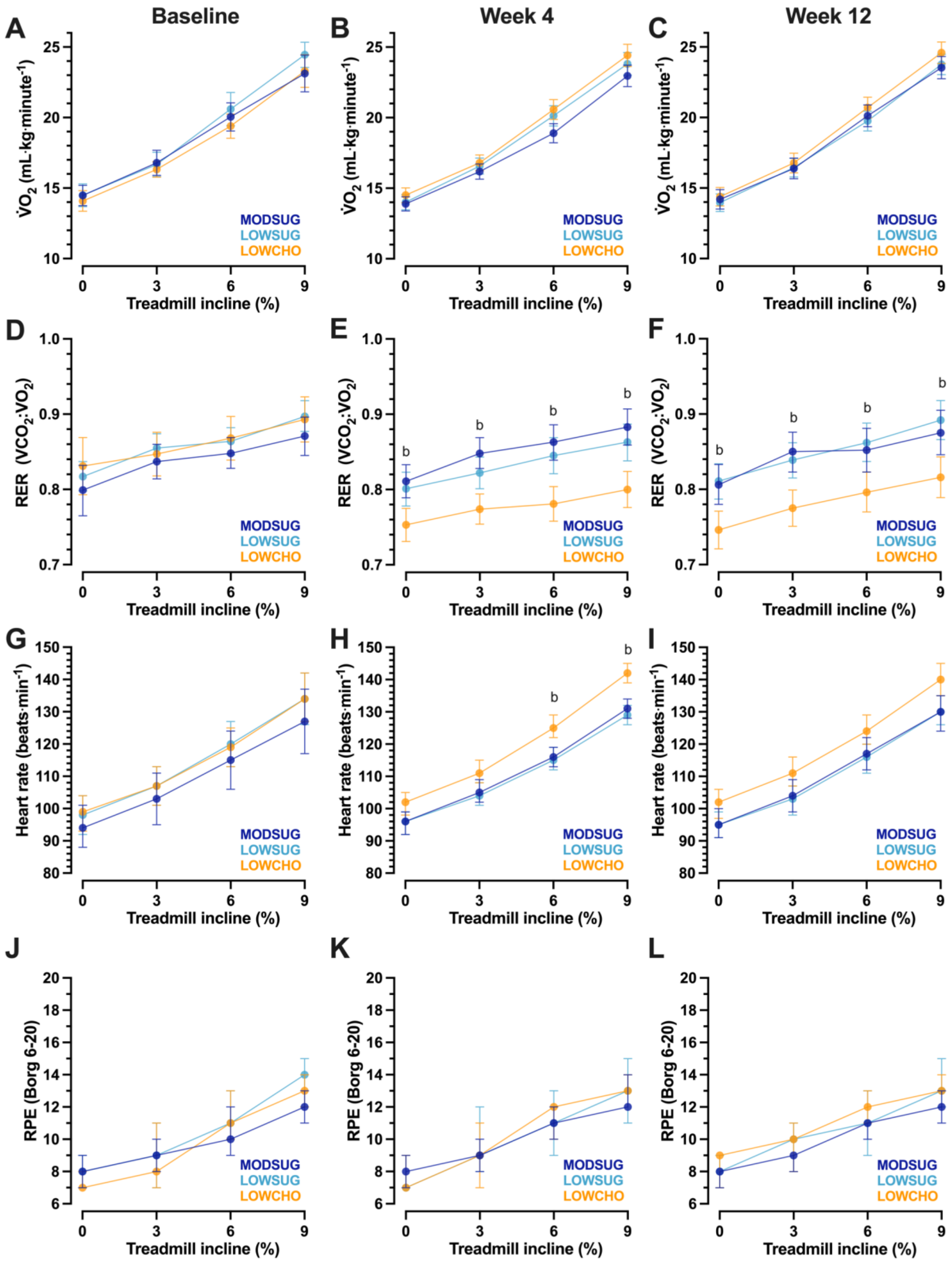

Figure S1. Physiological responses during incremental exercise tests at baseline and following 4 weeks and 12 weeks of moderate-sugar diet (MODSUG), low-sugar diet (LOWSUG), or low-carbohydrate diet (LOWCHO) in healthy men and women. Related to Figure 1. Relative $\dot{V}O_2$ (**A-C**), respiratory exchange ratio (RER) (**D-F**), heart rate (**G-I**), and rate of perceived exertion (RPE) (**J-L**) during exercise at 5.2 km·h^{-1} and incremental inclines from 0 to 9%. Data shown are mean (95% CI) for Baseline and ANCOVA-adjusted mean (95% CI) for Week 4 and Week 12. Week 4 n=52, Week 12 n=45. $^{b}p \leq 0.05$ MODSUG *vs* LOWCHO.

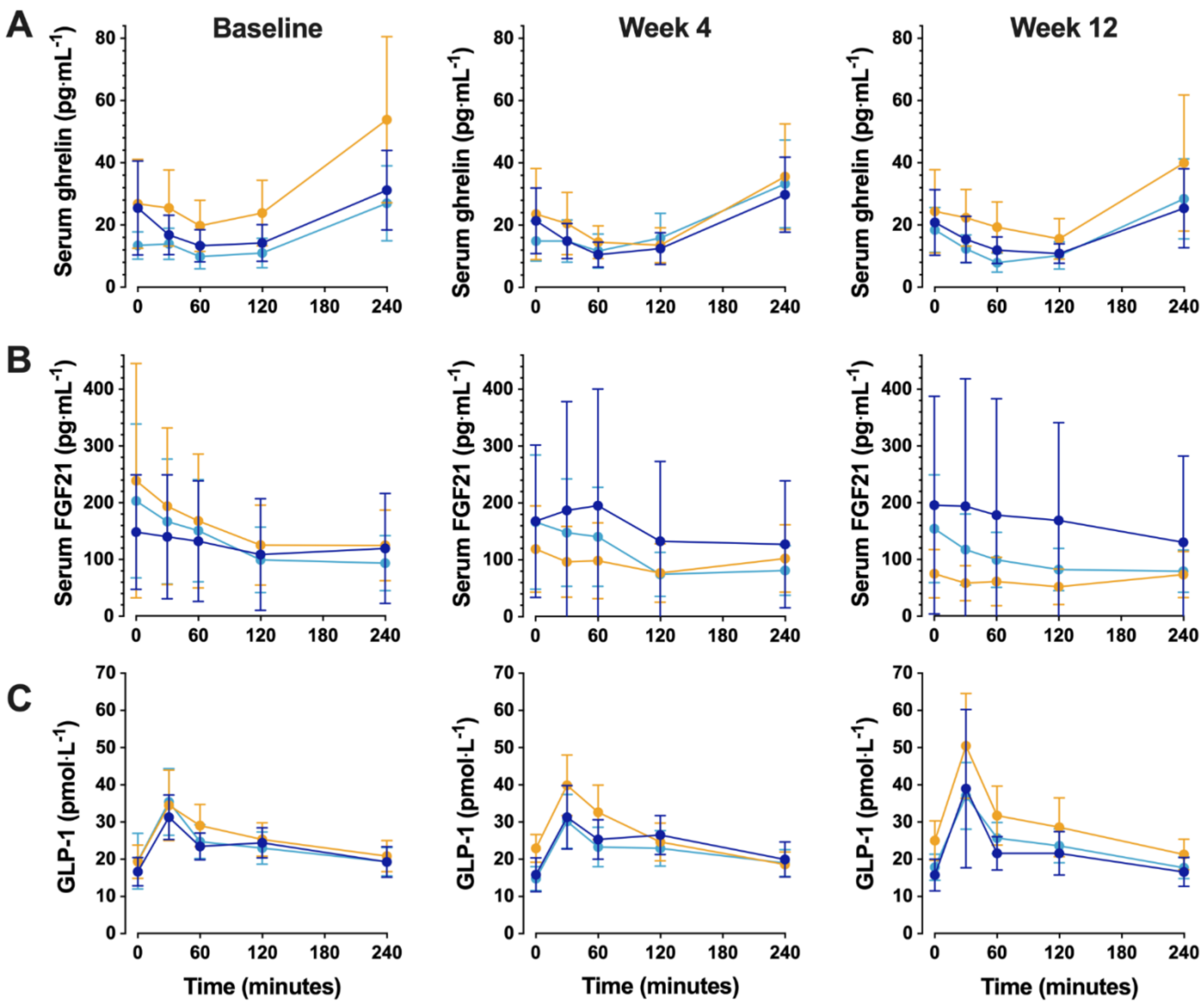

Figure S2. Serum ghrelin, fibroblast growth factor 21 (FGF21), and glucagon-like peptide-1 (GLP-1) responses to mixed meal tolerance tests (mean [range] 502 [331 to 715 kcal]; 54% carbohydrate [23% of which sugars], 31% fat, 15% protein) at baseline, week 4, and week 12 of moderate sugar (MODSUG), free-sugar restriction (LOWSUG), or ketogenic carbohydrate restricted (LOWCHO) diets. Related to Figure 7. Unadjusted postprandial concentrations of serum ghrelin (**A**), FGF21 (**B**) GLP-1 (**C**). Week 4 n=43-50; week 12 n=38-42.

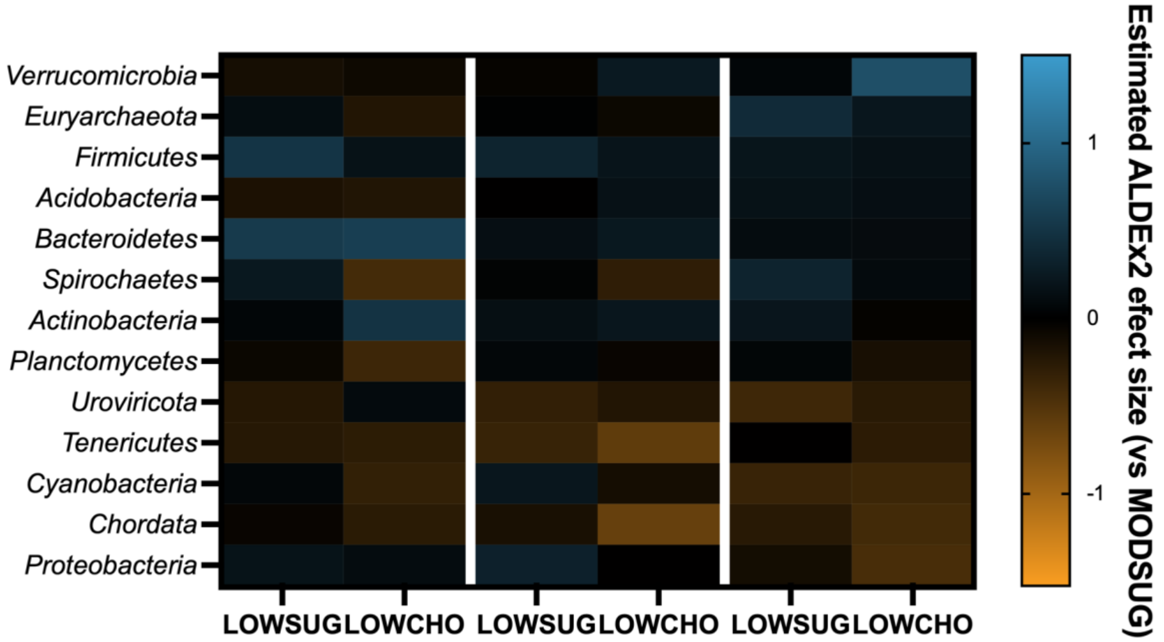

Figure S3. Differences in relative abundance of the gut microbiome at the phylum level at baseline, week 4, and week 12 free-sugar restriction (LOWSUG), or ketogenic carbohydrate restricted (LOWCHO) diets compared with moderate sugar (MODSUG) Related to Figure 5. Estimated effect size of change in center log ratio abundance of gut microbiome phyla vs MODSUG. Week 4 *n*=48, week 12 *n*=41.

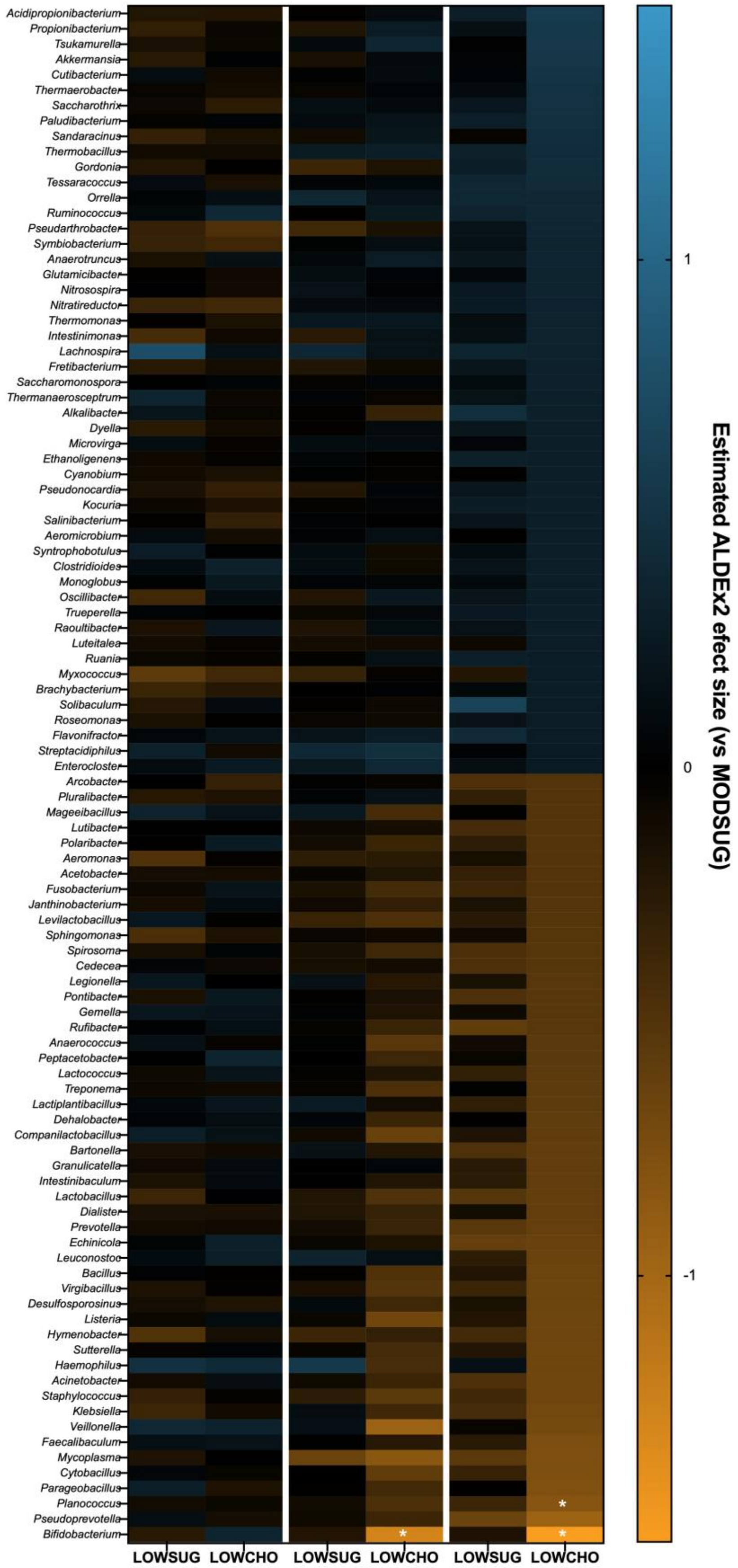

Figure S4. Differences in relative abundance of the gut microbiome at the genus level at baseline (left-hand), week 4 (center), and week 12 (right-hand) with free-sugar restriction (LOWSUG), or ketogenic carbohydrate restricted (LOWCHO) diets compared with moderate sugar (MODSUG). Related to Figure 5. Fifty largest increases and 50 largest decreases in estimated effect size of change in center log ratio abundance vs MODSUG are presented. Week 4 *n*=48, week 12 *n*=41.

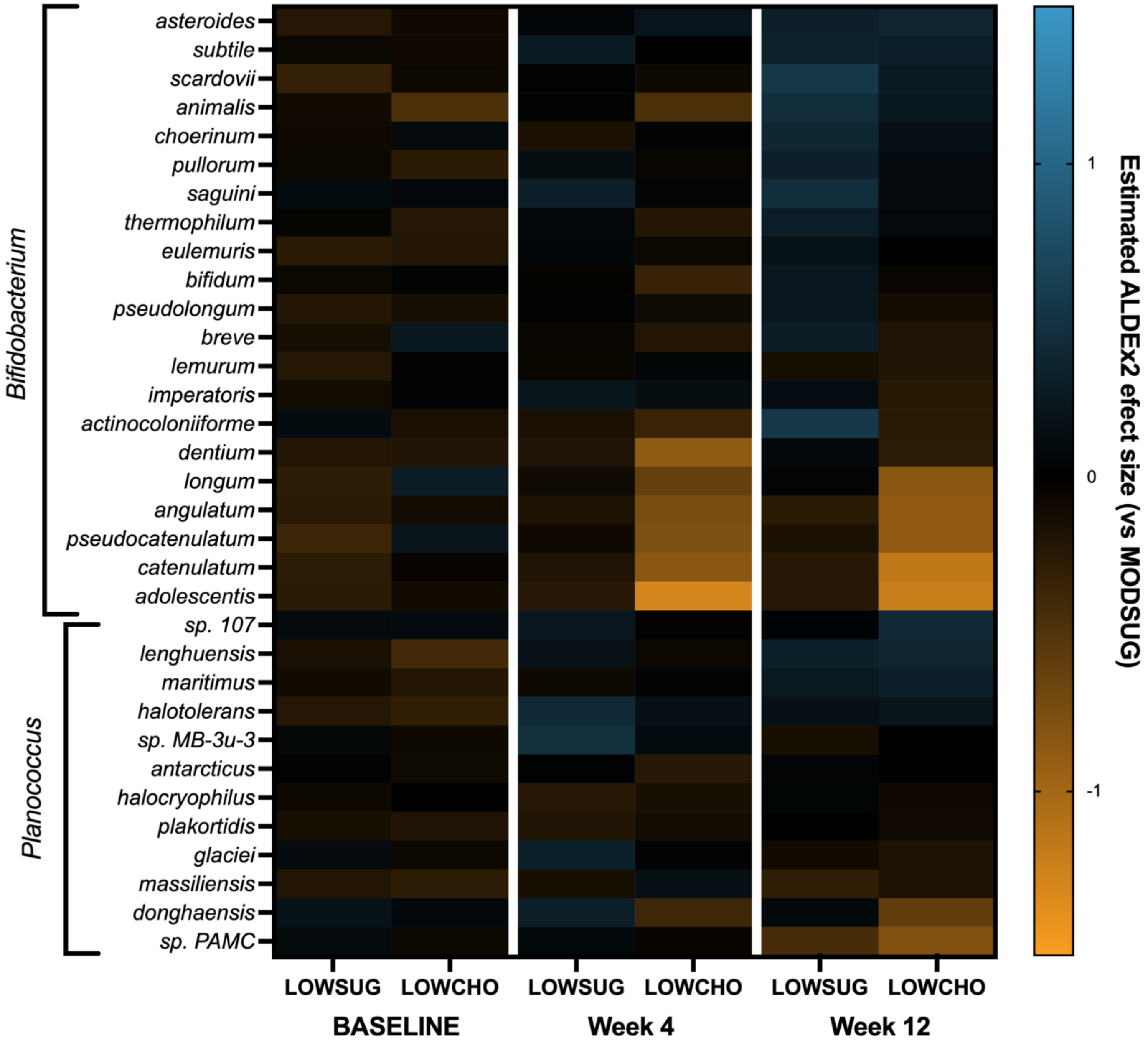

Figure S5. Differences in relative abundance of the gut microbiome at the species level for *Bifidobacterium* and *Planoccocus* at baseline (left-hand), week 4 (center), and week 12 (right-hand) with free-sugar restriction (LOWSUG), or ketogenic carbohydrate restricted (LOWCHO) diets compared with moderate sugar (MODSUG). Related to Figure 5. Estimated effect size of change in center log ratio abundance of gut microbiome species vs MODSUG. Week 4 *n*=48, week 12 *n*=41.

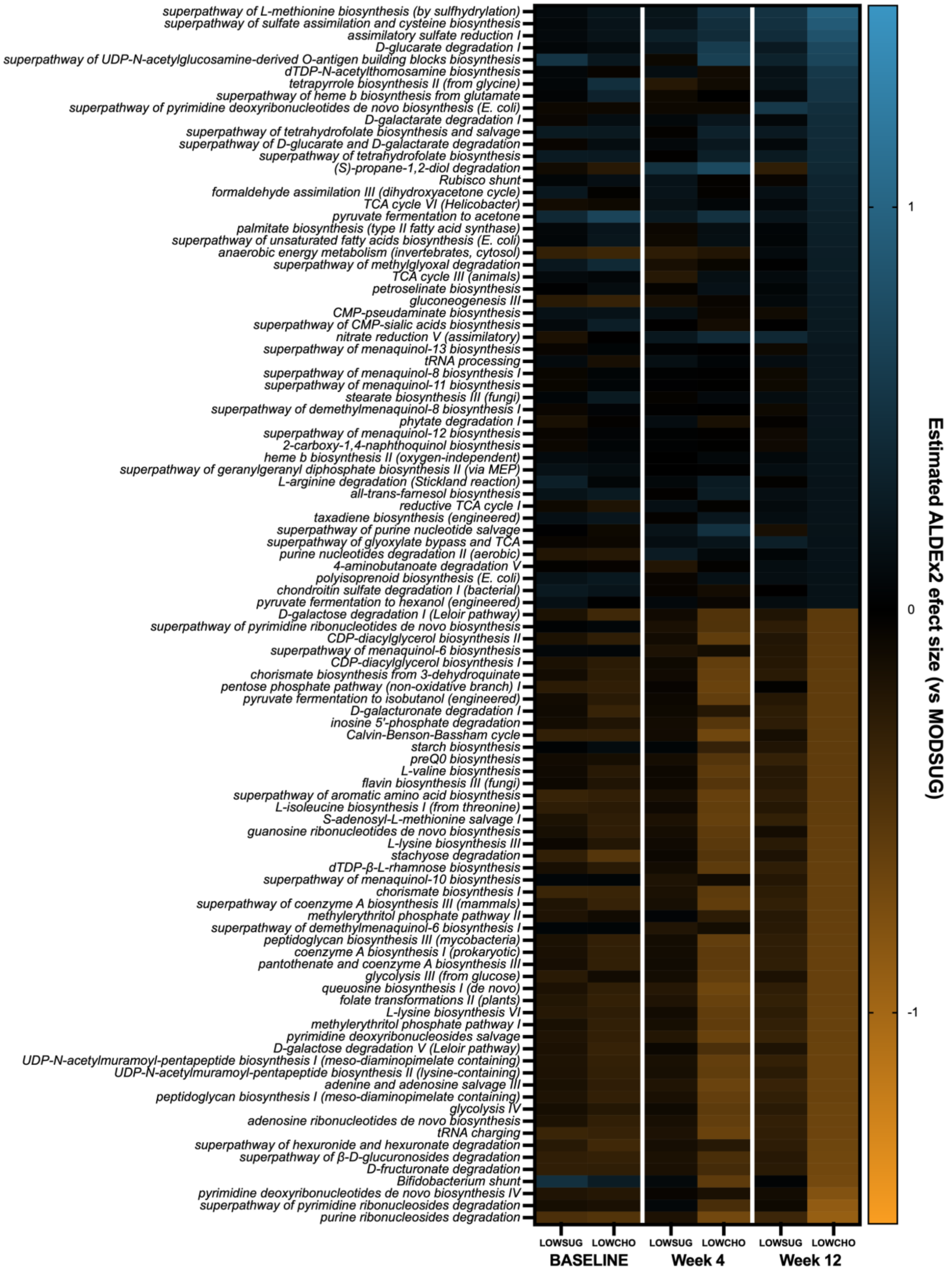

Figure S6. Differences in HUMANn2 metabolic pathways abundance at baseline (left-hand), week 4 (center), and week 12 (right-hand) with free-sugar restriction (LOWSUG), or ketogenic carbohydrate restricted (LOWCHO) diets compared with moderate sugar (MODSUG). Related to Figure 5. Fifty largest increases and 50 largest decreases in estimated effect size of change in center log ratio abundance vs MODSUG are presented. Week 4 *n*=48, week 12 *n*=41.

Table S1. Baseline characteristics for participants following a moderate-sugar control (MODSUG), low-sugar (LOWSUG), or low-carbohydrate diets (LOWCHO). Related to Figure 1.

	Week 4 completers			Week 12 completers		
	**MODSUG**	**LOWSUG**	**LOWCHO**	**MODSUG**	**LOWSUG**	**LOWCHO**
n (female, male)	18 (11, 7)	17 (10, 7)	18 (9, 9)	13 (8, 5)	16 (10, 6)	16 (7, 9)
Age (years)	39 ± 17	31 ± 16	33 ± 14	41 ± 16	32 ± 16	34 ± 14
Height (m)	1.72 ± 0.1	1.71 ± 0.05	1.71 ± 0.1	1.72 ± 0.11	1.71 ± 0.06	1.72 ± 0.1
Body mass (kg)	72.1 ± 10.8	69.4 ± 7	73.4 ± 13.4	73.3 ± 11.3	69.4 ± 7.2	75.5 ± 12.8
BMI ($kg\cdot m^{-2}$)	24.1 ± 2.7	23.5 ± 2	24.8 ± 3	24.6 ± 2.8	23.6 ± 2.1	25.2 ± 2.9
Waist circumference (cm)	82 ± 9.2	79.3 ± 6.1	83.9 ± 9.3	83.3 ± 10.3	79 ± 6.1	85.8 ± 8
Hip circumference (cm)	101.2 ± 5.4	99.4 ± 5.5	101.5 ± 6.9	101.8 ± 5.4	99.5 ± 5.7	102.4 ± 6.7
Waist: hip (ratio)	0.8 ± 0.05	0.79 ± 0.04	0.82 ± 0.07	0.81 ± 0.06	0.79 ± 0.04	0.83 ± 0.06
Fat free mass (kg)	52 ± 12	51 ± 7	51 ± 12	53 ± 14	50 ± 7	53 ± 12
Fat mass (kg)	18 ± 7	17 ± 7	20 ± 7	18 ± 8	17 ± 7	20 ± 7
Android fat mass (g)	1387 ± 900	1275 ± 746	1677 ± 797	1477 ± 1008	1267 ± 770	1769 ± 799
Gynoid fat mass (g)	3216 ± 1168	3119 ± 1259	3625 ± 1243	3216 ± 1135	3160 ± 1289	3641 ± 1314
Bone mineral density ($g\cdot cm^{-2}$)	1.13 ± 0.11	1.15 ± 0.06	1.16 ± 0.12	1.15 ± 0.13	1.15 ± 0.06	1.17 ± 0.12
Systolic blood pressure (mmHg)	119 ± 11	121 ± 7	117 ± 10	120 ± 11	121 ± 8	116 ± 10
Diastolic blood pressure (mmHg)	77 ± 6	77 ± 6	76 ± 5	79 ± 6	77 ± 7	75 ± 5
**Energy intake**						
Starch ($g\cdot d^{-1}$)	155 ± 74	161 ± 75	123 ± 59	155 ± 83	160 ± 77	123 ± 59
Fruit and vegetable sugars ($g\cdot d^{-1}$)	19 ± 13	19 ± 13	16 ± 9	20 ± 13	20 ± 13	18 ± 9
Milk sugars ($g\cdot d^{-1}$)	5 ± 6	4 ± 4	3 ± 5	2 ± 3	4 ± 4	2 ± 6
Liquid free sugars ($g\cdot d^{-1}$)	19 ± 26	14 ± 17	10 ± 9	20 ± 31	14 ± 18	10 ± 10
Solid free sugars ($g\cdot d^{-1}$)	52 ± 24	55 ± 29	44 ± 17	48 ± 24	57 ± 29	44 ± 13
Fat ($g\cdot d^{-1}$)	93 ± 36	82 ± 17	84 ± 27	93 ± 40	82 ± 18	87 ± 26
Protein ($g\cdot d^{-1}$)	91 ± 42	87 ± 24	89 ± 45	93 ± 49	87 ± 25	94 ± 45
Ethanol ($g\cdot d^{-1}$)	19 ± 18	10 ± 10	10 ± 12	18 ± 20	9 ± 10	10 ± 12
Fiber ($g\cdot d^{-1}$)	25 ± 9	26 ± 14	21 ± 8	26 ± 10	27 ± 14	23 ± 7
**Energy expenditure**						
PAEE ($kcal\cdot d^{-1}$)	1368 ± 677	1285 ± 515	1200 ± 674	1255 ± 716	1200 ± 399	1286 ± 733
Step count ($steps\cdot d^{-1}$)	9609 ± 4939	10485 ± 3680	8951 ± 5430	9983 ± 4976	10125 ± 3810	8951 ± 5430
RMR ($kcal\cdot d^{-1}$)	1671 ± 353	1637 ± 247	1651 ± 365	1665 ± 368	1634 ± 255	1694 ± 386
Protein oxidation ($g\cdot min^{-1}$)	0.02 ± 0.01	0.02 ± 0	0.02 ± 0.01	0.03 ± 0.01	0.02 ± 0	0.03 ± 0.01
Sleeping heart rate ($beats\cdot min^{-1}$)	49 ± 6	52 ± 8	52 ± 5	50 ± 6	53 ± 9	53 ± 4

Data are means ± SD.

Table S2. Baseline circulating biochemistry and muscle glycogen concentrations for participants following moderate-sugar control (MODSUG), low-sugar (LOWSUG), or low-carbohydrate diets (LOWCHO). Related to Figure 4.

	Week 4 completers			Week 12 completers		
	**MODSUG**	**LOWSUG**	**LOWCHO**	**MODSUG**	**LOWSUG**	**LOWCHO**
**Fasting**						
Total cholesterol (mmol·L^{-1})	4.77 ± 1.36	4.48 ± 1.19	4.52 ± 0.86	4.75 ± 1.62	4.65 ± 1.17	4.45 ± 0.85
HDL-C (mmol·L^{-1})	1.36 ± 0.42	1.4 ± 0.37	1.29 ± 0.25	1.27 ± 0.32	1.47 ± 0.37	1.26 ± 0.25
LDL-C (mmol·L^{-1})	2.7 ± 1.26	2.56 ± 1.04	2.49 ± 0.72	2.68 ± 1.44	2.62 ± 1.02	2.46 ± 0.68
ApoB (mg·dL^{-1})	82 ± 26	71 ± 24	73 ± 29	81 ± 30	72 ± 23	72 ± 27
Albumin (g·L^{-1})	41 ± 3	42 ± 3	42 ± 2	41 ± 3	42 ± 3	42 ± 2
WBC count (n·10^{9}·L^{-1})	4.9 ± 1.4	5.3 ± 1.3	8.1 ± 11.2	4.7 ± 1.5	5.3 ± 1.3	5.4 ± 1.2
RBC count (n·10^{12}·L^{-1})	4.6 ± 0.4	4.4 ± 0.4	4.5 ± 0.4	4.5 ± 0.3	4.4 ± 0.4	4.5 ± 0.4
Hemoglobin (g·dL^{-1})	13.9 ± 1.1	13.3 ± 1.3	13.4 ± 1.1	13.9 ± 0.9	13.3 ± 1.4	13.5 ± 1.1
Hematocrit (%)	39 ± 2	39 ± 3	39 ± 2	39 ± 2	38 ± 3	39 ± 2
Platelet count (n·10^{9}·L^{-1})	220 ± 49	229 ± 58	212 ± 48	211 ± 49	219 ± 43	207 ± 45
Leptin (ng·mL^{-1})	12 ± 11	14 ± 13	14 ± 14	13 ± 13	14 ± 13	14 ± 14
Ghrelin (pg·mL^{-1})	27 ± 25	13 ± 8	27 ± 27	18 ± 15	12 ± 7	27 ± 28
FGF21 (pg·mL^{-1})	155 ± 185	189 ± 265	247 ± 385	188 ± 212	204 ± 272	420 ± 1099
GLP-1 (pg·mL^{-1})	16 ± 7	19 ± 14	20 ± 8	13 ± 4	20 ± 14	20 ± 9
Acetate (μmol·L^{-1})	36 ± 33	27 ± 14	30 ± 15	32 ± 23	28 ± 17	30 ± 16
Propionate (μmol·L^{-1})	3.5 ± 2.3	3 ± 1.9	2.2 ± 1.2	3.2 ± 1.2	3.1 ± 0.5	1.6 ± 0.5
Butyrate (μmol·L^{-1})	0.55 ± 0.37	0.6 ± 0.43	0.51 ± 0.26	0.55 ± 0.37	0.54 ± 0.36	0.51 ± 0.28
CRP (mg·L^{-1})	0.83 ± 0.74	1.03 ± 1.11	0.91 ± 0.87	1.05 ± 0.85	0.95 ± 1.07	0.98 ± 0.92
LBP (mg·L^{-1})	11.2 ± 5.5	18 ± 8.4	15.3 ± 12.7	10.8 ± 6.1	17.7 ± 8.5	14.8 ± 13.2
**Postprandial**						
Glucose iAUC (mmol·L^{-1}·240 min)	188 ± 75	179 ± 156	195 ± 118	191 ± 84	177 ± 157	200 ± 119
Insulin iAUC (nmol·L^{-1}·240 min)	31 ± 22	36 ± 16	34 ± 18	32 ± 25	35 ± 17	34 ± 18
C-peptide iAUC (ng·mL^{-1}·240 min)	253 ± 161	259 ± 98	314 ± 129	271 ± 155	240 ± 97	330 ± 124
Lactate iAUC (mmol·L^{-1}·240 min)	66 ± 25	67 ± 30	61 ± 29	65 ± 23	68 ± 31	58 ± 23
βOHB AUC (mmol·L^{-1}·240 min)	18 ± 5	21 ± 10	20 ± 8	17 ± 3	21 ± 10	20 ± 8
TAG iAUC (mmol·L^{-1}·240 min)	53 ± 60	31 ± 23	42 ± 34	56 ± 65	30 ± 23	46 ± 35
NEFA AUC (mmol·L^{-1}·240 min)	56 ± 15	56 ± 18	57 ± 24	56 ± 15	57 ± 18	57 ± 25
Glycerol AUC (mmol·L^{-1}·240 min)	11 ± 9	8 ± 7	10 ± 8	8 ± 7	8 ± 7	10 ± 8
Ghrelin AUC (pg·mL^{-1}·240 min)	4920 ± 2979	3755 ± 2666	7762 ± 6611	3993 ± 1714	3357 ± 2350	8271 ± 6763
FGF21 AUC (pg·mL^{-1}·240 min)	29 ± 45	27 ± 33	36 ± 41	36 ± 53	29 ± 34	58 ± 125
iAUC GLP-1 (pg·mL^{-1}·240 min)	1609 ± 872	1450 ± 1206	1558 ± 1462	1763 ± 940	1422 ± 1190	1577 ± 1478

**CGMS**						
Mean glucose (mmol·L^{-1})	4.8 ± 0.5	4.7 ± 0.6	4.6 ± 0.3	5 ± 0.3	4.5 ± 0.4	4.6 ± 0.3
Glucose CV (%)	10.9 ± 3.4	11.6 ± 2.4	12.3 ± 5.3	11 ± 3.9	11.2 ± 2.8	11.6 ± 5.4
Glucose SD (mmol·L^{-1})	0.5 ± 0.1	0.5 ± 0	0.5 ± 0.2	0.5 ± 0.1	0.5 ± 0.1	0.5 ± 0.2
**Skeletal muscle**						
Glycogen (mmol·kg dm^{-1})	278 ± 78	254 ± 140	276 ± 77	265 ± 76	220 ± 155	296 ± 76

Data are means ± SD.

Table S3. Self-reported daily nutrient intake during 12 weeks of moderate-sugar diet (MODSUG), low-sugar diet (LOWSUG), or low-carbohydrate diet (LOWCHO). Related to Figure 1.

	Weeks 1 to 4			Weeks 5 to 12		
	**MODSUG**	**LOWSUG**	**LOWCHO**	**MODSUG**	**LOWSUG**	**LOWCHO**
Starch ($g \cdot d^{-1}$)	151 ± 68	153 ± 60	11 ± 5	144 ± 65	160 ± 64	15 ± 6
Fruit and vegetable sugars ($g \cdot d^{-1}$)	17 ± 12	19 ± 15	6 ± 2	17 ± 12	19 ± 18	6 ± 2
Milk sugars ($g \cdot d^{-1}$)	4 ± 4	3 ± 3	0 ± 2	3 ± 4	4 ± 4	0 ± 1
Liquid free sugars ($g \cdot d^{-1}$)	33 ± 23	2 ± 3	0 ± 1	35 ± 22	2 ± 4	1 ± 1
Solid free sugars ($g \cdot d^{-1}$)	67 ± 23	17 ± 6	8 ± 2	64 ± 29	19 ± 7	11 ± 4
Fat ($g \cdot d^{-1}$)	94 ± 37	87 ± 18	135 ± 51	89 ± 40	80 ± 15	145 ± 54
Protein ($g \cdot d^{-1}$)	91 ± 42	86 ± 22	96 ± 32	86 ± 32	84 ± 24	97 ± 39
Ethanol ($g \cdot d^{-1}$)	10 ± 8	7 ± 9	5 ± 8	11 ± 9	7 ± 8	4 ± 6
Fiber ($g \cdot d^{-1}$)	26 ± 9	26 ± 11	14 ± 5	23 ± 9	26 ± 11	15 ± 6

Data are means ± SD.

Table S4. Resting metabolic rate, protein oxidation, sleeping heart rate, fasting blood pressure and daily step count at week 4, and week 12 of moderate-sugar diet (MODSUG), low-sugar diet (LOWSUG), or low-carbohydrate diet (LOWCHO) in healthy men and women. Related to Figure 1.

	Week 4			Week 12		
	MODSUG	LOWSUG	LOWCHO	MODSUG	LOWSUG	LOWCHO
Resting metabolic rate ($g \cdot min^{-1}$)	1658 (1597 to 1719)	1674 (1611 to 1737)	1609 (1548 to 1670)	1653 (1498 to 1807)	1594 (1450 to 1738)	1685 (1545 to 1824)
Resting metabolic $rate_{adjFFM}$ ($g \cdot min^{-1}$)	1653 (1591 to 1715)	1669 (1605 to 1732)	1620 (1555 to 1684)	1641 (1484 to 1799)	1614 (1456 to 1772)	1687 (1543 to 1831)
Protein oxidation ($g \cdot min^{-1}$)	0.025 (0.018 to 0.032)	0.032 (0.025 to 0.038)	0.033 (0.027 to 0.040)	0.030 (0.019 to 0.041)	0.028 (0.019 to 0.037)	0.036 (0.027 to 0.045)
Sleeping heart rate ($beats \cdot min^{-1}$)	52 (51 to 54)	51 (49 to 52)	53 (51 to 55)	53 (51 to 55)	51 (50 to 53)	55[b] (54 to 57)
Systolic blood pressure (mmHg)	117 (114 to 120)	115 (112 to 117)	115 (112 to 118)	116 (112 to 120)	116 (113 to 120)	119 (116 to 123)
Diastolic blood pressure (mmHg)	75 (73 to 78)	76 (73 to 78)	75 (73 to 78)	76 (74 to 79)	76 (74 to 79)	76 (74 to 78)
Step count ($steps \cdot d^{-1}$)	8589 (7102 to 10075)	8835 (7341 to 10329)	9330 (7717 to 10942)	8634 (7324 to 9943)	8242 (6931 to 9552)	9109 (7640 to 10579)

Data are mean (95% CI) from ANCOVA with baseline values as the covariate ($_{adjFFM}$ indicates the change in FFM also added as a covariate). Step count for week 4 reflect the average over weeks 1 to 4 and for week 12 reflect the average over weeks 5 to 12.

Table S5. Body composition following 4 weeks and 12 weeks of moderate-sugar diet (MODSUG), low-sugar diet (LOWSUG), or low-carbohydrate diet (LOWCHO) in healthy men and women. Related to Figure 2.

	Week 4			Week 12		
	MODSUG	LOWSUG	LOWCHO	MODSUG	LOWSUG	LOWCHO
Waist:hip (ratio)	0.81 (0.80 to 0.82)	0.81 (0.80 to 0.82)	0.79 (0.78 to 0.80)	0.82 (0.80 to 0.84)	0.82 (0.81 to 0.84)	0.80 (0.79 to 0.82)
Fat free mass (kg)	51.8 (51.3 to 52.4)	51.8 (51.3 to 52.4)	50.7[b] (50.3 to 51.4)	52.0 (51.2 to 52.9)	52.6 (51.8 to 53.4)	51.4 (50.7 to 52.2)
Fat mass (kg)	18.8 (18.4 to 19.2)	17.8[a] (17.4 to 18.3)	17.2[b] (16.8 to 17.6)	19.5 (18.6 to 20.3)	17.3[a] (16.6 to 18.1)	16.6[b] (15.8 to 17.3)
Android fat mass (g)	1455 (1387 to 1526)	1335[a] (1268 to 1402)	1312[b] (1247 to 1377)	1593 (1473 to 1714)	1337[a] (1235 to 1438)	1269[b] (1168 to 1371)
Gynoid fat mass (g)	3296 (3205 to 3387)	3170 (3082 to 3259)	3085[b] (2998 to 3172)	3427 (3266 to 3588)	3001[a] (2867 to 3134)	2963[b] (2828 to 3098)
Bone mineral density ($g \cdot cm^{-2}$)	1.15 (1.14 to 1.16)	1.15 (1.14 to 1.16)	1.15 (1.14 to 1.16)	1.18 (1.16 to 1.20)	1.16 (1.14 to 1.18)	1.16 (1.15 to 1.18)

Data shown are ANCOVA-adjusted mean (95% CI) for Week 4 and Week 12 (with baseline values as the covariate). [a]$p < 0.05$ LOWSUG vs MODSUG, [b]$p < 0.05$ LOWCHO vs MODSUG. Week 4 n=53, Week 12 n=45. DXA-derived measures of tissue mass and bone density.

Table S6. Fasting circulating biochemical and hematology markers following 4 weeks and 12 weeks of moderate-sugar diet (MODSUG), low-sugar diet (LOWSUG), or low-carbohydrate diet (LOWCHO) in healthy men and women. Related to Figure 3.

	Week 4			Week 12		
	MODSUG	LOWSUG	LOWCHO	MODSUG	LOWSUG	LOWCHO
Fasting glucose (mmol·L^{-1})	5.44 (5.21 to 5.68)	5.20 (4.96 to 5.45)	4.98[b] (4.74 to 5.21)	5.83 (5.49 to 6.18)	5.39 (5.11 to 5.68)	5.51 (5.22 to 5.79)
Fasting insulin (pmol·L^{-1})	29 (24 to 34)	28 (23 to 34)	39 (24 to 34)	33 (26 to 40)	25 (20 to 31)	27 (21 to 32)
Fasting c-peptide (pg·mL^{-1})	549 (450 to 649)	600 (497 to 702)	496 (395 to 596)	564 (445 to 683)	596 (496 to 695)	538 (438 to 638)
Fasting lactate (mmol·L^{-1})	1.00 (0.87 to 1.12)	0.99 (0.86 to 1.12)	0.94 (0.81 to 1.06)	1.03 (0.87 to 1.19)	0.88 (0.75 to 1.02)	0.90 (0.77 to 1.04)
Fasting βOHB (mmol·L^{-1})	0.12 (0.00 to 0.39)	0.23 (0.00 to 0.50)	1.28[b] (1.01 to 1.54)	0.14 (0.00 to 0.37)	0.21 (0.02 to 0.40)	0.82[b] (0.62 to 1.01)
Fasting FGF21 (pg·mL^{-1})	196 (116 to 277)	170 (87 to 253)	97 (14 to 180)	196 (139 to 254)	138 (90 to 185)	94[b] (44 to 143)
Fasting ghrelin (pg·mL^{-1})	18.2 (14.1 to 22.2)	22.1 (17.8 to 26.4)	19.4 (15.5 to 23.4)	21.7 (17.7 to 25.6)	24.4 (20.9 to 27.8)	18.0 (14.7 to 21.4)
Fasting albumin (g·L^{-1})	41.6 (40.4 to 42.8)	41.9 (40.7 to 43.1)	41.7 (40.5 to 42.8)	43.0 (40.5 to 45.4)	42.1 (40.1 to 44.1)	43.0 (40.9 to 45.0)
Fasting TAG (mmol·L^{-1})	0.74 (0.59 to 0.88)	0.74 (0.59 to 0.89)	0.90 (0.76 to 1.05)	0.68 (0.43 to 0.92)	0.71 (0.52 to 0.90)	0.93 (0.74 to 1.12)
Fasting NEFA (mmol·L^{-1})	0.49 (0.40 to 0.59)	0.53 (0.43 to 0.63)	0.70[b] (0.61 to 0.80)	0.58 (0.46 to 0.71)	0.54 (0.44 to 0.65)	0.62[b] (0.52 to 0.72)
Fasting glycerol (mmol·L^{-1})	0.04 (0.04 to 0.05)	0.05 (0.04 to 0.06)	0.06 (0.05 to 0.07)	0.04 (0.03 to 0.06)	0.04 (0.03 to 0.05)	0.05 (0.04 to 0.07)
Fasting total cholesterol (mmol·L^{-1})	4.6 (4.3 to 4.8)	4.3 (4.0 to 4.5)	5.0* (4.8 to 5.3)	4.9 (4.5 to 5.2)	4.2* (3.9 to 4.5)	5.2 (4.9 to 5.5)
Fasting LDL-C (mmol·L^{-1})	2.7 (2.5 to 2.9)	2.3* (2.1 to 2.5)	2.9 (2.7 to 3.2)	2.9 (2.5 to 3.2)	2.3* (2.1 to 2.6)	3.0 (2.7 to 3.1)
Fasting HDL-C (mmol·L^{-1})	1.3 (1.2 to 1.4)	1.3 (1.2 to 1.4)	1.3 (1.2 to 1.4)	1.4 (1.2 to 1.5)	1.3 (1.2 to 1.4)	1.4 (1.3 to 1.5)
Fasting apoB (mg·dL^{-1})	75 (67 to 83)	71 (63 to 79)	92[b] (84 to 100)	80 (67 to 94)	74 (63 to 85)	89 (78 to 100)
Adipo-IR (mmol·L^{-1} x pmol·L^{-1})	13.0 (9.1 to 16.9)	15.3 (11.3 to 19.3)	21.3[b] (17.5 to 25.2)	18.6 (13.8 to 23.4)	14.4 (10.5 to 18.4)	15.6 (11.6 to 19.6)
WBC count (n·10^{9}·L^{-1})	5.2 (4.8 to 5.7)	4.7 (4.3 to 5.1)	4.5 (4.1 to 4.9)	5.1 (4.5 to 5.7)	5.1 (4.6 to 5.6)	4.7 (4.2 to 5.1)
RBC count (n·10^{12}·L^{-1})	4.42 (4.31 to 4.54)	4.38 (4.26 to 4.49)	4.49 (4.38 to 4.60)	4.52 (4.40 to 4.64)	4.38 (4.28 to 4.49)	4.45 (4.35 to 4.55)
Hemoglobin (g·dL^{-1})	13.2 (12.9 to 13.6)	13.2 (12.9 to 13.6)	13.6 (13.2 to 13.9)	13.7 (13.3 to 14.1)	13.1 (12.7 to 13.5)	13.4 (13.1 to 13.8)
Hematocrit (%)	38.7 (37.6 to 39.8)	38.6 (37.5 to 39.7)	39.0 (38.0 to 40.1)	39.4 (38.3 to 40.5)	38.0 (37.1 to 38.9)	38.8 (37.9 to 39.7)
Platelet count (n·10^{9}·L^{-1})	227 (213 to 240)	208 (195 to 222)	210 (198 to 223)	216 (195 to 237)	216 (197 to 234)	205 (187 to 222)

Data shown are ANCOVA-adjusted mean (95% CI) for Week 4 and Week 12. [a]$p < 0.05$ LOWSUG vs MODSUG, [b]$p < 0.05$ LOWCHO vs MODSUG. βOHB = beta-hydroxybutyrate, FGF21 = fibroblast growth factor-21, TAG = triglyceride, NEFA = non-esterified fatty acid, WBC = white blood cell, RBC = red blood cell. Week 4 n=52, week 12 n=43.

Table S7. Outcomes from continuous glucose monitoring during at baseline, week 1, and week 11-12 of moderate-sugar diet (MODSUG), low-sugar diet (LOWSUG), or low-carbohydrate diet (LOWCHO) in healthy men and women. Related to Figure 3.

	**Week 1**			**Week 12**		
	MODSUG	LOWSUG	LOWCHO	MODSUG	LOWSUG	LOWCHO
Wear time (days)	5 (1)	5 (1)	6 (1)	10 (3)	11 (2)	11 (3)
Mean glucose ($mmol \cdot L^{-1}$)	4.5 (4.4 to 4.7)	4.7 (4.5 to 4.8)	4.1[b] (4.0 to 4.2)	4.8 (4.3 to 5.2)	4.8 (4.4 to 5.3)	4.5 (4.2 to 4.8)
Glucose CV (%)	18.3 (16.0 to 20.5)	15.3 (12.9 to 17.7)	13.8[b] (11.7 to 15.9)	18.4 (16.3 to 20.6)	16.7 (14.4 to 19.0)	15.8 (14.2 to 17.5)
Glucose management indicator (%)	5.3 (5.2 to 5.3)	5.3 (5.3 to 5.4)	5.1[b] (5.0 to 5.1)	5.4 (5.2 to 5.6)	5.4 (5.2 to 5.6)	5.2 (5.1 to 5.4)
Time spent above 13.9 $mmol \cdot L^{-1}$ (%)	0 (0 to 0)	0 (0 to 0)	0 (0 to 0)	0 (0 to 0)	0 (0 to 0)	0 (0 to 0)
Time spent between 13.9 and 10.1 $mmol \cdot L^{-1}$ (%)	0 (0 to 0)	0 (0 to 0)	0 (0 to 0)	1 (0 to 2)	0 (0 to 0)	0 (0 to 0)
Time spent between 10.0 and 3.9 $mmol \cdot L^{-1}$ (%)	84 (71 to 97)	86 (76 to 95)	58 (45 to 70)	88 (80 to 97)	82 (67 to 97)	74 (62 to 86)
Time spent between 3.8 and 3.0 $mmol \cdot L^{-1}$ (%)	11 (5 to 17)	14 (4 to 24)	38 (27 to 49)	10 (2 to 19)	16 (3 to 30)	21 (12 to 30)
Time spent below 3.0 $mmol \cdot L^{-1}$ (%)	5 (0 to 14)	0 (0 to 0)	4 (2 to 6)	1 (0 to 1)	2 (0 to 3)	5 (1 to 8)

Wear time is mean (SD). Mean glucose, glucose CV, and glucose management indicator are ANCOVA-adjusted mean (95% CI) for Week 4 and Week 12 (with baseline values as the covariate). Time spent in range are unadjusted mean (95% CI) for all time-points. [a]$p < 0.05$ LOWSUG vs MODSUG, [b]$p < 0.05$ LOWCHO vs MODSUG. Week 1 n=39, Week 12 n=32.

Table S9. Nutrition information of foods included in the alternative forced choice task used to measure relative food preference. Related to Figure 7.

Description	Energy density (kcal·g^{-1})	Fat (g per 100 g)	CHO (g per 100 g)	Sugars (g per 100 g)	Protein (g per 100 g)
High-carbohydrate sweet					
Banana	1.03	0.5	23.2	20.9	1.2
Strawberry trifle	1.47	6.0	21.0	19.3	2.0
Toffee and vanilla ice cream	1.84	6.5	28.3	22.4	2.8
Apple pie	2.70	10.5	40.0	21.1	2.7
Chocolate on toast	3.46	12.5	49.8	21.5	7.4
Chocolate digestive biscuits	4.95	23.6	62.2	29.5	6.7
High-carbohydrate savory					
Rice with sweetcorn	1.17	1.2	22.6	1.2	4.1
Tomato and basil pasta	1.46	4.1	22.7	4.6	3.7
Noodles with katsu sauce	1.86	6.2	29.0	4.7	2.7
French fries	2.47	9.6	36.1	0.9	2.4
Garlic bread	3.39	13.0	44.8	2.7	9.7
Cheese crackers	5.00	23.0	63.5	8.8	7.7
Low-carbohydrate savory					
Vegetarian chicken slices	1.19	2.6	4.5	0.9	16.3
Hard-boiled egg	1.43	9.6	0.5	0.5	14.1
Tuna and mayonnaise	1.86	11.4	0.5	0.2	20.1
Vegetarian bacon slices	2.14	15.7	5.4	2.6	10.1
Edam cheese	3.12	24.0	0.5	0.5	24.0
Peanuts and edamame beans	4.70	37.4	7.2	4.8	21.7

Table S10. RNA expression primer sequences. Related to Figure 6.

primers		Sequence (5'-3')
FABP4	Forward	ACTGGGCCAGGAATTTGACG
	Reverse	CTCGTGGAAGTGACGCCTT
IRS1	Forward	AGTCTGTCGTCCAGTAGCACCA
	Reverse	ACTGGAGCCATACTCATCCGAG
IRS2	Forward	CCACCATCGTGAAAGAGTGAAGA
	Reverse	CTGAAACAGTGCTGAGCGTC
TBC1D4	Forward	ATGAGAGGTCGGCTTGGAAGTG
	Reverse	CGGAATCCTCTTCGGGAAACGT
GLUT4	Forward	ACTGGACGAGCAACTTCATC
	Reverse	GAGGACCGCAAATAGAAGGAA
TBC1D1	Forward	CACCCAGTGCCACTCGATTT
	Reverse	TGGCTTTATTACCCCGGGAC
Akt 1	Forward	CGAGCTGTTCTTCCACCTGT
	Reverse	TAATGTGCCCGTCCTTGTCC
Akt 2	Forward	CTGCGGAAGGAAGTCATCATT
	Reverse	GGTCGTGGGTCTGGAAGG
INSR	Forward	CGTCCCCAGAAAAACCTCTTC
	Reverse	ACGGCCACCGTCACATTC
PDK4	Forward	TGGAGCATTTCTCGCGCTAC
	Reverse	ACAGGCAATTCTTGTCGCAAA
PPIA	Forward	CCCACCGTGTTCTTCGACATT
	Reverse	GGACCCGTATGCTTTAGGATGA
Leptin	Forward	TTTTGTCAAGTGTCATATGTAGGTGTC
	Reverse	CTCCCTTCTGCCCAAACATTC
Adiponectin	Forward	TGCCCAAAGAGGAGAGAGGAA
	Reverse	TCAGAAACAGGCACACAACTCA
SREBP1c	Forward	TTCTGACAGCCATGAAGACAG
	Reverse	CCGCATCTACGACCAGTG
HSL	Forward	TCAGTGTCTAGGTCAGACTGG
	Reverse	AGGCTTCTGTTGGGTATTGGA
FAS	Forward	CAGAGTCGGAGAACTTGCAG
	Reverse	GGAGGCATCAAACCTAGACAG
Actin	Forward	CCTCGCCTTTGCCGATCC
	Reverse	CATCACGCCCTGGTGCC
18S	Forward	AACCCGTTGAACCCCATT
	Reverse	CCATCCAATCGGTAGTAGCG
LPL	Forward	GGACTGAGAGTGAAACCCATAC
	Reverse	GGAAGGAGTAGGTCTTATTTGTGG
PPARɣ	Forward	GATCCAGTGGTTGCAGAT
	Reverse	GAGGGAGTTGGAAGGCTCTTC
CPT1A	Forward	TCCAGTTGGCTTATCGTGGTG
	Reverse	CTAACGAGGGGTCGATCTTGG
GPR109A/HCAR2	Forward	GCCGATCCAGAATGGCGG
	Reverse	TTCGTGCCACTGGAAGGTAT
