# Supplementary material for: Ketogenic diet but not free-sugar restriction alters glucose tolerance, lipid metabolism, peripheral tissue phenotype, and gut microbiome: RCT
Source: Cell Rep Med. 2024 Aug 5;5(8):101667. doi: 10.1016/j.xcrm.2024.101667 (PMC11384946; doi:10.1016/j.xcrm.2024.101667)
Supplement: Document S2. Article plus supplemental information [file mmc3.pdf]

Cell Reports Medicine

Article

# Ketogenic diet but not free-sugar restriction alters glucose tolerance, lipid metabolism, peripheral tissue phenotype, and gut microbiome: RCT

## Graphical abstract

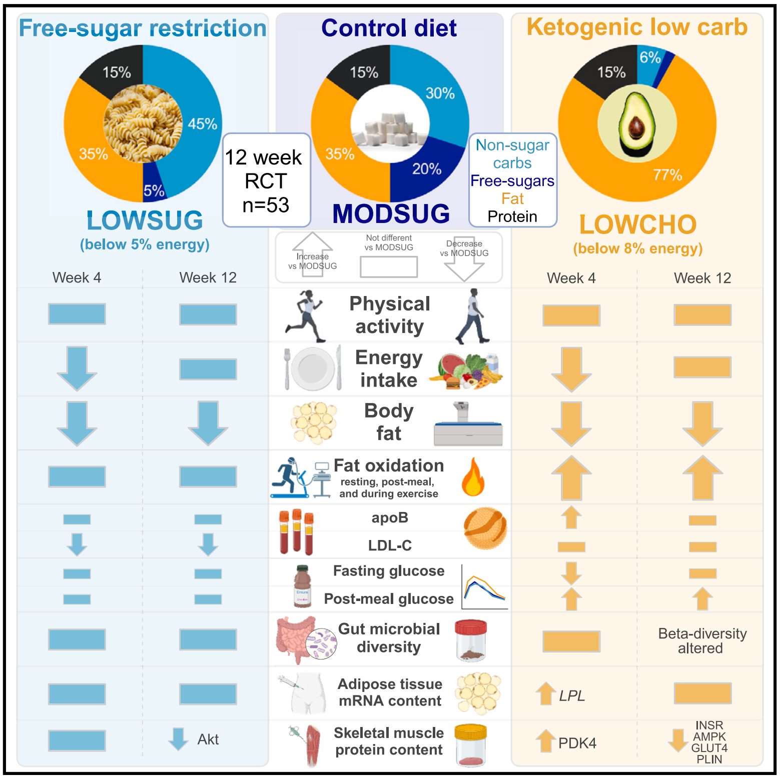


## Highlights

- Low free-sugar or carbohydrate intake reduces body fat but not energy expenditure
- Restricting sugar lowers LDL-C with minimal impact on metabolism or gut microbiome
- Four weeks of a ketogenic diet increases atherogenic lipoprotein profile
- The ketogenic diet alters gut microbial beta diversity and skeletal muscle phenotype

## Authors

Aaron Hengist, Russell G. Davies, Jean-Philippe Walhin, ..., Françoise Koumanov, Dylan Thompson, Javier T. Gonzalez

## Correspondence

J.T.Gonzalez@bath.ac.uk

## In brief

Hengist et al. show that restricting free sugars or overall carbohydrates (ketogenic diet) both reduce body fat over 12 weeks in healthy adults. While sugar restriction had relatively little effect on metabolism or the gut microbiome, ketogenic carbohydrate restriction increases atherogenic lipoproteins, reduces glucose tolerance, and alters gut microbial diversity.

Hengist et al., 2024, Cell Reports Medicine *5*, 101667
August 20, 2024 © 2024 The Author(s). Published by Elsevier Inc.
https://doi.org/10.1016/j.xcrm.2024.101667

CellPress

Cell Reports Medicine

CellPress OPEN ACCESS

Article

# Ketogenic diet but not free-sugar restriction alters glucose tolerance, lipid metabolism, peripheral tissue phenotype, and gut microbiome: RCT

Aaron Hengist,[1,9,10] Russell G. Davies,[1,9] Jean-Philippe Walhin,[1] Jariya Buniam,[1,2] Lucy H. Merrell,[1] Lucy Rogers,[1] Louise Bradshaw,[1] Alfonso Moreno-Cabañas,[1] Peter J. Rogers,[3] Jeff M. Brunstrom,[3] Leanne Hodson,[4] Luc J.C. van Loon,[5] Wiley Barton,[6,7,8] Ciara O'Donovan,[6,7] Fiona Crispie,[6,7] Orla O'Sullivan,[6,7,8] Paul D. Cotter,[6,7,8] Kathryn Proctor,[1] James A. Betts,[1] Françoise Koumanov,[1] Dylan Thompson,[1] and Javier T. Gonzalez[1,10,11,12,*]

[1]University of Bath, Bath, UK
[2]Chulabhorn Royal Academy, Bangkok, Thailand
[3]University of Bristol, Bristol, UK
[4]University of Oxford and National Institute for Health Research Oxford Biomedical Research Centre, Oxford University Hospital Trusts, Oxford, UK
[5]Maastricht University, Maastricht, the Netherlands
[6]Teagasc Food Research Centre, Moorepark, Cork, Ireland
[7]APC Microbiome Ireland, Cork, Ireland
[8]VistaMilk, Cork, Ireland
[9]These authors contributed equally
[10]Senior author
[11]X (formerly Twitter): @Gonzalez_JT
[12]Lead contact
*Correspondence: J.T.Gonzalez@bath.ac.uk
https://doi.org/10.1016/j.xcrm.2024.101667

## SUMMARY

Restricted sugar and ketogenic diets can alter energy balance/metabolism, but decreased energy intake may be compensated by reduced expenditure. In healthy adults, randomization to restricting free sugars or overall carbohydrates (ketogenic diet) for 12 weeks reduces fat mass without changing energy expenditure versus control. Free-sugar restriction minimally affects metabolism or gut microbiome but decreases low-density lipoprotein cholesterol (LDL-C). In contrast, a ketogenic diet decreases glucose tolerance, increases skeletal muscle PDK4, and reduces AMPK and GLUT4 levels. By week 4, the ketogenic diet reduces fasting glucose and increases apolipoprotein B, C-reactive protein, and postprandial glycerol concentrations. However, despite sustained ketosis, these effects are no longer apparent by week 12, when gut microbial beta diversity is altered, possibly reflective of longer-term adjustments to the ketogenic diet and/or energy balance. These data demonstrate that restricting free sugars or overall carbohydrates reduces energy intake without altering physical activity, but with divergent effects on glucose tolerance, lipoprotein profiles, and gut microbiome.

## INTRODUCTION

Free sugars are "monosaccharides and disaccharides added to foods by the manufacturer, cook, or consumer, plus sugars naturally present in honey, syrups, and unsweetened fruit juices."[1] This encompasses added sugars and sugars naturally present in fruit and vegetable juices, concentrates, smoothies, purées, pastes, powders, and extruded products.[2,3] In randomized trials, greater proportional free-sugar consumption increases self-reported energy intake, and reduction of free sugars to ≤5% of energy intake has been theorized to reduce energy intake by 100 kcal/day.[1,4] However, dietary free-sugar manipulation results in small changes in fat mass (FM) compared to the self-reported changes in energy intake,[5] suggesting other components of energy balance are compensating for this effect, or that reporting of energy intake is inaccurate. Dietary guidelines advocate restricting free (or added) sugars from current intakes almost entirely due to the basis of effects on self-reported energy intake.[1,6,3] There is, therefore, a need to establish whether restriction of free sugars can meaningfully alter energy intake without reliance on self-report methods. We previously reported that free-sugar restriction does not modulate energy balance components within 24 h.[7] To date, no study has directly measured physical activity energy expenditure (PAEE) or objectively determined energy intake in response to longer-term free-sugar restriction to infer efficacy or effectiveness of recommendations. Furthermore, the impact of free-sugar restriction and associated body composition changes on metabolism and gut microbiome is currently unclear.


This is an open access article under the CC BY license (http://creativecommons.org/licenses/by/4.0/).

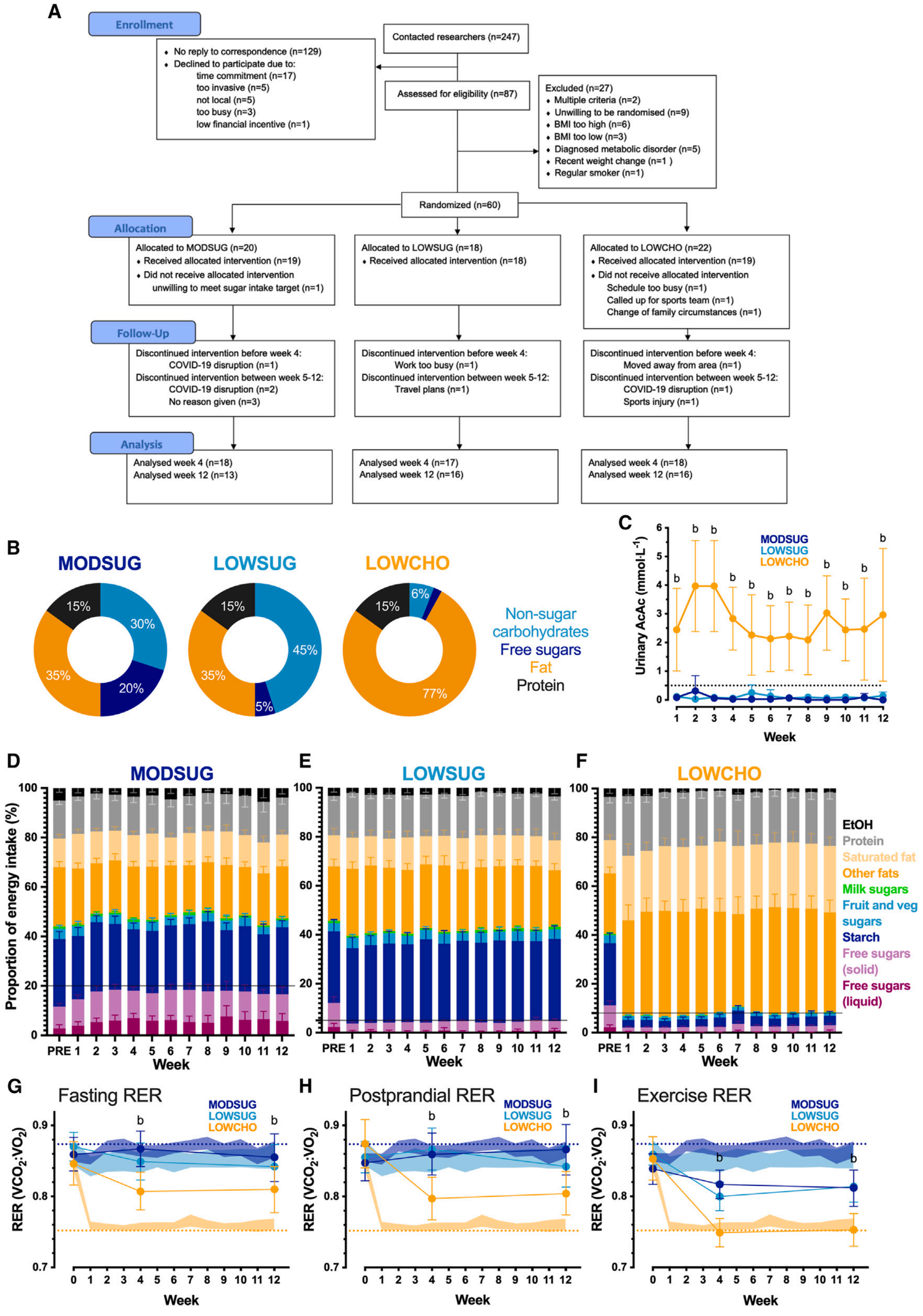


(legend on next page)

Restricting overall carbohydrate intake is another strategy often utilized to alter body composition, but with additional metabolic effects.[8] With low carbohydrate availability (ketogenic diet), hepatically produced ketone bodies provide an alternative fuel to carbohydrates for organs like the brain and skeletal muscle.[9,10] The effects of ketogenic diets on objectively measured energy balance components and cardiometabolic health in humans are not well characterized.[11,12] Only one pilot study has indirectly estimated PAEE in response to ketogenic diets, suggesting reduced PAEE on a ketogenic diet (7% carbohydrate, 83% fat) compared with a high-carbohydrate diet (83% carbohydrate, 7% fat),[13] with five participants measured across 9–21 days of diet, and PAEE estimated by subtracting resting metabolic rate from total energy expenditure. As such, it is important to investigate the effects of ketogenic carbohydrate restriction on energy balance and metabolism, while objectively measuring potentially major compensatory components such as physical activity.

In addition to effects on energy balance, physical activity has independent effects on cardiometabolic health. Peripheral tissue phenotype (e.g., skeletal muscle and adipose tissue) plays a crucial role in regulating fasting and postprandial metabolism and is influenced by nutrition and physical activity, with further modulation by changes in gut microbiome composition (e.g., via production of short-chain fatty acids [SCFAs]).[14,15] Therefore, peripheral tissue phenotype may provide key links between any effects of diet and associated changes in physical activity on cardiometabolic health. Combinations of carbohydrate restriction with low energy availability, like skipping breakfast[16] and intermittent 24-h fasting,[17] can reduce objectively measured PAEE, with potential implications for cardiometabolic health. The role of carbohydrate manipulation per se (i.e., independent of total energy intake) on PAEE is yet to be elucidated. Therefore, the primary aim of this study was to characterize the effects of restricting dietary free sugars (LOWSUG), or overall carbohydrates (ketogenic diet; LOWCHO), on free-living PAEE over 12 weeks in healthy adult humans. These two diets were each compared to a control diet with a moderate sugar and moderate carbohydrate content (MODSUG), and total energy intake was free to vary in all conditions (i.e., proportional macronutrient intake is the intervention). We hypothesized that ketogenic carbohydrate restriction and free-sugar restriction would reduce physical activity compared with the control diet. Fasting and postprandial metabolism, appetite-related hormones, skeletal muscle and adipose tissue phenotypes, gut microbiome composition, and objectively measured energy intake were explored to establish cardiometabolic responses and underlying mechanisms in response to diets restricting free sugars or total carbohydrate intake (ketogenic).

## RESULTS

Sixty healthy adult participants were randomized, and 53 participants completed $\geq 4$ weeks of either MODSUG ($n$ = 18), LOWSUG ($n$ = 17), or LOWCHO ($n$ = 18) diet (Figures 1A and 1B). Baseline characteristics, habitual diet, and energy expenditure measures are reported in Table S1. Baseline circulating biochemical profiles, diurnal glycemia, and muscle glycogen concentrations are reported in Table S2. The study was conducted under almost complete free-living conditions, with only 3.5% of time spent within the laboratory.

### The interventions effectively manipulated nutrient intake, altered substrate oxidation, and achieved ketosis

Urinary acetoacetate concentrations were elevated throughout the 12-week intervention in LOWCHO ($p < 0.01$ vs. MODSUG; Figure 1C). Participants reported consuming ~18% of energy from free sugars in the MODSUG group (Figure 1D), whereas participants reported restricting free sugars to the target of <5% energy in the LOWSUG group (Figure 1E). The LOWCHO group reported restricting total carbohydrates to <8% energy (Figure 1F). Absolute nutrient intakes from self-report weighted food diaries are displayed in Table S3.

At week 4, carbohydrate restriction lowered respiratory exchange ratio (RER) in the fasted state ($p$ = 0.004; Figure 1G), postprandial state ($p$ = 0.005; Figure 1H), and during exercise ($p < 0.001$; Figure 1I). At week 12, these reductions in RER were still present with carbohydrate restriction in the fasted state ($p$ = 0.04; Figure 1H), postprandial state ($p$ = 0.01; Figure 1H), and during exercise ($p$ = 0.001; Figure 1I). Sugar restriction did not meaningfully alter RER in any of the states measured at either week 4 or week 12 (all $p > 0.29$ vs. MODSUG). Incremental treadmill exercise data are displayed in Figure S1. Protein oxidation was negligible in all conditions and was not altered by intervention (all $p > 0.08$; Table S4).

### Neither sugar nor total carbohydrate restriction meaningfully altered physical activity or other components of energy expenditure, but they did reduce energy intake and thereby altered body composition

As carbohydrate restriction altered the relationship between energy expenditure and heart rate, we quantified free-living PAEE using heart rate and branched equation modeling[18,19] with individual-level calibration against the gold-standard laboratory assessments.[20] At week 4, compared to MODSUG, there was no evidence of meaningful differences in PAEE with either sugar

**Figure 1. CONSORT diagram, macronutrient targets, and biomarkers of adherence to moderate sugar, free-sugar-restricted, and ketogenic carbohydrate-restricted diets**

(A–I) CONSORT diagram of flow through study from enrollment to allocation and analysis of participants randomized (A). Prescribed dietary macronutrient targets (%total energy) for intervention arms (B). Weekly morning fasted urinary acetoacetate (AcAc) concentrations (C; $n$ = 33–55). Self-reported proportional nutrient composition of the moderate sugar (MODSUG) group with horizontal line marking 20% as the target intake for free sugars (D; $n$ = 10–18), the free-sugar-restricted (LOWSUG) group with horizontal line marking 5% as the target for maximal intake of free sugars (E; $n$ = 15–17), and the ketogenic carbohydrate-restricted (LOWCHO) group with the horizontal line marking 8% as the target for maximal intake of overall carbohydrates (F; $n$ = 16–18). Respiratory exchange ratio (RER; filled dots), prescribed food quotients (dotted lines) and self-reported food quotients (shaded area = 95% CI) fasting (G; $n$ = 45–53), postprandial (H; $n$ = 42–48), and during treadmill walking test following a 4–5 h fast (I; $n$ = 45–53). Data for B–F are unadjusted mean (95% CI). Data for G–I are mean (95% CI) at baseline, and ANCOVA-adjusted mean (95% CI) during the interventions (with baseline as the covariate). [a]$p \leq 0.05$ for LOWSUG vs. MODSUG; [b]$p \leq 0.05$ for LOWCHO vs. MODSUG.

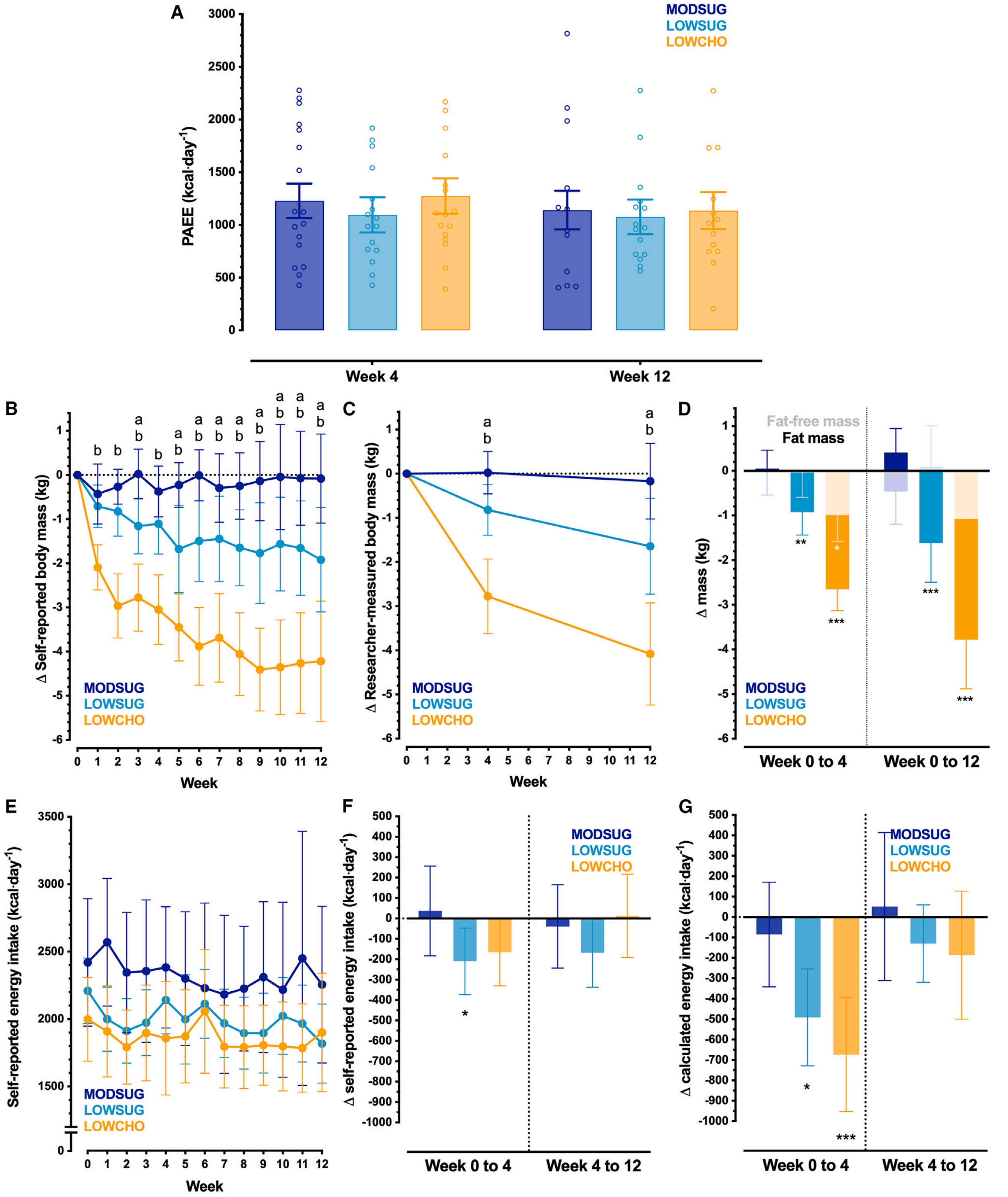


**Figure 2. Physical activity, changes in body mass and composition, and energy intake from self-report and objectively calculated methods across 12 weeks of MODSUG, LOWSUG, or LOWCHO diets**

(A–G) Baseline-adjusted (ANCOVA) mean (95% CI) and individual 24-h physical activity energy expenditure (PAEE) at week 4 and week 12 (A). Self-reported change in body mass across 12 weeks (B), researcher-measured change in body mass across 12 weeks (C), and DXA-derived changes in fat mass (FM; solid

*(legend continued on next page)*

restriction ($-133$ kcal days$^{-1}$; 95% confidence interval [CI]: $-366$ to 100 kcal days$^{-1}$ vs. MODSUG, $p = 0.26$) or carbohydrate restriction (46 kcal days$^{-1}$; 95% CI: $-189$ to 280 kcal days$^{-1}$ vs. MODSUG, $p = 0.70$). At week 12, there were no substantial changes in these differences with sugar restriction ($-65$ kcal days$^{-1}$; 95% CI: $-310$ to 180 kcal days$^{-1}$ vs. MODSUG, $p = 0.60$) or carbohydrate restriction ($-6$ kcal days$^{-1}$; 95% CI: $-260$ to 248 kcal days$^{-1}$ vs. MODSUG, $p = 0.96$; Figure 2A). Furthermore, these inferences remained consistent when including change in body mass as a covariate (all $p > 0.35$), when assessing each intensity domain of physical activity, or when using a mixed model ($p = 0.32$ and $p = 0.71$ for LOWSUG and LOWCHO vs. MODSUG at week 4, and $p = 0.63$ and $p = 0.71$ for LOWSUG and LOWCHO vs. MODSUG at week 12) to account for dropouts.[21] Step count was also not meaningfully changed by condition (Table S4).

At week 4, there was no evidence of meaningful effects on resting metabolic rate (RMR) with either sugar restriction ($p = 0.72$; Table S4) or carbohydrate restriction ($p = 0.26$; Table S3). Similarly, at week 12, there was no evidence of meaningful effects on RMR with either sugar restriction ($p = 0.58$; Table S3) or carbohydrate restriction ($p = 0.76$). Furthermore, these inferences remained consistent when including change in fat-free mass (FFM) as a covariate (all $p > 0.47$; Table S3). Sleeping heart rate, fasting diastolic blood pressure, and fasting systolic blood pressure were not altered by condition (all $p \geq 0.06$; Table S4).

Both self-reported (Figure 2B) and researcher-measured (Figure 2C) body mass were reduced by sugar restriction and carbohydrate restriction. Compared to MODSUG, sugar restriction reduced body mass at week 4 ($p = 0.05$) and week 12 ($p = 0.04$). The reduction in body mass with sugar restriction was entirely explained by FM ($p < 0.001$; Figure 2D). Compared with MODSUG, carbohydrate restriction also reduced body mass at week 4 ($p < 0.001$) and week 12 ($p < 0.001$). The reduction in body mass with carbohydrate restriction at week 4 was explained by reductions in both FFM and FM (FFM: $p = 0.013$; FM: $p < 0.001$; Figure 2D), although this was not the case by week 12, where FM explained most of the body mass loss (FFM: $p = 0.281$; FM: $p < 0.001$; Figure 2D). Measures of body composition are also displayed in Table S5.

Self-reported energy intake is displayed in absolute units in Figure 2E and as change from baseline in Figure 2F. Compared with MODSUG, the sugar restriction lowered self-reported energy intake from baseline to week 4 ($p = 0.05$) without a detectable difference between LOWSUG and MODSUG across weeks 4–12 ($p = 0.32$). No detectable difference between carbohydrate restriction and MODSUG was observed for self-reported energy intake between weeks 0–4 ($p = 0.11$) or weeks 4–12 ($p = 0.69$). Over the full 12 weeks (baseline to 12 weeks analysis), the change in self-reported energy intake was $-19$ kcal•days$^{-1}$ (95% CI: $-250$ to 212 kcal•days$^{-1}$) in the MODSUG group, $-236$ kcal•days$^{-1}$ (95% CI: $-415$ to $-56$ kcal•days$^{-1}$) in the LOWSUG group ($p = 0.10$ vs. MODSUG), and $-172$ kcal•days$^{-1}$ (95% CI: $-338$ to $-6.6$ kcal•days$^{-1}$) in the LOWCHO group ($p = 0.24$ vs. MODSUG).

Sugar restriction reduced objectively calculated (via the intake-balance method[22]) energy intake between baseline and week 4 ($p = 0.02$) without a detectable difference between LOWSUG and MODSUG between weeks 4 and 12 ($p = 0.15$). Carbohydrate restriction reduced calculated energy intake from baseline to week 4 ($p < 0.001$) without a detectable difference between LOWCHO and MODSUG across weeks 4–12 ($p = 0.22$; Figure 2G). Across the entire 12 weeks (baseline to 12 weeks analysis), the change in objectively calculated energy intake was $-96$ kcal•days$^{-1}$ (95% CI: $-345$ to 153 kcal•days$^{-1}$) in the MODSUG group, $-352$ kcal•days$^{-1}$ (95% CI: $-560$ to $-145$ kcal•days$^{-1}$) in the LOWSUG group ($p = 0.19$ vs. MODSUG), and $-398$ kcal•days$^{-1}$ (95% CI: $-703$ to $-92$ kcal•days$^{-1}$) in the LOWCHO group ($p = 0.09$ vs. MODSUG).

### Ketogenic carbohydrate restriction reduces fasting and nocturnal glycemia but induces glucose intolerance and suppresses postprandial lactate responses

Fasting serum metabolic, endocrine, and hematological markers are presented in Table S6. At week 4, fasting glucose was lowered by carbohydrate restriction ($p = 0.004$), but not with sugar restriction ($p = 0.16$ vs. MODSUG). At week 12, fasting glucose was no longer significantly reduced by carbohydrate restriction versus MODSUG ($p = 0.14$). The difference in fasting glucose between sugar restriction and MODSUG at week 12 was $-0.4$ mmol•L$^{-1}$ (95% CI: 0.0 to $-0.9$ mmol•L$^{-1}$; $p = 0.06$). Carbohydrate restriction increased the postprandial glucose response to a mixed meal test at week 4 and week 12 with both the incremental area under the curve (iAUC) (Figures 3A and 3B) and peak glucose concentrations at week 4 ($p = 0.002$) but not week 12 ($p = 0.11$). In contrast, no meaningful differences were observed with sugar restriction vs. MODSUG in the postprandial glucose response to a mixed test meal (iAUC and peak glucose at weeks 4 and 12, all $p > 0.49$ vs. MODSUG; Figures 3A and 3B).

Compared to MODSUG, postprandial C-peptide was higher with carbohydrate restriction at week 4 ($p = 0.02$; Figure 3F), but there was no evidence of other meaningful effects of either sugar or carbohydrate restriction on fasting insulin or C-peptide concentrations, postprandial insulin or C-peptide iAUC, or peak insulin or C-peptide concentrations (all $p > 0.07$ vs. MODSUG; Figures 3C–3F). Postprandial peak lactate concentrations were lower with carbohydrate restriction at both week 4 ($p < 0.001$) and week 12 ($p = 0.003$), but not by sugar restriction (week 4: $p = 0.85$; week 12: $p = 0.43$; Figures 3G and 3H).

Carbohydrate restriction increased fasting beta-hydroxybutyrate (βOHB) concentrations at week 4 ($p < 0.001$) and week 12

bars) and fat free mass (FFM; shadowed bars, data stacked) at 4 weeks and 12 weeks (week 4 $n = 53$; week 12 $n = 45$; D). Self-reported energy intake (EI) across 12 weeks (E), change in self-reported EI across 4 weeks and 12 weeks (F), and change in objectively calculated EI across 4 weeks and 12 weeks using the intake-balance method (week 4 $n = 52$; week 12 $n = 43$; G). Data were all analyzed as ANCOVA-adjusted mean (95% CI) at week 4 and week 12 (with baseline scores as the covariate), apart from F and G which are unadjusted mean (95% CI) since values require calculation as the change from baseline. (a) $p \leq 0.05$ for a difference between LOWSUG vs. MODSUG at the time point where this letter appears; (b) $p \leq 0.05$ for a difference between LOWCHO vs. MODSUG at the time point where this letter appears; *$p \leq 0.05$ vs. MODSUG; **$p \leq 0.01$ vs. MODSUG; ***$p \leq 0.001$ vs. MODSUG.

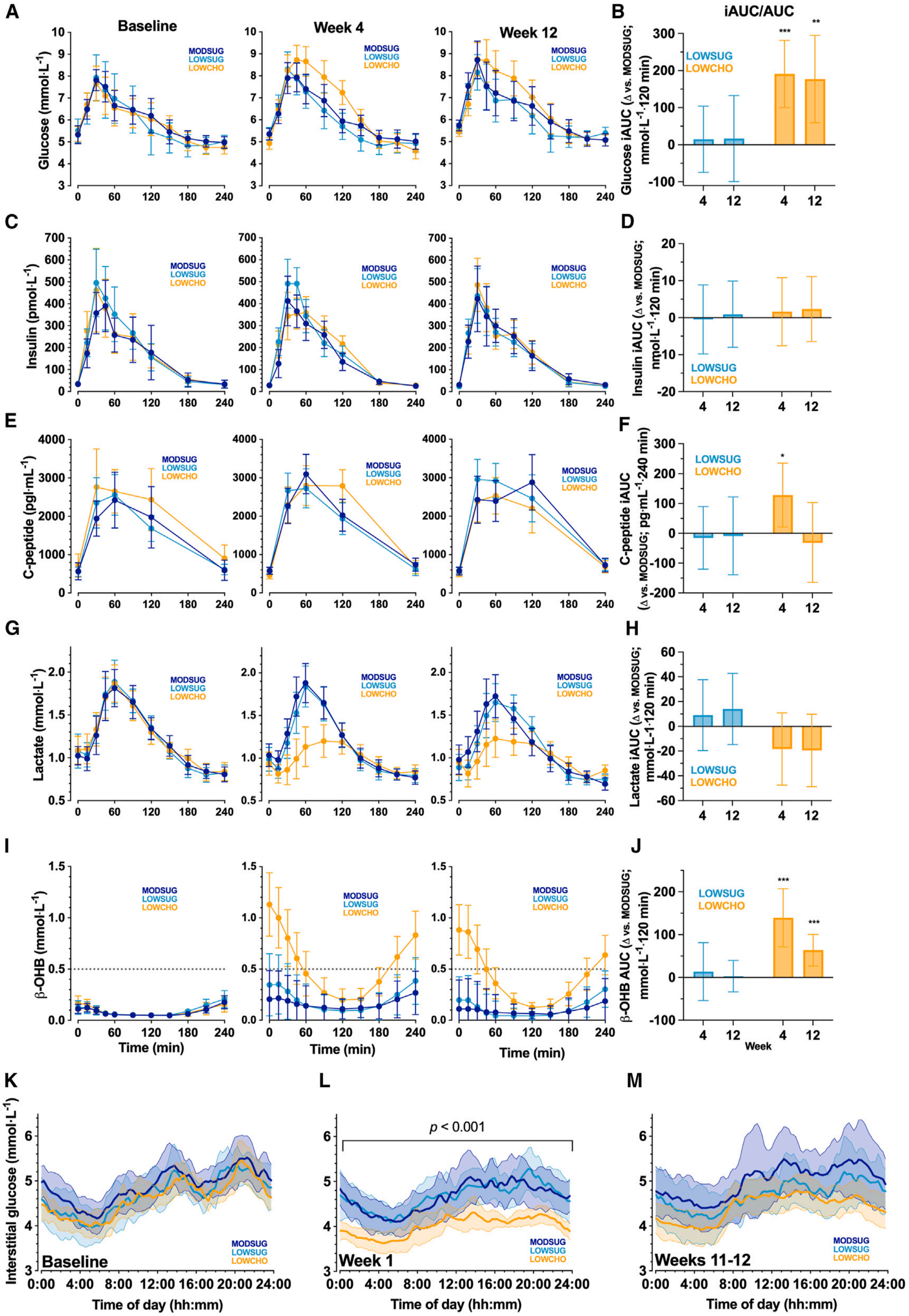


(legend on next page)

by ($p < 0.001$), whereas sugar restriction did not meaningfully alter fasting βOHB concentrations at either week 4 ($p = 0.59$) or week 12 ($p = 0.60$). Following ingestion of the mixed macronutrient test meal, postprandial βOHB concentrations were suppressed in all groups, but participants in the LOWCHO group re-entered ketosis within 4 h (Figure 3I). As such, βOHB total area under the curve (tAUC) was greater in LOWCHO compared to MODSUG at both week 4 and week 12 (both $p \leq 0.001$; Figure 3J).

Interstitial glucose and derived indices are displayed in Table S7 with baseline diurnal concentrations shown in Figure 3K. During week 1, carbohydrate restriction reduced mean daily interstitial glucose concentrations compared to MODSUG ($p < 0.001$; Figure 3L), but by weeks 11–12, a reduction was not detected ($p = 0.27$; Figure 3M).

## Weight loss from sugar restriction and a ketogenic diet elicits divergent systemic lipoprotein, amino acid, and lipid responses

Fasting plasma from baseline and week 4 was analyzed using NMR spectroscopy for metabolite and lipoprotein profiles, with the change from baseline to week 4 in each condition displayed in Figure 4A and Table S8. Most changes in NMR spectra were observed with carbohydrate restriction, whereby concentrations of most glucogenic amino acids decreased (e.g., alanine and glutamine) and of most branched-chain amino acids increased (e.g., isoleucine, leucine, and valine). Furthermore, in LOWCHO, there was an increase in particle concentration of all sizes of low-density lipoproteins (LDLs) and the lipid content in particles across a broad spectrum from medium VLDLs (very-low-density lipoproteins) to small LDLs, with some decrease in total lipid content of medium high-density lipoprotein (HDL) particles (all $p < 0.05$). Glycoprotein acetyls also decreased with both carbohydrate restriction and sugar restriction (Figure 4A; $p < 0.05$). Moreover, sugar restriction also demonstrated a modest reduction in the lipid content of small and medium HDL particles (both $p < 0.05$).

Sugar restriction did not detectably alter total cholesterol concentrations at week 4 ($p = 0.09$) but reduced total cholesterol concentrations at week 12 ($p = 0.01$; Figure 4B). Changes in total cholesterol with sugar restriction were almost entirely accounted for by reductions in LDL cholesterol (LDL-C) with sugar restriction at week 4 ($p = 0.02$) and week 12 ($p = 0.01$; Figure 4D). However, these decreases in LDL-C were not reflected by a meaningful difference in apolipoprotein B (apoB) concentrations at either week 4 or week 12 (both $p > 0.47$; Figure 4E). In contrast, compared with MODSUG, there was an increase in total cholesterol with carbohydrate restriction at week 4 ($p = 0.02$; Figure 4B), which was not completely reflected by LDL-C concentrations ($p = 0.10$; Figure 4D), but was concurrent with an increase in apoB concentrations ($p = 0.006$; Figure 4E). By week 12, there was no evidence of meaningful differences in total cholesterol, HDL cholesterol, LDL-C, or apoB concentrations with carbohydrate restriction vs. MODSUG (all $p > 0.16$).

There was little evidence of any meaningful effects of sugar restriction on fasting triacylglycerol (TAG) concentrations vs. MODSUG ($p > 0.76$ for both week 4 and 12), but carbohydrate restriction increased fasting TAG concentrations at both week 4 and week 12 (both $p = 0.04$ vs. MODSUG; Figure 4E). Compared to MODSUG, postprandial peak TAG concentrations were not different with sugar or carbohydrate restriction at week 4 or week 12 (all $p > 0.12$), but postprandial TAG iAUC was decreased by carbohydrate restriction at week 12 ($p = 0.02$; Figure 4F).

No evidence was observed for meaningful effects of sugar restriction on fasting or postprandial glycerol or non-esterified fatty acid (NEFA) concentrations (all $p > 0.50$ vs. MODSUG; Figures 4G–4J). Carbohydrate restriction, on the other hand, increased fasting concentrations and postprandial area under the curve (AUC) for glycerol and NEFAs at week 4 (all $p \leq 0.02$ vs. MODSUG; Figures 4H and 4J). By week 12, however, the only increase that clearly remained was for the postprandial NEFA AUC ($p = 0.01$ vs. MODSUG; Figure 4J).

## The ketogenic diet, but not sugar restriction, altered gut microbial diversity without functional changes in gut permeability markers or SCFA concentrations

While there was little evidence of broad changes in the fecal microbiome with sugar restriction (Figures 5A–5C), carbohydrate restriction altered beta diversity (assessed by nonmetric multidimensional scaling; NMDS) of fecal species at week 12 ($p = 0.04$; Figure 5C). There were, however, no large changes observed in alpha diversity with either sugar or carbohydrate restriction (Figure 5D). No significant differences between groups were observed at the phylum or species levels at either week 4 or week 12 (Figure S3). At the genus level, however, (Figure S4), ketogenic carbohydrate restriction lowered the abundance of *Bifidobacterium* by week 4 (Figure 5G; $q = 0.04$), which was sustained until week 12 (Figure 5H; $q = 0.01$), by which time ketogenic carbohydrate restriction had also lowered the abundance of *Planococcus* ($q = 0.03$). The reduction in *Bifidobacterium* was largely explained by reductions in the species *adolescentis* with contributions from other species (Figure S5).

To understand the potential impact of changes in beta diversity and relative abundance, we explored the HUMANn2 normalized unstratified metabolic pathway abundances with NMDS. No significant differences were observed between groups in NMDS of metabolic pathway abundances with minimal separation

**Figure 3. Glucose, insulin, C-peptide, lactate, and beta-hydroxybutyrate responses to mixed meal tolerance tests (mean [range] 502 [331 to 715 kcal]; 54% carbohydrate [23% of which are sugars], 31% fat, 15% protein), and continuous interstitial glucose concentrations at baseline and across 12 weeks of MODSUG, LOWSUG, or LOWCHO diets**

(A–M) Unadjusted postprandial concentrations of serum glucose (A), insulin (C) C-peptide (E), lactate (G), and β-hydroxybutyrate (I; βOHB), and ANCOVA-adjusted mean (95% CI, baseline as the covariate) incremental/total area under the curve (tAUC) expressed as differences vs. MODSUG (B, D, F, H, and J, respectively; week 4 $n = 48$; week 12 $n = 42$). Twenty-four-hour interstitial glucose concentrations at baseline (K), week 1 (L), and weeks 11–12 (M); data are means (95% CIs) for (K), and ANCOVA-adjusted means (95% CIs) for (L) and (M); week 1 $n = 38$; weeks 11–12 $n = 32$. *$p \leq 0.05$ vs. MODSUG; **$p \leq 0.01$ vs. MODSUG; ***$p \leq 0.001$ vs. MODSUG.

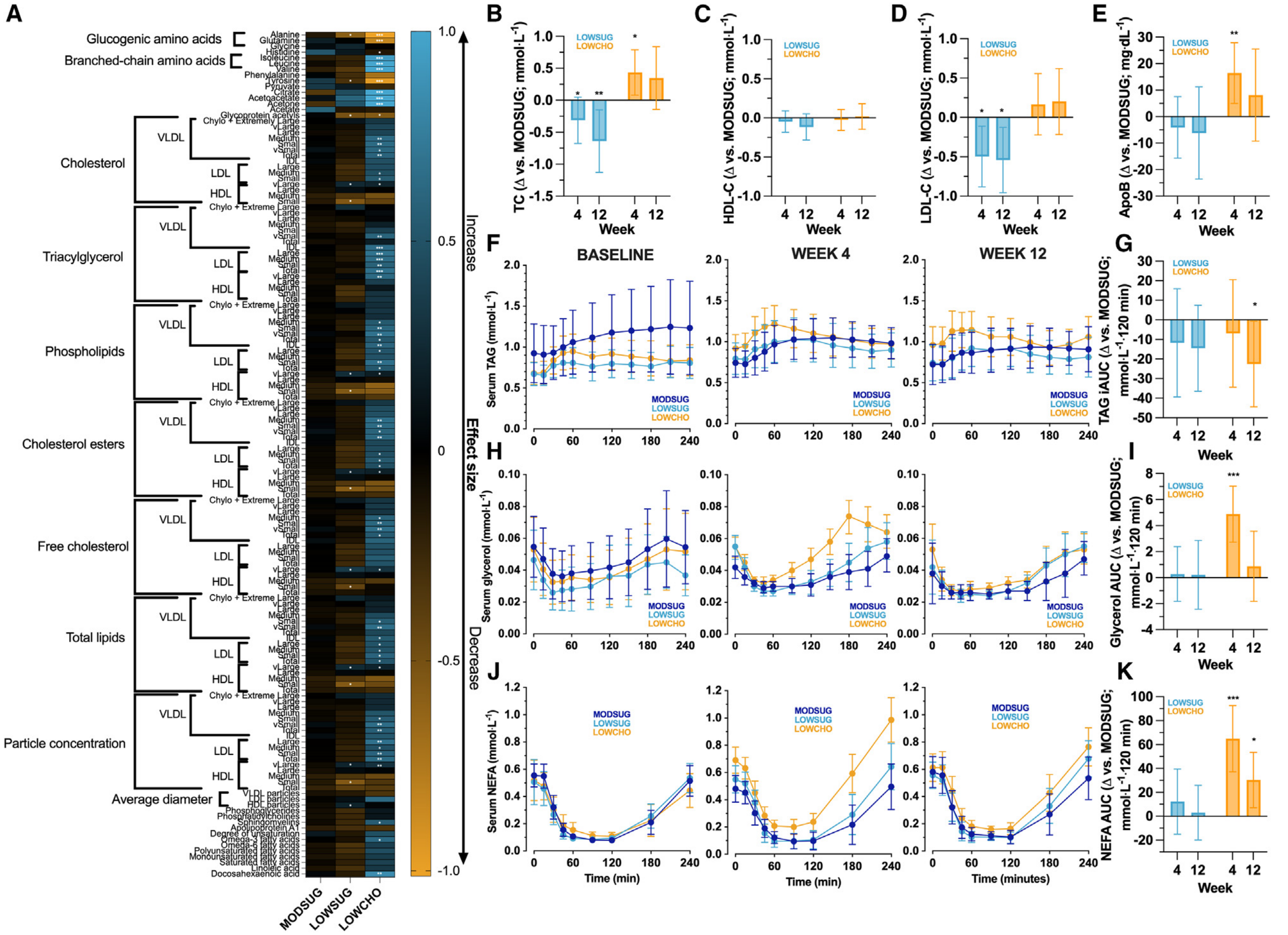


**Figure 4. Targeted nuclear magnetic resonance spectroscopy of fasting plasma lipoprotein and metabolite profiles, fasting serum cholesterol and apolipoprotein B concentrations, and triacylglycerol, glycerol, and non-esterified fatty acid responses to mixed meal tolerance tests (mean [range] 502 [331 to 715 kcal]; 54% carbohydrate [23% of which are sugars], 31% fat, 15% protein) after 4 or 12 weeks of MODSUG, LOWSUG, or LOWCHO diets**

(A–J) Change in nuclear magnetic resonance (NMR)-derived fasting plasma lipoprotein and metabolite profile from baseline to week 4 (A), expressed as effect size ($n$ = 49). *$p \leq 0.05$, **$p \leq 0.01$, ***$p \leq 0.001$ week 4 vs. baseline (e.g., blue with an asterisk represents a significant increase from baseline and yellow indicates a decrease from baseline); ANCOVA-adjusted mean (95% CI, baseline as the covariate) differences vs. MODSUG in fasting serum total cholesterol (B; TC) high-density lipoprotein cholesterol (C; HDL-C); low-density lipoprotein cholesterol (D; LDL-C), and apolipoprotein B (E; apoB) concentrations; unadjusted post-prandial concentrations of serum triacylglycerol (F; TAG), glycerol (H), and non-esterified fatty acid (J; NEFA) and ANCOVA-adjusted mean (95% CI, baseline as the co-variate) incremental/tAUC differences vs. MODSUG (G, I, and K, respectively; week 4 $n$ = 48; week 12 $n$ = 42). *$p \leq 0.05$ vs. MODSUG; **$p \leq 0.01$ vs. MODSUG; ***$p \leq 0.001$ vs. MODSUG in B–J.

($q$ > 0.1). While no significant differences were observed at any time point between LOWSUG and MODSUG (all $q$ > 0.10), LOWCHO altered the beta diversity of metabolic pathway abundances at both week 4 ($q$ = 0.02 vs. MODSUG) and week 12 ($q$ = 0.04 vs. MODSUG). There were no significant differences between groups at baseline in any pathway abundances (all $q$ > 0.1); however, at week 4 and week 12 there was some evidence of differential pathway abundance with LOWCHO vs. MODSUG, whereby several biosynthetic pathways (e.g., methionine, sulfate, cysteine, and palmitate) displayed positive effect sizes while several degradation pathways displayed negative effect sizes (e.g., purine ribonucleosides, galactose, and glycolysis; Figure S6).

We also measured the serum concentrations of SCFAs and lipopolysaccharide-binding protein (LBP). At week 4, compared to MODSUG, serum levels of the SCFA propionate were reduced with carbohydrate restriction ($p$ = 0.03; Figure 5J), but there was no evidence of any other effects of either sugar or carbohydrate restriction on fasting plasma acetate, propionate, butyrate, or LBP concentrations at week 4 or week 12 (all $p$ > 0.26; Figures 5I–5L).

## Carbohydrate restriction altered skeletal muscle and adipose tissue metabolic phenotype

To further understand the adipose and skeletal muscle adaptations in response to the diets, we interrogated the short-term (4-week) transcriptional responses of 19 key metabolic genes

CellPress
OPEN ACCESS

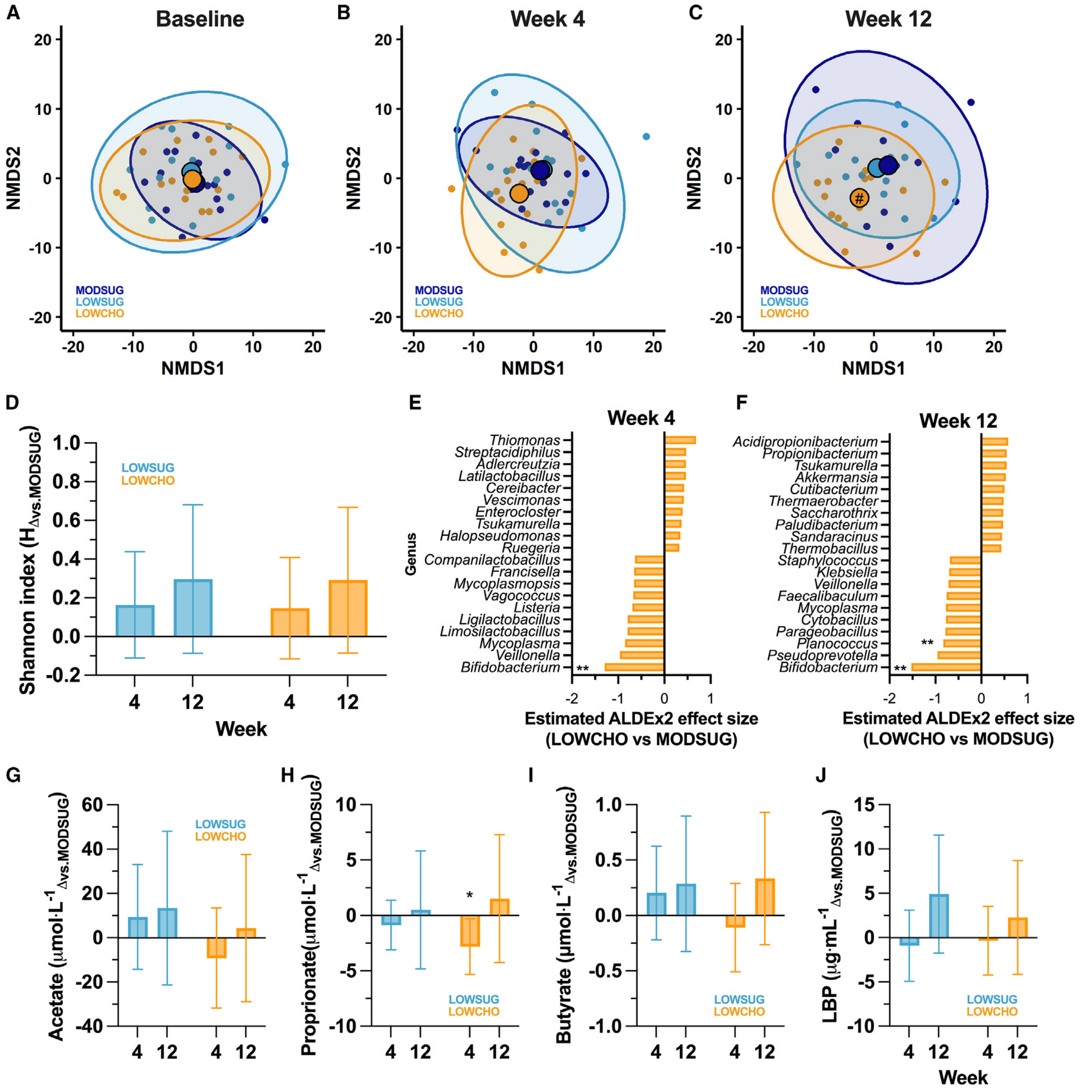


**Figure 5. Beta diversity, alpha diversity, and major genus-level changes in gut microbiome composition, and concentrations of plasma short-chain fatty acids and lipopolysaccharide-binding protein across 12 weeks of MODSUG, LOWSUG, or LOWCHO diets**

(A–J) Nonmetric multidimensional scaling (NMDS) plots from robust Aitchison distances of all species at baseline, week 4, and week 12 (A, B, and C). Large points indicate centroids. Centroid containing # indicates LOWCHO vs. MODSUG $q = 0.10$. Alpha diversity as expressed as the Shannon index with ANOVA-adjusted means ± 95% CI (D, baseline as the covariate). Estimated effect size of change in center log ratio abundance of the top 20 gut microbiome genera with the largest differences between LOWSUG and MODSUG at week 4 (E) and week 12 (F), and between LOWCHO and MODSUG at week 4 (G) and week 12 (H), *$q < 0.1$; **$q < 0.05$. Plasma concentrations of acetate (G), propionate (H), butyrate (I), and lipopolysaccharide-binding protein (LBP; J). Data are means (95% CIs) at baseline, and ANCOVA-adjusted means (95% CIs) during the interventions (with baseline scores as the covariate). Week 4 $n = 48$, week 12 $n = 41$ for microbiome outcomes. Week 4 $n$ = 26–50, week 12 $n$ = 18–43 for circulating factors. *$p \leq 0.05$ vs. MODSUG; **$p \leq 0.01$ vs. MODSUG; ***$p \leq 0.001$.

in adipose tissue by quantitative reverse-transcription PCR (Table S9) and the short-term (4-week) and longer-term (12-week) responses of 12 key metabolic proteins in skeletal muscle by western blotting. Carbohydrate restriction increased adipose tissue lipoprotein lipase (*LPL*) mRNA content ($p = 0.04$) and decreased adipose tissue *Adiponectin* mRNA content ($p = 0.03$)

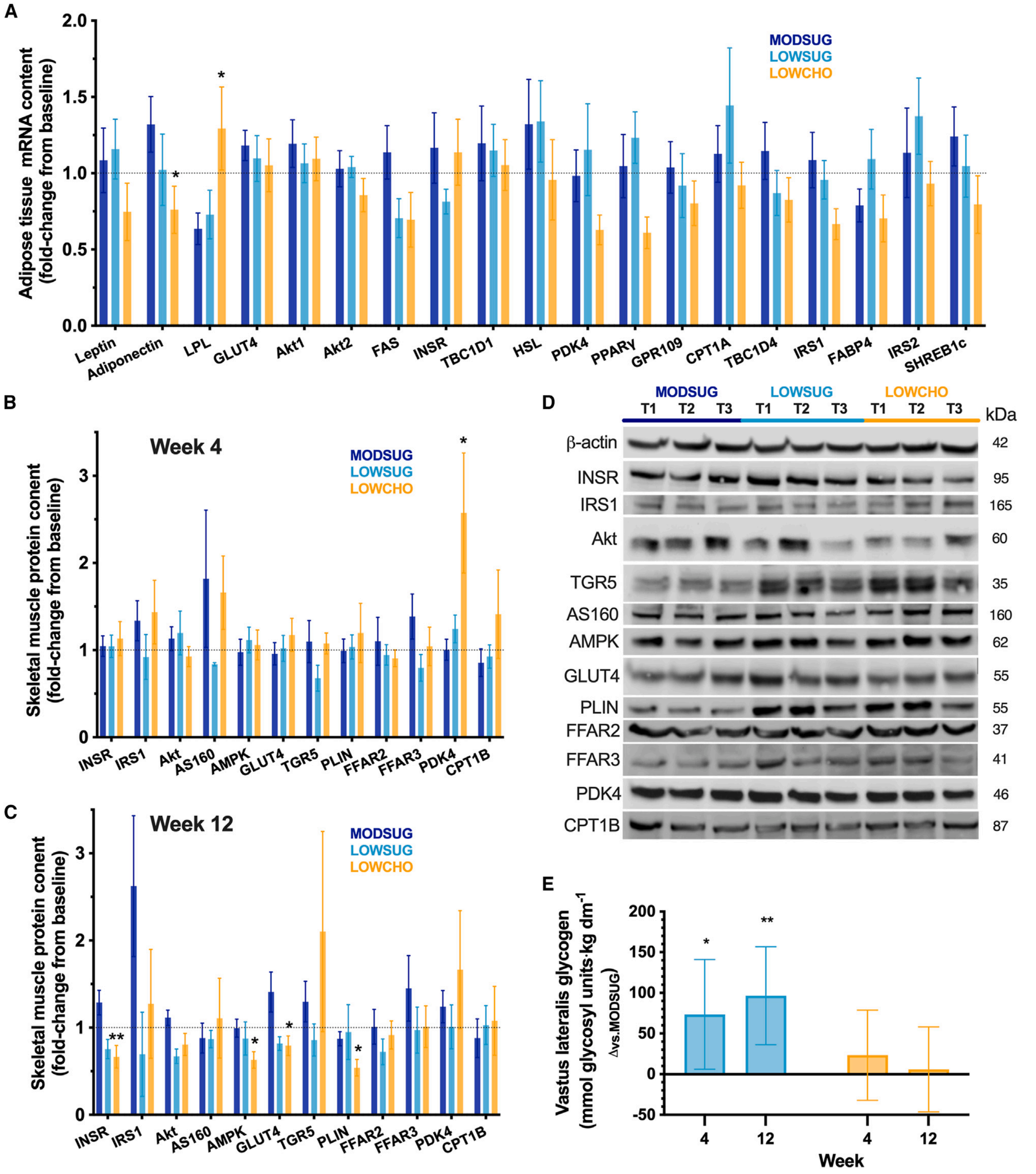


**Figure 6. Adipose tissue mRNA content and skeletal muscle protein and glycogen content across 4 and 12 weeks of MODSUG, LOWSUG, or LOWCHO diets**

(A–D) Fold changes in levels of mRNA in adipose tissue at week 4 ($n$ = 38; A) and key proteins in skeletal muscle at week 4 ($n$ = 27; B) and week 12 ($n$ = 19; C), with representative blots (D). Data for (A)–(C) are mean (SEM).

(E) ANCOVA-adjusted mean (95% CI) difference vs. MODSUG in skeletal muscle glycogen concentrations (week 4 $n$ = 29; week 12 $n$ = 21). *$p \leq 0.05$ vs. MODSUG; **$p \leq 0.01$ vs. MODSUG; ***$p \leq 0.001$ vs. MODSUG.

at week 4 compared with MODSUG, without detectable differences in a variety of other genes involved in insulin signaling, glucose, and lipid metabolism (Figure 6A). Carbohydrate restriction also increased skeletal muscle pyruvate dehydrogenase kinase 4 (PDK4) protein levels at week 4 ($p = 0.04$ vs. MODSUG; Figure 6B) and decreased insulin receptor (INSR), adenosine monophosphate-activated protein kinase (AMPK), glucose transporter 4 (GLUT4), and perilipin 1 (PLIN) at week 12 ($p = 0.006$, $p = 0.02$, $p = 0.03$, and $p = 0.02$ vs. MODSUG, respectively; Figure 6C). Representative blots are displayed in Figure 6D. Sugar restriction resulted in fewer changes with the only detectable change being decreased Akt (protein kinase B) at week 12 ($p = 0.02$ vs. MODSUG), while also increasing glycogen concentrations at week 4 ($p = 0.03$) and week 12 ($p = 0.004$; Figure 6E). In contrast, ketogenic carbohydrate restriction did not meaningfully alter muscle glycogen concentrations at either week 4 ($p = 0.40$) or week 12 ($p = 0.82$; Figure 6E).

### Integrated endocrine response, food preference, and appetite

Compared to MODSUG, sugar restriction lowered circulating fasting leptin concentrations at week 12 ($p = 0.006$; Figure 7A) but did not meaningfully impact fasting or postprandial GLP-1, FGF21, ghrelin, or C-reactive protein (CRP) concentrations (all $p > 0.09$ vs. MODSUG; Figures 7B–7H). In contrast, while carbohydrate restriction also reduced fasting leptin concentrations at week 4 and week 12 (both $p \leq 0.002$ vs. MODSUG; Figure 7A), reductions in fasting and postprandial FGF21 were also observed with carbohydrate restriction at week 12 (both $p \leq 0.01$ vs. MODSUG; Figures 7C and 7G), alongside an increase in fasting, but not postprandial, circulating GLP-1 concentrations (Figures 7B and 7F). Time-course data for postprandial GLP-1, FGF21, and ghrelin are displayed in Figure S2.

No differences were detected using the food preference task test battery in either fasting or postprandial preferences for high-carbohydrate sweet foods, high-carbohydrate savory foods, or low-carbohydrate savory foods at week 4 or week 12 (all $p > 0.05$; Figure 7I). However, both sugar restriction and carbohydrate restriction were associated with increased visual analog scale scores for the desire for sweet foods at week 12 compared with baseline (Figure 7J).

## DISCUSSION

This experiment demonstrates that restricting free sugar below 5% energy intake or restricting overall carbohydrate to below 8% energy intake does not substantially change free-living PAEE in adults without obesity. This suggests that changes in physical activity observed previously with breakfast skipping[16] and alternate day fasting[17] may be due to complete absence of energy intake, rather than lack of carbohydrate per se. Preservation of physical activity (and total) energy expenditure was seen despite energy intake being free-to-vary by design. Consequently, observed reductions in body (and fat) mass with free-sugar and carbohydrate restriction were explained by reduced energy intake. These data demonstrate that adherence to guidelines (e.g., UK and World Health Organization[1,3]) restricting free-sugar intake to 5% energy intake reduces objectively measured energy intake by over 400 kcal$\cdot$days$^{-1}$ (versus MODSUG) for 1 month, and reductions are sustained with smaller magnitude over 3 months. While both sugar restriction and the ketogenic diet induced an energy deficit via reduced energy intake, the most substantial changes in whole-body and tissue-specific metabolism and gut microbial composition were only seen with ketogenic carbohydrate restriction.

Although free-living nutrition studies can overcome some of the limitations (e.g., external validity) seen with domiciled studies, they often lack adherence to interventions, and self-report diet measures can limit measurement validity of energy intake. To address these issues, we employed several objective compliance markers and calculated energy intake via an objective method (the intake-balance method[22]). Importantly, while the direction of effect on energy intake was similar with both self-report and objective methods, the magnitude and sensitivity to detect effects of sugar and carbohydrate restriction on changes in energy intake were improved by the objective method over self-report. Causal evidence supporting guidelines to restrict free (or added)-sugar intakes is almost entirely based on self-report methods of energy intake, with only one study using researcher-weighed energy intake over 1 week.[23] Meta-regression of randomized controlled trials (RCTs) suggests that each 1% energy reduction from free sugars results in a reduction of self-reported energy intake by $\sim$14 kcal$\cdot$days$^{-1}$.[1] The data in the current study align with this, demonstrating a reduction in free-sugar intake of $\sim$15% reduces self-reported energy intake of $\sim$217 kcal$\cdot$days$^{-1}$ (i.e., 14 kcal/day per %energy) over 12 weeks compared with control. Importantly, however, our objective assessment of energy intake suggests that self-report methods underestimate the relationship between free-sugar intakes and total energy intake. This may be partly due to reporting fatigue, and thus increasing error in the self-report measures of energy intake. Objective assessment of energy intake demonstrated a 256 kcal$\cdot$days$^{-1}$ reduction in energy intake with free-sugar restriction versus control over 12 weeks (equivalent to 17 kcal$\cdot$days$^{-1}$ per % energy). The true dose response of free-sugar restriction on energy intake may therefore be larger than the 14 kcal$\cdot$days$^{-1}$ per 1% energy intake currently proposed. This may have implications for the population targets for free-(or added)-sugar intakes.

In the absence of changes in energy expenditure (and assuming energy balance at baseline), reductions in energy intake would be expected to reduce body (and fat) mass and improve overall cardiometabolic health. Indeed, 12 weeks of sugar restriction reduced fasting glucose and LDL-C concentrations by 0.4 mmol$\cdot$L$^{-1}$ (95% CI: 0 to 0.9 mmol$\cdot$L$^{-1}$) and 0.5 mmol$\cdot$L$^{-1}$ (95% CI: 0.1 to 1.0 mmol$\cdot$L$^{-1}$) vs. MODSUG, respectively. Elevated fasting glucose and LDL-C concentrations have been associated with mortality even within a "healthy" range.[24,25] Reduced fasting glucose with sugar restriction and energy deficit could be explained by increased hepatic and/or peripheral (skeletal muscle and adipose tissue) insulin sensitivity. The lack of effect of sugar restriction on postprandial glucose and insulin concentrations, and of adipose tissue mRNA or skeletal muscle proteins involved in insulin sensitivity, is consistent with an increase in hepatic insulin sensitivity primarily explaining reduced fasting glucose concentrations with sugar

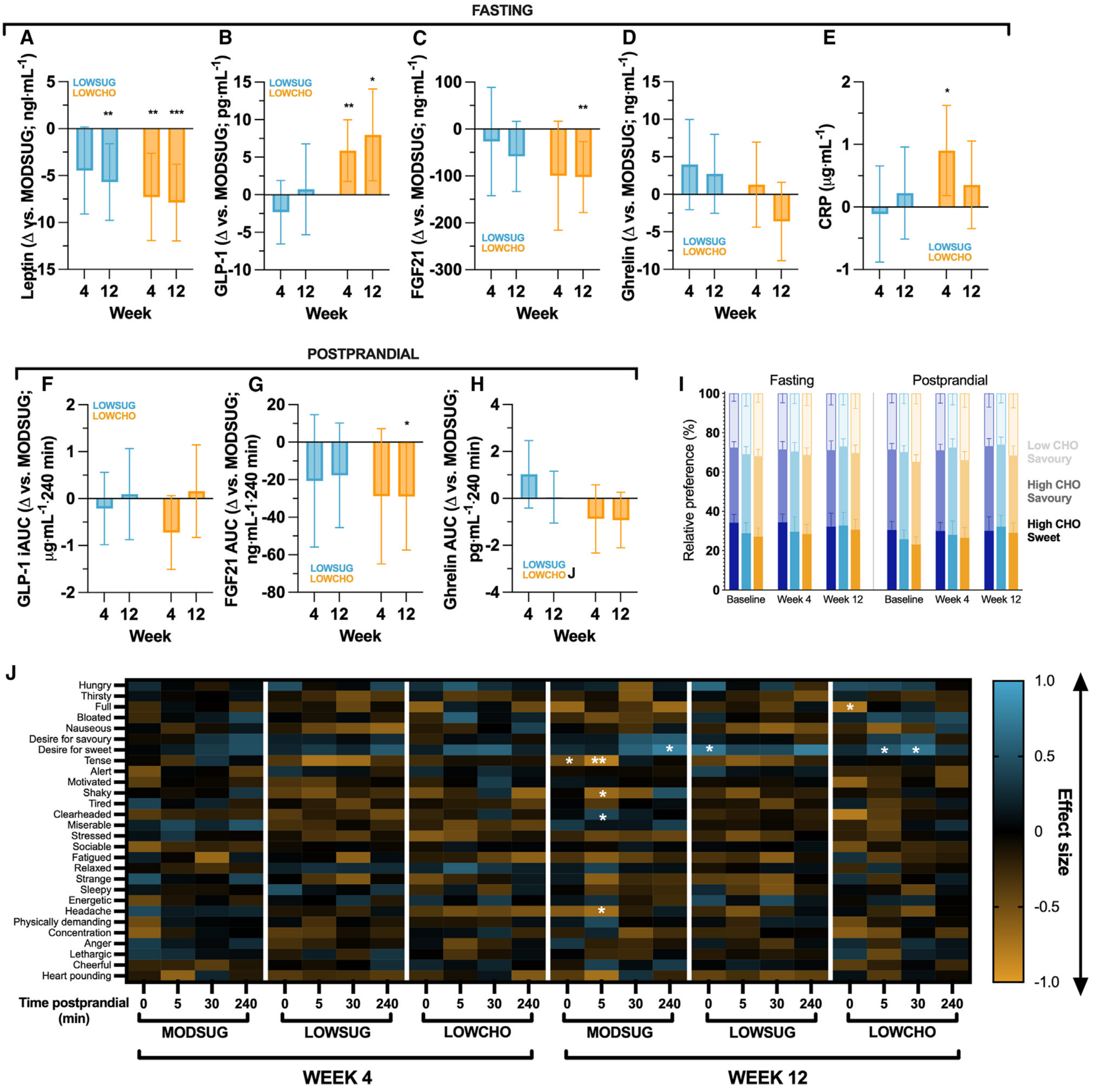


**Figure 7. Fasting and postprandial endocrine and subjective behavioral responses across 4 and 12 weeks of MODSUG, LOWSUG, or LOWCHO diets**

(A–J) Fasting leptin (A), glucagon-like peptide 1 (GLP-1; B), fibroblast growth factor 21 (FGF21; C), ghrelin (D), and C-reactive protein (CRP; E). Postprandial GLP-1 iAUC (F), FGF21 tAUC (G), and ghrelin tAUC (H) differences vs. MODSUG. Relative preference for high-carbohydrate sweet foods, high-carbohydrate savory foods, and low-carbohydrate savory foods (I). Visual analog scales. Data in A–H are ANCOVA-adjusted mean (95% CI) differences vs. MODSUG. Data in I are mean (95% CI) at baseline, and ANCOVA-adjusted mean (95% CI) for week 4 and 12 (with baseline scores as the covariate). Data in J are mean effect size of change between baseline and week 4 or week 12 (*$p < 0.05$ vs. baseline). *$p \leq 0.05$ vs. MODSUG; **$p \leq 0.01$ vs. MODSUG; ***$p \leq 0.001$ for A–H. Week 4 $n = 41$–$50$; week 12 $n = 36$–$43$.

restriction. Further evidence for favorable cardiometabolic effects of sugar restriction includes reduced glycoprotein acetyls (GlycA). The GlycA signal from NMR represents the integrated concentration and glycosylation of several acute phase proteins and is a novel marker of systemic inflammation, suggested to be a better predictor of cardiovascular disease (CVD) risk than CRP.[26,27] While lower LDL-C would generally be expected to associate with reduced CVD risk, the number of apoB containing

CellPress
OPEN ACCESS

lipoproteins is thought to be the causal factor linking lipoproteins to atherosclerotic CVD.[28] We found little evidence that sugar restriction meaningfully altered the number of apoB-containing lipoproteins based on either apoB concentrations or NMR spectroscopy of VLDL, intermediate-density lipoprotein, or LDL particle concentrations. This suggests that the reduction in LDL-C concentration with sugar restriction is not explained by a reduced number of LDL particles but rather by reduced cholesterol enrichment of LDL particles, likely because of the energy deficit achieved by sugar restriction.

In contrast to the cardiometabolic effects of weight loss from sugar restriction, ketogenic carbohydrate restriction resulted in a wide range of divergent cardiometabolic responses. Ketogenic carbohydrate restriction reduced daily mean glycemia and glycemic variability within week 1 and fasting glucose concentrations by ~0.5 mmol$\cdot$L$^{-1}$ (95% CI: −0.2 to −0.8 mmol$\cdot$L$^{-1}$) at week 4. However, these effects were not preserved at week 12 despite ongoing ketosis. In addition to a potential improvement in hepatic insulin sensitivity at week 4, the reduction in fasting glucose concentrations could be a direct effect of βOHB, which can suppress endogenous glucose production and reduce glycemia.[9,29] Upon reintroduction of carbohydrates during a mixed meal tolerance test, postprandial glycemia was exaggerated with the ketogenic diet, coinciding with reduced postprandial rise in lactate concentrations. In the postprandial state, skeletal muscle disposes of a substantial proportion of circulating glucose via oxidation, storage as glycogen, and/or interconversion (e.g., into lactate[30]). Increased postprandial glycemia and reduced lactate therefore likely reflect reduced peripheral insulin sensitivity, glucose uptake, and glycolysis. Furthermore, whereas the energy deficit from sugar restriction resulted in an increase in muscle glycogen concentrations at week 12, no such effect was seen with ketogenic carbohydrate restriction. Within skeletal muscle, the regulation of glucose uptake and glycolysis is coordinated by a variety of proteins with roles in insulin signaling, transarcolemmal transport, and intracellular metabolism. The ketogenic diet increased skeletal muscle PDK4 levels (at week 4) and reduced AMPK, GLUT4, and insulin receptor levels (at week 12). PDK4 increases under conditions of high fatty acid availability/oxidation and downregulates pyruvate dehydrogenase complex activity,[31] a rate limiting step in glucose metabolism.[32] Therefore, increased skeletal muscle PDK4 is consistent with a metabolic shift away from glycolysis, which could be due to increased fatty acid availability and/or increased branched-chain amino acid availability. GLUT4 is the insulin-sensitive glucose transporter in skeletal muscle that plays a role in insulin-stimulated skeletal muscle glucose uptake; changes in skeletal muscle GLUT4 content have relevance to insulin sensitivity and postprandial glycemia.[33] As such, the reduced skeletal muscle GLUT4 content (alongside reduced insulin receptor and AMPK) provides an additional mechanistic explanation for the reduced glucose tolerance with adaptation to a ketogenic diet and is consistent with reduced capacity for insulin-stimulated (postprandial) skeletal muscle glucose uptake.

In addition to reducing glucose tolerance, the ketogenic diet increased apoB concentrations by ~16 mg$\cdot$dL$^{-1}$ (95% CI: 5 to 28 mg$\cdot$dL$^{-1}$) and fasting triacylglycerol concentrations at week 4. Consistent with this were increases in particle concentration of all sizes of LDLs and of small and very small VLDLs. While total LDL-C concentrations were not increased versus MODSUG, carbohydrate restriction did increase cholesterol concentrations in medium and small LDL particles. These changes suggest that, despite an energy deficit, the ketogenic diet can increase the number of circulating atherogenic lipoproteins in the short term. Interestingly, the differences in fasting apoB concentrations between groups were not apparent at week 12. Alongside the loss of short-term effects on fasting and daily glycemia, the lack of meaningful differences between carbohydrate restriction and control at week 12 may reflect adaptation to the ketogenic diet and/or differences in energy balance status (weight loss in the first 4 weeks versus weight maintenance in the last 4 weeks). A time-dependent effect was also apparent for CRP, whereby carbohydrate restriction increased circulating CRP concentrations vs. control at week 4, but differences in CRP between groups were not meaningful by week 12. CRP is almost entirely produced by the liver and is tightly linked to hepatic fatty acid status.[34,35] During the first 4 weeks, carbohydrate restriction also produced an energy deficit, with consequent increases in fasting and postprandial glycerol and NEFA concentrations from lipolysis. By week 12, energy balance was re-established as demonstrated by stable body mass, and consequently, fasting/postprandial glycerol concentrations were no longer meaningfully different from control. The higher lipolysis and fatty acid availability from combined carbohydrate/calorie restriction and increased fat intake during the first 4 weeks may therefore explain the transient increase in CRP concentrations, as the liver handles excess lipids. Adipose tissue *LPL* mRNA was also increased with carbohydrate restriction at week 4, which may be an adaptive response to clear excess circulating triglycerides. By week 12, energy balance is re-established and therefore some of the lipolytic stimulus and thus hepatic lipid availability is diminished. It is notable that CRP and GlycA displayed divergent responses in the first 4 weeks and may suggest GlycA is more responsive to energy balance status whereas CRP may be more responsive to hepatic fatty acid availability.

Ketogenic carbohydrate restriction altered gut microbial beta diversity at the levels of both taxonomic composition and functional potential after 12 weeks, with particularly noteworthy significant reductions in the relative abundance of *Bifidobacteria* by week 4 and persisting at week 12. This is causal evidence that longer-term (12-week) ketogenic diets change the gut microbial composition in healthy adults.[36,37] Dietary fiber supports survival and activity of intestinal microorganisms such as *Bifidobacteria*,[38] and fiber intake was reduced to ~15 g per day (reduction of ~40% vs. MODSUG) with ketogenic carbohydrate restriction. *Bifidobacteria* are typically considered “favorable” gut microorganisms and, indeed, are commonly consumed as probiotics.[39] Supplementation with *Bifidobacterium lactis* lowers LDL-C concentrations,[40] and therefore, the reductions in *Bifidobacteria* with a ketogenic diet may contribute to the observed changes in lipoprotein profiles. We found no evidence of meaningful changes in circulating SCFA or LBP concentrations in the fasted state with ketogenic carbohydrate restriction (other than a small reduction in propionate at week 4) and no change in the basal expression of G protein-coupled receptors in skeletal muscle activated by SCFAs (FFAR2 and FFAR3). We cannot establish whether ketogenic carbohydrate restriction altered hepatic and splanchnic

uptake or appearance of SCFAs, although this is a possibility.[41] Thus, at the present time, while the present RCT provides robust evidence that carbohydrate restriction changes gut microbial populations after 12 weeks, it is not yet possible to establish the biological significance of these changes.

Under free-living conditions, the reduction in energy intake induced by either free sugar or total carbohydrate restriction can be due to a combination of physiological, social, and behavioral factors. Carbohydrate-restrictive diets, including low-sugar, tend to be lower in palatability, which reduce energy intake.[42] Since the ketogenic diet is even more restrictive with food choice (and variety) than sugar-restricted diets, this could (partly) explain the large initial weight loss with carbohydrate restriction. Over time, physiological feedback can be expected to increase appetite to counteract fat loss.[43] Consistent with this, we observed reduced leptin concentrations with both sugar and carbohydrate restriction and reduced fasting FGF21 with the ketogenic diet. This reduction in FGF21 with a ketogenic diet is consistent with other human data and conflicts rodent data.[44,45] Accordingly, the reduction in energy intake with both diets was clearest (and largest) in the first 4 weeks, with weight loss beginning to plateau thereafter, when reductions in leptin and FGF21 were most apparent. Furthermore, ratings of appetite showed little differences at week 4, but by week 12, there was evidence of increased desire for sweet food following either sugar or total carbohydrate restriction in the fasted/early-to-mid postprandial state. This is consistent with evidence that FGF21 and leptin influence sugar intake and sweet taste sensitivity, respectively,[46,47] and suggests that hormonally driven appetite feedback to energy deficits induced by sugar and carbohydrate restriction can take more than 4 weeks to manifest in increased appetite.

## Limitations of the study

The objective (intake-balance) method of assessing energy intake relies on accurate estimation of changes in body energy stores. Dual-energy X-ray absorptiometry (DXA) was used to assess changes in body energy stores from FM and FFM. Changes in FFM measured by DXA during energy deficit and carbohydrate restriction may be influenced by fluid shifts and potentially liver glycogen.[48,49] However, these would influence shorter-term assessments (i.e., within days), and uncertainty of the DXA-based intake-balance method is minimized after 21 days[22] Therefore, over the time frames measured in the current study, the assumptions associated with the DXA-based intake-balance method are reasonable. The intake-balance method was originally developed using doubly labeled water for energy expenditure.[22] However, since accurate measurement of energy expenditure using doubly labeled water requires knowledge of the RER during the measurement period, this can be challenging under free-living conditions with low-carbohydrate diets.[50] Our data further highlight this issue with fasting and postprandial RER not matching food quotient (FQ) during ketosis. This is most likely due to increased ketone body production/oxidation, supported by observations that exercise RER did match FQ during ketosis, when skeletal muscle predominates whole-body RER. We therefore used accelerometry with branched-equation modeling, which has been used to assess free-living PAEE under a variety of conditions,[16,19,51,52] and add this to direct measures of resting metabolic rate. Importantly, we individually calibrated these devices to account for changes in the heart rate-energy expenditure relationship over time, and this method does not rely on RER for accuracy. Participants were not informed that physical activity was the primary outcome, nor was physical activity or body weight emphasized in meetings. However, prior beliefs about diets in relation to physical activity and body weight could contribute to observed responses, which reflect free-living scenarios. The control condition in the current study consisted of free-sugar intakes that approximated the 70$^{th}$ percentile of reported UK intakes[53] and thus could be considered high relative to the UK population. However, the free-sugar intakes in the control group were relatively similar to the habitual free-sugar intakes of the sample in the current study. The relatively large difference in sugar intake this creates between control and sugar-restricted groups means that an absence of effect on the primary outcome makes it possible to rule out the likelihood that smaller changes in sugar restriction would meaningfully alter energy expenditure. Finally, while we had multiple biomarkers to confirm adherence to the ketogenic diet, we lacked a biomarker for adherence to sugar restriction. Nevertheless, the fact that weight loss was seen in the group advised to restrict intake of sugars demonstrates that an energy-containing dietary nutrient must have been restricted, and sustained RER under fasted, postprandial, and exercising conditions confirmed that total carbohydrate intake (and FQ) was maintained, suggesting that sugar restriction was achieved. Some biomarkers exist for intakes of added sugars in the US, such as carbon isotope ratio of breath.[54] However, since sugar sources in Europe contain less natural abundance of $^{13}C$ (due to inclusion of beet and other sugar sources), the validity of these biomarkers for sugar restriction in Europe is unclear.[55] Due to dropouts and COVID-19 mitigation, the final sample size was smaller than the target, and thus the statistical power for the primary outcome could be diminished. However, based on the *a priori* power calculation, the final sample size should still provide >80% power for week 4 analyses and >71% power for week 12 analyses. Furthermore, the mean difference, even when using per-protocol analysis that can inflate effect sizes, is not of a biologically meaningful magnitude. Accordingly, it is unlikely that the intervention diets meaningfully alter PAEE. Other exploratory outcomes should be interpreted with more caution.

## Conclusions

In summary, despite inducing an energy-deficit and subsequent physiological feedback on appetite with dietary restriction of either free sugars or overall carbohydrates, we found no evidence of feedback on energy expenditure, including PAEE. Dietary free-sugar restriction reduced FM and LDL-C concentrations compared to control (MODSUG), but this was not accompanied by meaningful changes in postprandial metabolism. While ketogenic carbohydrate restriction also reduced body mass and FM compared to control, this was accompanied by more varied and divergent metabolic effects. Ketogenic carbohydrate restriction increased fat oxidation, transiently reduced both fasting and interstitial glucose concentrations, and transiently increased apoB and postprandial triglyceride concentrations. After 12 weeks of ketogenic carbohydrate restriction, there were substantial changes

to the fecal microbial profile, insulin and energy signaling, and glucose transport protein levels in skeletal muscle, and circulating GLP-1, FGF21, and triglycerides. These findings suggest that substantial fat loss can be achieved by restricting either free sugars or a ketogenic diet despite differences in how these diets affect whole-body and peripheral tissue metabolism. Whereas free-sugar restriction had modest effects on whole-body or tissue-specific metabolism or the gut microbiome, ketogenic carbohydrate restriction had wide-ranging effects on metabolism, with sustained increases in whole-body and skeletal muscle fat oxidation, reductions in glucose tolerance, and alterations in gut microbial beta diversity. These data suggest that ketogenic carbohydrate restriction may not necessarily produce a cardiometabolic health benefit that would be expected by the weight loss observed. Instead, free-sugar restriction may be a more appropriate dietary choice for overall cardiometabolic health for many people. Neither type of dietary restriction appears to meaningfully alter PAEE.

## STAR★METHODS

Detailed methods are provided in the online version of this paper and include the following:

- KEY RESOURCES TABLE
- RESOURCE AVAILABILITY
  - Lead contact
  - Materials availability
  - Data and code availability
- EXPERIMENTAL MODEL AND STUDY PARTICIPANT DETAILS
- METHOD DETAILS
  - Study protocol
  - Preliminary measures
  - Randomization
  - Laboratory visits
  - Design of the diets
  - Implementation and adherence
  - Physical activity
  - Anthropometry and body composition
  - Indirect calorimetry and substrate oxidation
  - Dietary assessment and energy intake
  - DIT and food quotient
  - Food preference, appetite, and mood
  - Submaximal exercise test
  - Blood pressure and heart rate
  - Continuous glucose monitoring
  - Adipose tissue biopsies
  - Skeletal muscle biopsies
  - Blood sample processing and analyses
  - Urine sample processing and analyses
  - Fecal sample collection
  - Microbiome analyses
  - Short-chain fatty acid measurement
  - Muscle glycogen
  - Muscle western blotting
  - Adipose tissue RNA extraction
  - Adipose tissue mRNA content by qPCR
- QUANTIFICATION AND STATISTICAL ANALYSES
- ADDITIONAL RESOURCES

## SUPPLEMENTAL INFORMATION

Supplemental information can be found online at https://doi.org/10.1016/j.xcrm.2024.101667.

## ACKNOWLEDGMENTS

This work was supported by an initial grant from the Rank Prize Funds with subsequent funding from the Cosun Nutrition Center, the University of Bath, and Ian Tarr. Nuclear magentic resonance spectroscopy was funded by BHF Senior fellowships (FS/15/56/31645 and FS/SBSRF/21/31013) to L.H. F.K. is funded by the MRC (MR/P0002927/1). The authors thank the participants for their time and commitment to the study.

## AUTHOR CONTRIBUTIONS

Conceptualization, A.H., R.G.D., L.J.C.v.L., F.K., J.A.B., D.T., and J.T.G.; methodology, A.H., R.G.D., P.J.R., J.M.B., L.J.C.v.L., F.K., J.A.B., J.-P.W., D.T., and J.T.G.; formal analysis, A.H., R.G.D., J.B., L.H., W.B., F.C., O.O., P.D.C., F.K., J.-P.W., and J.T.G.; investigation, A.H., R.G.D., L.M., L.R., L.B., J.B., J.A.B., J.-P.W., and J.T.G.; data curation, A.H., R.G.D., and J.T.G.; writing – original draft, A.H., R.G.D., and J.T.G.; writing – review and editing, all co-authors; visualization, A.H., R.G.D., and J.T.G.; supervision, L.J.C.v.L., F.K., J.A.B., J.-P.W., D.T., and J.T.G.; funding acquisition, L.H., L.J.C.v.L., F.K., J.A.B., D.T., and J.T.G.

## DECLARATION OF INTERESTS

P.D.C. is a co-founder and CTO of SeqBiome Ltd. J.T.G. has received research funding from BBSRC, MRC, Cancer Research UK, the British Heart Foundation, Clasado Biosciences, Lucozade Ribena Suntory, Arla Foods Ingredients, the Cosun Nutrition Center, and the Fruit Juice Science Center; is a scientific advisory board member to ZOE; and has completed paid consultancy for 6d Sports Nutrition, The Dairy Council, PepsiCo, Violicom Medical, Tour Racing Ltd., and SVGC. J.A.B. is an investigator on research grants funded by BBSRC, MRC, the British Heart Foundation, Rare Disease Foundation, EU Hydration Institute, GlaxoSmithKline, Nestlé, Lucozade Ribena Suntory, Arla Foods, Cosun Nutrition Center, American Academy of Sleep Medicine Foundation, and Salus Optima (L3M Technologies Ltd.); has completed paid consultancy for PepsiCo, Kellogg's, SVGC, and Salus Optima (L3M Technologies Ltd.); is Company Director of Metabolic Solutions Ltd.; receives an annual honorarium as a member of the academic advisory board for the International Olympic Committee Diploma in Sports Nutrition; and receives an annual stipend as Editor-in-Chief of International Journal of Sport Nutrition & Exercise Metabolism.

Received: February 2, 2024
Revised: April 1, 2024
Accepted: July 9, 2024
Published: August 5, 2024

## REFERENCES

1. Scientific Advisory Committee on Nutrition (2015). Carbohydrates and health report (Public Health England).
2. Swan, G.E., Powell, N.A., Knowles, B.L., Bush, M.T., and Levy, L.B. (2018). A definition of free sugars for the UK. Publ. Health Nutr. *21*, 1636–1638. https://doi.org/10.1017/S136898001800085X.
3. World Health Organization (2015). Guideline: sugars intake for adults and children. https://www.who.int/publications/i/item/9789241549028.
4. Scientific Advisory Committee on Nutrition (2015). Why 5%? An explanation of SACN's recommendations about sugars and health. https://www.gov.uk/government/publications/sacns-sugars-and-health-recommendations-why-5.
5. Te Morenga, L., Mallard, S., and Mann, J. (2013). Dietary sugars and body weight: systematic review and meta-analyses of randomised controlled trials and cohort studies. BMJ Br. Med. J. (Clin. Res. Ed.) *346*, e7492. https://doi.org/10.1136/bmj.e7492.
6. Erickson, J., Sadeghirad, B., Lytvyn, L., Slavin, J., and Johnston, B.C. (2017). The Scientific Basis of Guideline Recommendations on Sugar

Intake: A Systematic Review. Ann. Intern. Med. *166*, 257–267. https://doi.org/10.7326/M16-2020.

7. Hengist, A., Davies, R.G., Rogers, P.J., Brunstrom, J.M., van Loon, L.J.C., Walhin, J.P., Thompson, D., Koumanov, F., Betts, J.A., and Gonzalez, J.T. (2022). Restricting sugar or carbohydrate intake does not impact physical activity level or energy intake over 24 h despite changes in substrate use: a randomised crossover study in healthy men and women. Eur. J. Nutr. *62*, 921–940. https://doi.org/10.1007/s00394-022-03048-x.
8. Hall, K.D., and Guo, J. (2017). Obesity Energetics: Body Weight Regulation and the Effects of Diet Composition. Gastroenterology *152*, 1718–1727.e3. https://doi.org/10.1053/j.gastro.2017.01.052.
9. Mikkelsen, K.H., Seifert, T., Secher, N.H., Grondal, T., and van Hall, G. (2015). Systemic, cerebral and skeletal muscle ketone body and energy metabolism during acute hyper-D-beta-hydroxybutyratemia in post-absorptive healthy males. J. Clin. Endocrinol. Metab. *100*, 636–643. https://doi.org/10.1210/jc.2014-2608.
10. Moller, N. (2020). Ketone Body, 3-Hydroxybutyrate: Minor Metabolite - Major Medical Manifestations. J. Clin. Endocrinol. Metab. *105*, 2884–2892. https://doi.org/10.1210/clinem/dgaa370.
11. Scientific Advisory Committee on Nutrition (2021). Lower carbohydrate diets for adults with type 2 diabetes. https://www.gov.uk/government/publications/sacn-report-lower-carbohydrate-diets-for-type-2-diabetes.
12. Smith, H.A., Gonzalez, J.T., Thompson, D., and Betts, J.A. (2017). Dietary carbohydrates, components of energy balance, and associated health outcomes. Nutr. Rev. *75*, 783–797. https://doi.org/10.1093/nutrit/nux045.
13. Bandini, L.G., Schoeller, D.A., and Dietz, W.H. (1994). Metabolic differences in response to a high-fat vs. a high-carbohydrate diet. Obes. Res. *2*, 348–354. https://doi.org/10.1002/j.1550-8528.1994.tb00074.x.
14. Jensen, T., Abdelmalek, M.F., Sullivan, S., Nadeau, K.J., Green, M., Roncal, C., Nakagawa, T., Kuwabara, M., Sato, Y., Kang, D.H., et al. (2018). Fructose and sugar: A major mediator of non-alcoholic fatty liver disease. J. Hepatol. *68*, 1063–1075. https://doi.org/10.1016/j.jhep.2018.01.019.
15. Postic, C. (2020). Conversion of a dietary fructose: new clues from the gut microbiome. Nat. Metab. *2*, 217–218. https://doi.org/10.1038/s42255-020-0185-x.
16. Betts, J.A., Richardson, J.D., Chowdhury, E.A., Holman, G.D., Tsintzas, K., and Thompson, D. (2014). The causal role of breakfast in energy balance and health: a randomized controlled trial in lean adults. Am. J. Clin. Nutr. *100*, 539–547. https://doi.org/10.3945/ajcn.114.083402.
17. Templeman, I., Smith, H.A., Chowdhury, E., Chen, Y.C., Carroll, H., Johnson-Bonson, D., Hengist, A., Smith, R., Creighton, J., Clayton, D., et al. (2021). A randomized controlled trial to isolate the effects of fasting and energy restriction on weight loss and metabolic health in lean adults. Sci. Transl. Med. *13*, eabd8034. https://doi.org/10.1126/scitranslmed.abd8034.
18. Brage, S., Brage, N., Franks, P.W., Ekelund, U., Wong, M.-Y., Andersen, L.B., Froberg, K., and Wareham, N.J. (2004). Branched equation modeling of simultaneous accelerometry and heart rate monitoring improves estimate of directly measured physical activity energy expenditure. J. Appl. Physiol. *96*, 343–351.
19. Thompson, D., Batterham, A.M., Bock, S., Robson, C., and Stokes, K. (2006). Assessment of low-to-moderate intensity physical activity thermogenesis in young adults using synchronized heart rate and accelerometry with branched-equation modeling. J. Nutr. *136*, 1037–1042.
20. Gladden, L.B., Yates, J.W., and Howley, E.T. (2012). Who needs a bag? Med. Sci. Sports Exerc. *44*, 288–289.
21. Gadbury, G., Coffey, C., and Allison, D. (2003). Modern statistical methods for handling missing repeated measurements in obesity trial data: beyond LOCF. Obes. Rev. *4*, 175–184.
22. Ravelli, M.N., and Schoeller, D.A. (2021). An objective measure of energy intake using the principle of energy balance. Int. J. Obes. *45*, 725–732.
23. Mazlan, N., Horgan, G., Whybrow, S., and Stubbs, J. (2006). Effects of increasing increments of fat- and sugar-rich snacks in the diet on energy and macronutrient intake in lean and overweight men. Br. J. Nutr. *96*, 596–606.
24. Emerging Risk Factors Collaboration (2011). Diabetes mellitus, fasting glucose, and risk of cause-specific death. N. Engl. J. Med. *364*, 829–841.
25. Ference, B.A., Ginsberg, H.N., Graham, I., Ray, K.K., Packard, C.J., Bruckert, E., Hegele, R.A., Krauss, R.M., Raal, F.J., Schunkert, H., et al. (2017). Low-density lipoproteins cause atherosclerotic cardiovascular disease. 1. Evidence from genetic, epidemiologic, and clinical studies. A consensus statement from the European Atherosclerosis Society Consensus Panel. Eur. Heart J. *38*, 2459–2472. https://doi.org/10.1093/eurheartj/ehx144.
26. Chiesa, S.T., Charakida, M., Georgiopoulos, G., Roberts, J.D., Stafford, S.J., Park, C., Mykkanen, J., Kahonen, M., Lehtimaki, T., Ala-Korpela, M., et al. (2022). Glycoprotein Acetyls: A Novel Inflammatory Biomarker of Early Cardiovascular Risk in the Young. J. Am. Heart Assoc. *11*, e024380. https://doi.org/10.1161/JAHA.121.024380.
27. Chiesa, S.T., Charakida, M., Georgiopoulos, G., Roberts, J.D., Stafford, S.J., Park, C., Mykkänen, J., Kähönen, M., Lehtimäki, T., and Ala-Korpela, M. (2022). Glycoprotein acetyls: a novel inflammatory biomarker of early cardiovascular risk in the young. J. Am. Heart Assoc. *11*, e024380.
28. Glavinovic, T., Thanassoulis, G., de Graaf, J., Couture, P., Hegele, R.A., and Sniderman, A.D. (2022). Physiological Bases for the Superiority of Apolipoprotein B Over Low-Density Lipoprotein Cholesterol and Non-High-Density Lipoprotein Cholesterol as a Marker of Cardiovascular Risk. J. Am. Heart Assoc. *11*, e025858. https://doi.org/10.1161/JAHA.122.025858.
29. Evans, E., Walhin, J.-P., Hengist, A., Betts, J.A., Dearlove, D.J., and Gonzalez, J.T. (2022). Ketone monoester ingestion increases post-exercise serum erythropoietin concentrations in healthy men. Am. J. Physiol. Endocrinol. Metabol. *324*, E56–E61.
30. Kelley, D., Mitrakou, A., Marsh, H., Schwenk, F., Benn, J., Sonnenberg, G., Arcangeli, M., Aoki, T., Sorensen, J., and Berger, M. (1988). Skeletal muscle glycolysis, oxidation, and storage of an oral glucose load. J. Clin. Invest. *81*, 1563–1571.
31. Lundsgaard, A.M., Sjoberg, K.A., Hoeg, L.D., Jeppesen, J., Jordy, A.B., Serup, A.K., Fritzen, A.M., Pilegaard, H., Myrmel, L.S., Madsen, L., et al. (2017). Opposite Regulation of Insulin Sensitivity by Dietary Lipid Versus Carbohydrate Excess. Diabetes *66*, 2583–2595. https://doi.org/10.2337/db17-0046.
32. Chokkalingam, K., Jewell, K., Norton, L., Littlewood, J., Van Loon, L., Mansell, P., Macdonald, I., and Tsintzas, K. (2007). High-fat/low-carbohydrate diet reduces insulin-stimulated carbohydrate oxidation but stimulates nonoxidative glucose disposal in humans: An important role for skeletal muscle pyruvate dehydrogenase kinase 4. J. Clin. Endocrinol. Metab. *92*, 284–292.
33. Edinburgh, R.M., Koumanov, F., and Gonzalez, J.T. (2022). Impact of pre-exercise feeding status on metabolic adaptations to endurance-type exercise training. J. Physiol. *600*, 1327–1338. https://doi.org/10.1113/JP280748.
34. Foroughi, M., Maghsoudi, Z., Khayyatzadeh, S., Ghiasvand, R., Askari, G., and Iraj, B. (2016). Relationship between non-alcoholic fatty liver disease and inflammation in patients with non-alcoholic fatty liver. Adv. Biomed. Res. *5*, 28. https://doi.org/10.4103/2277-9175.176368.
35. Yeniova, A.O., Kucukazman, M., Ata, N., Dal, K., Kefeli, A., Basyigit, S., Aktas, B., Agladioglu, K., Akin, K.O., Ertugrul, D.T., et al. (2014). High-sensitivity C-reactive protein is a strong predictor of non-alcoholic fatty liver disease. Hepato-Gastroenterology *61*, 422–425.
36. Nakamura, K., Hagihara, K., Nagai, N., Egashira, R., Takeuchi, M., Nakano, M., Saito, H., Moriguchi, M., Tonari, S., Watanabe, S., et al. (2022). Ketogenic effects of multiple doses of a medium chain triglycerides enriched ketogenic formula in healthy men under the ketogenic

CellPress
OPEN ACCESS

diet: A randomized, double-blinded, placebo-controlled study. Nutrients *14*, 1199.

37. Rew, L., Harris, M.D., and Goldie, J. (2022). The ketogenic diet: Its impact on human gut microbiota and potential consequent health outcomes: A systematic literature review. Gastroenterol. Hepatol. Bed Bench *15*, 326–342.
38. Lai, H., Li, Y., He, Y., Chen, F., Mi, B., Li, J., Xie, J., Ma, G., Yang, J., Xu, K., et al. (2023). Effects of dietary fibers or probiotics on functional constipation symptoms and roles of gut microbiota: a double-blinded randomized placebo trial. Gut Microb. *15*, 2197837. https://doi.org/10.1080/19490976.2023.2197837.
39. Wieers, G., Belkhir, L., Enaud, R., Leclercq, S., Philippart de Foy, J.M., Dequenne, I., de Timary, P., and Cani, P.D. (2019). How Probiotics Affect the Microbiota. Front. Cell. Infect. Microbiol. *9*, 454. https://doi.org/10.3389/fcimb.2019.00454.
40. Bernini, L.J., Simão, A.N.C., Alfieri, D.F., Lozovoy, M.A.B., Mari, N.L., de Souza, C.H.B., Dichi, I., and Costa, G.N. (2016). Beneficial effects of Bifidobacterium lactis on lipid profile and cytokines in patients with metabolic syndrome: A randomized trial. Effects of probiotics on metabolic syndrome. Nutrition *32*, 716–719.
41. Bloemen, J.G., Venema, K., van de Poll, M.C., Olde Damink, S.W., Buurman, W.A., and Dejong, C.H. (2009). Short chain fatty acids exchange across the gut and liver in humans measured at surgery. Clin. Nutr. *28*, 657–661. https://doi.org/10.1016/j.clnu.2009.05.011.
42. Rogers, P.J., Vural, Y., Berridge-Burley, N., Butcher, C., Cawley, E., Gao, Z., Sutcliffe, A., Tinker, L., Zeng, X., Flynn, A.N., et al. (2024). Evidence that carbohydrate-to-fat ratio and taste, but not energy density or NOVA level of processing, are determinants of food liking and food reward. Appetite *193*, 107124.
43. Rogers, P.J., and Brunstrom, J.M. (2016). Appetite and energy balancing. Physiol. Behav. *164*, 465–471.
44. Song, P., Zechner, C., Hernandez, G., Cánovas, J., Xie, Y., Sondhi, V., Wagner, M., Stadlbauer, V., Horvath, A., Leber, B., et al. (2018). The hormone FGF21 stimulates water drinking in response to ketogenic diet and alcohol. Cell Metabol. *27*, 1338–1347.e4.
45. Rosenbaum, M., Hall, K.D., Guo, J., Ravussin, E., Mayer, L.S., Reitman, M.L., Smith, S.R., Walsh, B.T., and Leibel, R.L. (2019). Glucose and lipid homeostasis and inflammation in humans following an isocaloric ketogenic diet. Obesity *27*, 971–981.
46. Niki, M., Jyotaki, M., Yoshida, R., Yasumatsu, K., Shigemura, N., DiPatrizio, N.V., Piomelli, D., and Ninomiya, Y. (2015). Modulation of sweet taste sensitivities by endogenous leptin and endocannabinoids in mice. J. Physiol. *593*, 2527–2545.
47. von Holstein-Rathlou, S., BonDurant, L.D., Peltekian, L., Naber, M.C., Yin, T.C., Claflin, K.E., Urizar, A.I., Madsen, A.N., Ratner, C., Holst, B., et al. (2016). FGF21 Mediates Endocrine Control of Simple Sugar Intake and Sweet Taste Preference by the Liver. Cell Metabol. *23*, 335–343. https://doi.org/10.1016/j.cmet.2015.12.003.
48. Luke, A., and Schoeller, D.A. (1992). Basal metabolic rate, fat-free mass, and body cell mass during energy restriction. Metabolism *41*, 450–456. https://doi.org/10.1016/0026-0495(92)90083-m.
49. Gonzalez, J.T., Batterham, A.M., Atkinson, G., and Thompson, D. (2023). Perspective: Is the response of human energy expenditure to increased physical activity additive or constrained? Adv. Nutr. *14*, 406–419.
50. Hall, K.D., Guo, J., Chen, K.Y., Leibel, R.L., Reitman, M.L., Rosenbaum, M., Smith, S.R., and Ravussin, E. (2019). Methodologic considerations for measuring energy expenditure differences between diets varying in carbohydrate using the doubly labeled water method. Am. J. Clin. Nutr. *109*, 1328–1334.
51. Edinburgh, R.M., Bradley, H.E., Abdullah, N.F., Robinson, S.L., Chrzanowski-Smith, O.J., Walhin, J.P., Joanisse, S., Manolopoulos, K.N., Philp, A., Hengist, A., et al. (2020). Lipid Metabolism Links Nutrient-Exercise Timing to Insulin Sensitivity in Men Classified as Overweight or Obese. J. Clin. Endocrinol. Metab. *105*, 660–676. https://doi.org/10.1210/clinem/dgz104.
52. Turner, J.E., Markovitch, D., Betts, J.A., and Thompson, D. (2010). Non-prescribed physical activity energy expenditure is maintained with structured exercise and implicates a compensatory increase in energy intake. Am. J. Clin. Nutr. *92*, 1009–1016.
53. Bates, B., Collins, D., Jones, K., Page, P., Roberts, C., Steer, T., and Swan, G. (2020). NDNS: results from years 9 to 11 (2016 to 2017 and 2018 to 2019). https://www.gov.uk/government/statistics/ndns-results-from-years-9-to-11-2016-to-2017-and-2018-to-2019.
54. O'Brien, D.M., Niles, K.R., Black, J., and Schoeller, D.A. (2021). The breath carbon isotope ratio reflects short-term added-sugar intake in a dose-response, crossover feeding study of 12 healthy adults. J. Nutr. *151*, 628–635.
55. O'Brien, D.M. (2015). Stable isotope ratios as biomarkers of diet for health research. Annu. Rev. Nutr. *35*, 565–594.
56. Ricciuto, L., Fulgoni, V.L., 3rd, Gaine, P.C., Scott, M.O., and DiFrancesco, L. (2021). Sources of Added Sugars Intake Among the U.S. Population: Analysis by Selected Sociodemographic Factors Using the National Health and Nutrition Examination Survey 2011-18. Front. Nutr. *8*, 687643. https://doi.org/10.3389/fnut.2021.687643.
57. Roberts, C., Steer, T., Maplethorpe, N., Cox, L., Meadows, S., Nicholson, S., Page, P., and Swan, G. (2018). National Diet and Nutrition Survey: Results from Years 7 and 8 (combined) of the Rolling Programme (2014/2015 to 2015/2016).
58. Wittekind, A., and Walton, J. (2014). Worldwide trends in dietary sugars intake. Nutr. Res. Rev. *27*, 330–345. https://doi.org/10.1017/S0954422414000237.
59. U.S. Department of Agriculture (2015). 2015–2020 Dietary Guidelines for Americans. https://www.dietaryguidelines.gov/sites/default/files/2019-05/2015-2020_Dietary_Guidelines.pdf.
60. Feinman, R.D., Pogozelski, W.K., Astrup, A., Bernstein, R.K., Fine, E.J., Westman, E.C., Accurso, A., Frassetto, L., Gower, B.A., McFarlane, S.I., et al. (2015). Dietary carbohydrate restriction as the first approach in diabetes management: critical review and evidence base. Nutrition *31*, 1–13. https://doi.org/10.1016/j.nut.2014.06.011.
61. Volek, J.S., and Phinney, S.D. (2012). The Art and Science of Low Carbohydrate Performance (Beyond Obesity LLC).
62. Brage, S., Ekelund, U., Brage, N., Hennings, M.A., Froberg, K., Franks, P.W., and Wareham, N.J. (2007). Hierarchy of individual calibration levels for heart rate and accelerometry to measure physical activity. J. Appl. Physiol. *103*, 682–692. https://doi.org/10.1152/japplphysiol.00092.2006.
63. Haskell, W.L., Lee, I.M., Pate, R.R., Powell, K.E., Blair, S.N., Franklin, B.A., Macera, C.A., Heath, G.W., Thompson, P.D., Bauman, A., et al. (2007). Physical activity and public health: updated recommendation for adults from the American College of Sports Medicine and the American Heart Association. Circulation *116*, 1081–1093. https://doi.org/10.1161/CIRCULATIONAHA.107.185649.
64. Tremblay, M.S., Aubert, S., Barnes, J.D., Saunders, T.J., Carson, V., Latimer-Cheung, A.E., Chastin, S.F.M., Altenburg, T.M., and Chinapaw, M.J.M.; Participants, S.T.C.P (2017). Sedentary Behavior Research Network (SBRN) - Terminology Consensus Project process and outcome. Int. J. Behav. Nutr. Phys. Activ. *14*, 75. https://doi.org/10.1186/s12966-017-0525-8.
65. Haugen, H.A., Chan, L.N., and Li, F. (2007). Indirect calorimetry: a practical guide for clinicians. Nutr. Clin. Pract. *22*, 377–388. https://doi.org/10.1177/0115426507022004377.
66. Betts, J.A., and Thompson, D. (2012). Thinking outside the bag (not necessarily outside the lab). Med. Sci. Sports Exerc. *44*, 2040. https://doi.org/10.1249/MSS.0b013e318264526f.
67. Frayn, K.N. (1983). Calculation of substrate oxidation rates in vivo from gaseous exchange. J. Appl. Physiol. Respir. Environ. Exerc. Physiol. *55*, 628–634.

68. Jeukendrup, A.E., and Wallis, G.A. (2005). Measurement of substrate oxidation during exercise by means of gas exchange measurements. Int. J. Sports Med. *26*, S28–S37. https://doi.org/10.1055/s-2004-830512.

69. Elia, M., and Cummings, J.H. (2007). Physiological aspects of energy metabolism and gastrointestinal effects of carbohydrates. Eur. J. Clin. Nutr. *61*, S40–S74. https://doi.org/10.1038/sj.ejcn.1602938.

70. Westerterp, K.R. (2004). Diet induced thermogenesis. Nutr. Metab. *1*, 5. https://doi.org/10.1186/1743-7075-1-5.

71. Jéquier, E., Acheson, K., and Schutz, Y. (1987). Assessment of energy expenditure and fuel utilization in man. Annu. Rev. Nutr. *7*, 187–208. https://doi.org/10.1146/annurev.nu.07.070187.001155.

72. Borg, G. (1970). Perceived exertion as an indicator of somatic stress. Scand. J. Rehabil. Med. *2*, 92–98.

73. Battelino, T., Danne, T., Bergenstal, R.M., Amiel, S.A., Beck, R., Biester, T., Bosi, E., Buckingham, B.A., Cefalu, W.T., Close, K.L., et al. (2019). Clinical Targets for Continuous Glucose Monitoring Data Interpretation: Recommendations From the International Consensus on Time in Range. Diabetes Care *42*, 1593–1603. https://doi.org/10.2337/dci19-0028.

74. Bergenstal, R.M., Beck, R.W., Close, K.L., Grunberger, G., Sacks, D.B., Kowalski, A., Brown, A.S., Heinemann, L., Aleppo, G., Ryan, D.B., et al. (2018). Glucose Management Indicator (GMI): A New Term for Estimating A1C From Continuous Glucose Monitoring. Diabetes Care *41*, 2275–2280. https://doi.org/10.2337/dc18-1581.

75. Danne, T., Nimri, R., Battelino, T., Bergenstal, R.M., Close, K.L., DeVries, J.H., Garg, S., Heinemann, L., Hirsch, I., Amiel, S.A., et al. (2017). International Consensus on Use of Continuous Glucose Monitoring. Diabetes Care *40*, 1631–1640. https://doi.org/10.2337/dc17-1600.

76. Tarnopolsky, M.A., Pearce, E., Smith, K., and Lach, B. (2011). Suction-modified Bergström muscle biopsy technique: experience with 13,500 procedures. Muscle Nerve *43*, 717–725.

77. Cole, T.G. (1990). Glycerol blanking in triglyceride assays: is it necessary? Clin. Chem. *36*, 1267–1268.

78. Julkunen, H., Cichonska, A., Tiainen, M., Koskela, H., Nybo, K., Makela, V., Nokso-Koivisto, J., Kristiansson, K., Perola, M., Salomaa, V., et al. (2023). Atlas of plasma NMR biomarkers for health and disease in 118,461 individuals from the UK Biobank. Nat. Commun. *14*, 604. https://doi.org/10.1038/s41467-023-36231-7.

79. Skogerboe, K.J., Labbe, R.F., Rettmer, R.L., Sundquist, J.P., and Gargett, A.M. (1990). Chemiluminescent measurement of total urinary nitrogen for accurate calculation of nitrogen balance. Clin. Chem. *36*, 752–755.

80. Jequier, E., and Felber, J.P. (1987). Indirect calorimetry. Baillieres Clin Endocrinol Metab *1*, 911–935. https://doi.org/10.1016/s0950-351x(87)80011-3.

81. Lim, M.Y., Song, E.J., Kim, S.H., Lee, J., and Nam, Y.D. (2018). Comparison of DNA extraction methods for human gut microbial community profiling. Syst. Appl. Microbiol. *41*, 151–157. https://doi.org/10.1016/j.syapm.2017.11.008.

82. Langmead, B., and Salzberg, S.L. (2012). Fast gapped-read alignment with Bowtie 2. Nat. Methods *9*, 357–359. https://doi.org/10.1038/nmeth.1923.

83. Kreuger, F. (2021). Trimgalore. https://github.com/FelixKrueger/TrimGalore.

84. Lu, J., Breitwieser, F.P., Thielen, P., and Salzberg, S.L. (2017). Bracken: estimating species abundance in metagenomics data. Peerj Comput Sci. *3*, e104. https://doi.org/10.7717/peerj-cs.104.

85. Wood, D.E., Lu, J., and Langmead, B. (2019). Improved metagenomic analysis with Kraken 2. Genome Biol. *20*, 1–13. https://doi.org/10.1186/s13059-019-1891-0.

86. Franzosa, E.A., McIver, L.J., Rahnavard, G., Thompson, L.R., Schirmer, M., Weingart, G., Lipson, K.S., Knight, R., Caporaso, J.G., Segata, N., and Huttenhower, C. (2018). Species-level functional profiling of metagenomes and metatranscriptomes. Nat. Methods *15*, 962–968. https://doi.org/10.1038/s41592-018-0176-y.

87. Zhang, S., Wang, H., and Zhu, M.J. (2019). A sensitive GC/MS detection method for analyzing microbial metabolites short chain fatty acids in fecal and serum samples. Talanta *196*, 249–254. https://doi.org/10.1016/j.talanta.2018.12.049.

88. Boets, E., Gomand, S.V., Deroover, L., Preston, T., Vermeulen, K., De Preter, V., Hamer, H.M., Van den Mooter, G., De Vuyst, L., Courtin, C.M., et al. (2017). Systemic availability and metabolism of colonic-derived short-chain fatty acids in healthy subjects: a stable isotope study. J. Physiol. *595*, 541–555. https://doi.org/10.1113/JP272613.

89. Hewavitharana, A.K. (2011). Matrix matching in liquid chromatography-mass spectrometry with stable isotope labelled internal standards–is it necessary? J. Chromatogr. A *1218*, 359–361. https://doi.org/10.1016/j.chroma.2010.11.047.

90. Harris, R.C., Hultman, E., and Nordesjo, L.O. (1974). Glycogen, glycolytic intermediates and high-energy phosphates determined in biopsy samples of musculus quadriceps femoris of man at rest. Methods and variance of values. Scand. J. Clin. Lab. Invest. *33*, 109–120.

91. Faul, F., Erdfelder, E., Lang, A.G., and Buchner, A. (2007). G*Power 3: a flexible statistical power analysis program for the social, behavioral, and biomedical sciences. Behav. Res. Methods *39*, 175–191.

92. Narang, B.J., Atkinson, G., Gonzalez, J.T., and Betts, J.A. (2020). A Tool to Explore Discrete-Time Data: The Time Series Response Analyser. Int. J. Sport Nutr. Exerc. Metabol. *30*, 374–381. https://doi.org/10.1123/ijsnem.2020-0150.

93. Bland, J.M., and Altman, D.G. (2015). Best (but oft forgotten) practices: testing for treatment effects in randomized trials by separate analyses of changes from baseline in each group is a misleading approach. Am. J. Clin. Nutr. *102*, 991–994. https://doi.org/10.3945/ajcn.115.119768.

94. Bender, R., and Lange, S. (2001). Adjusting for multiple testing–when and how? J. Clin. Epidemiol. *54*, 343–349. https://doi.org/10.1016/s0895-4356(00)00314-0.

95. Blundell, J., De Graaf, C., Hulshof, T., Jebb, S., Livingstone, B., Lluch, A., Mela, D., Salah, S., Schuring, E., Van Der Knaap, H., and Westerterp, M. (2010). Appetite control: methodological aspects of the evaluation of foods. Obes. Rev. *11*, 251–270.

96. Smirnova, E., Huzurbazar, S., and Jafari, F. (2019). PERFect: PERmutation Filtering test for microbiome data. Biostatistics *20*, 615–631. https://doi.org/10.1093/biostatistics/kxy020.

97. Park, C.C., and Allaby, M. (2017). A dictionary of environment and conservation. Edition, Third edition (Oxford University Press).

98. Martino, C., Morton, J.T., Marotz, C.A., Thompson, L.R., Tripathi, A., Knight, R., and Zengler, K. (2019). A Novel Sparse Compositional Technique Reveals Microbial Perturbations. mSystems *4*, e00016-19. https://doi.org/10.1128/mSystems.00016-19.

99. Oksanen, J., Simpson, G., Blanchet, F., Kindt, R., Legendre, P., Minchin, P., O'Hara, R., Solymos, P., Stevens, M., and Szoecs, E. (2022). vegan: Community Ecology Package, R package version 2.6-4. https://CRAN.R-project.org/package=vegan.

100. Fernandes, A.D., Reid, J.N., Macklaim, J.M., McMurrough, T.A., Edgell, D.R., and Gloor, G.B. (2014). Unifying the analysis of high-throughput sequencing datasets: characterizing RNA-seq, 16S rRNA gene sequencing and selective growth experiments by compositional data analysis. Microbiome *2*, 15. https://doi.org/10.1186/2049-2618-2-15.

## STAR★METHODS

### KEY RESOURCES TABLE

| REAGENT or RESOURCE | SOURCE | IDENTIFIER |
|---|---|---|
| Antibodies | | |
| INSR | Cell Signaling | 3025 |
| IRS1 | Merk | 2387826 |
| Akt | Cell Signaling | 3063 |
| AS160 | Cell Signaling | 2670 |
| AMPK | Cell Signaling | 2532 |
| GLUT4 | Custom made antibody | N/A |
| TGR5 | ABCAM | Ab72608 |
| PLIN | Cell Signaling | 9349 |
| FFAR2 | ABCAM | Ab131003 |
| FFAR3 | ABCAM | Ab236654 |
| PDK4 | ABGENT | Q16654 |
| CPT1B | ABCAM | Ab134988 |
| Anti-rabbit IgG-HRP conjugate | Invitrogen | 16104 |
| Anti-mouse IgG-HRP conjugate | Invitrogen | 31432 |
| Biological samples | | |
| Human serum and plasma | This paper | N/A |
| Human adipose tissue | This paper | N/A |
| Human skeletal muscle tissue | This paper | N/A |
| Human urine samples | This paper | N/A |
| Microbial DNA from human fecal samples | This paper | N/A |
| Chemicals, peptides, and recombinant proteins | | |
| Hexokinase | Sigma-Aldrich | H4502 |
| Glucose-6-phosphate dehydrogenase | Sigma-Aldrich | G5885 |
| α-amyloglucosidase | Sigma-Aldrich | 10115 |
| Amersham™ ECL Prime Western Blotting Detection Reagent | Cytiva | RPN2232 |
| Amersham™ ECL Select Western Blotting Detection Reagent | Cytiva | RPN2235 |
| Critical commercial assays | | |
| RX Daytona glucose | Randox | GL3815 |
| RX Daytona lactate | Randox | LC3980 |
| RX Daytona triglycerides | Randox | TR3823 |
| RX Daytona non-esterified fatty acids | Randox | FA 115 |
| RX Daytona glycerol | Randox | GY 105 |
| RX Daytona beta-hydroxybutyrate | Randox | RB1007 |
| RX Daytona total cholesterol | Randox | CH3810 |
| RX Daytona LDL-cholesterol (direct) | Randox | CH3841 |
| RX Daytona HDL-cholesterol | Randox | CH3811 |
| RX Daytona apolipoprotein B | Randox | LP3839 |
| RX Daytona urea | Randox | UR3825 |
| RX Daytona albumin | Randox | AB3800 |
| Insulin ELISA | Mercodia | 10-1113-10 |
| Leptin ELISA | Mercodia | 10-1199-01 |
| Lipopolysaccharide binding protein ELISA | R&D Systems | DY870-05 |

(*Continued on next page*)

| REAGENT or RESOURCE | SOURCE | IDENTIFIER |
|---|---|---|
| *Continued* | | |
| Fibroblast growth factor 21, Ghrelin (total), C-reactive protein, C-peptide, GLP-1 (total) U-PLEX Metabolic combo 1 (human) multiplex assay | Meso Scale Discovery | K15281K |
| U-PLEX Human CRP assay | Meso Scale Discovery | K151L9K |
| QIAamp Fast DNA Stool Mini Kit | Qiagen | 51504 |
| miRNeasy® mini column for extraction | Qiagen | 1038703 |
| RNase-Free DNase set | Qiagen | 79254 |
| Nextera XT DNA Library Preparation Kit | Illumina Inc. | FC-131-1096 |
| DNA/RNA UD Indexes | Illumina Inc. | 20027217 |
| Agilent High Sensitivity DNA Kit | Agilent Technologies | 5067–4626 |
| Qubit dsDNA high-sensitivity assay | Thermo Fisher Scientific | Q32851 |
| KAPA Library Quantification Kit | Kapa Biosystems | KK4824 |
| 2 × 150 bp High Output Kit | Illumina Inc. | 20024908 |
| Applied Biosystems™ High-capacity cDNA reverse transcription kit | Applied Biosystems | 4368814 |
| Luna® Universal qPCR Master Mix | New England Biolab | M3003E |
| Deposited data | | |
| Raw human data | Mendeley Data | https://data.mendeley.com/v1/datasets/10.17632/g5yn28myjb.2 |
| Raw microbiome metagenomic data | European Nucleotide Archive | PRJEB72300 |
| Raw NMR metabolomics data | Mendeley Data | https://data.mendeley.com/v1/datasets/10.17632/g5yn28myjb.2 |
| Oligonucleotides | | |
| Primers used for RNA expression | Merck | See Table S10 |
| Software and algorithms | | |
| Prism | GraphPad | 9.2.0 |
| SPSS | IBM | 29.0.1.0 |
| R | R | 4.1.0 |
| Code | Github | https://github.com/jg833/CHEBI.git |
| Biorender | Biorender | biorender.com |

## RESOURCE AVAILABILITY

### Lead contact

Further information and requests for resources and reagents should be directed to and will be fulfilled by the lead contact, Professor Javier Gonzalez (J.T.Gonzalez@bath.ac.uk).

### Materials availability

This study did not generate new unique reagents.

### Data and code availability

De-identified data will be publicly available as of the date of publication at Mendeley Data: https://data.mendeley.com/v1/datasets/10.17632/g5yn28myjb.2 and the European Nucleotide Archive: https://www.ebi.ac.uk/ena/browser/(data accession ID: PRJEB72300). This paper does not report original code. Accession numbers are listed in the key resources table. Any additional information required to reanalyze the data reported in this work paper is available from the lead contact upon request.

## EXPERIMENTAL MODEL AND STUDY PARTICIPANT DETAILS

This study included human participants from the local area of Bath (UK; NCT03574987). Data were collected in accordance with the Declaration of Helsinki. The study was approved by the South West – Central Bristol National Health Service (NHS) Research Ethics

Committee (18/SW/0178). The study sponsor was the University of Bath. Participant characteristics are presented in Table S1. Inclusion criteria were body mass index (BMI) between 18.5 and 29.9 kg $m^{-2}$, age between 18 and 65 years, able and willing to provide informed consent and safely comply with study procedures, no anticipated changes in physical activity during the study period (e.g., specific training programs or holidays), maintaining a record of regular menstrual cycle phase or contraceptive use (females only). Exclusion criteria were any reported condition or behavior deemed either to pose undue personal risk to the participant or introduce bias into the experiment, any diagnosed metabolic disease (e.g., type 1 or type 2 diabetes), any reported use of substances which may pose undue personal risk to the participant or introduce bias into the experiment, lifestyle not conforming to a standard sleep-wake cycle (e.g., night-shift workers), any reported recent (<6 months) change in body mass greater than 3%, use of antibiotic medication in the previous 3 months, and use of prebiotic or probiotic supplements or products in the previous month. Sex was classified based on sex chromosomes.

## METHOD DETAILS

### Study protocol

Sixty participants were randomized to one of three intervention diets which differed in macronutrient profile for 12 weeks, in an open-label study with three active comparators, MODSUG as control. A consort diagram displaying participant flow through the intervention can be found in Figure 1A. Fifty-three participants received allocation of the intervention for $\geq$4 weeks. Participants completed 1 week of habitual diet and physical activity monitoring before allocation to the intervention diet, with laboratory visits at baseline, 4 weeks, and 12 weeks. Laboratory visits took place across two consecutive days, with an afternoon session on day one comprising tissue biopsies and a treadmill walk and a morning session on day two following an overnight fast comprising assessments of body composition, resting metabolic rate, and physiological responses to a mixed meal tolerance test.

### Preliminary measures

Preliminary measures included anthropometric measures, resting metabolic rate (RMR), and physical activity monitor calibration during an initial laboratory visit following a minimum of 4–5 h fast. Participants were provided with a physical activity monitor and asked to record habitual diet for 7 days. One week prior the baseline laboratory visit, participants were fitted with a continuous glucose monitor (CGM) (Freestyle Libre Pro, Abbott, UK) to capture habitual lifestyle and week one of the intervention.

### Randomization

Participants were randomized according to a randomization plan developed by the trial statistician (J.A.B.), with randomization performed by a researcher who was not involved in participant interaction (F.K.). Participants were randomized in block sizes of 6, in a 1:1:1 ratio to each of the three arms of the study with allocation stratified on two levels: by self-reported sex (male vs. female) and habitual physical activity level (PAL) during preliminary measures (<1.70 vs. $\geq$ 1.70). PAL was calculated by dividing total energy expenditure (TEE) by RMR (TEE was calculated as measured RMR plus measured PAEE, multiplied by 1.1 to account for diet-induced thermogenesis).

### Laboratory visits

Laboratory visits occurred at baseline, week 4, and week 12 of the intervention and were split across two consecutive days. On day one, participants arrived in the afternoon following a mean (SD) 5.7 (1.6) hour fast having not performed structured exercise in this period. An adipose tissue biopsy and a muscle tissue biopsy were obtained. After a short rest (5–10 min), participants completed a graded exercise protocol on the treadmill before returning home.

On day two, participants arrived at around 08:00, following a mean (SD) overnight fast of 13.8 (4.2) hours, having drank a pint of water and avoided strenuous physical activity. A whole-body dual-energy X-ray absorptiometry (DXA) scan was performed, and anthropometric measures were obtained, followed by resting metabolic rate (RMR) and blood pressure. An intravenous cannula was inserted for collection of arterialized blood samples. A baseline blood sample was obtained, and participants were provided with a mixed-meal tolerance test (Chocolate Ensure Plus, Abbott, USA) equating to 30% of RMR. The composition of the test solution closely aligned the prescribed nutrient intake of the MODSUG group; 54% carbohydrate (23% of which sugars), 31% fat, 15% protein. Participants consumed the whole drink within 5 min.

Blood samples were obtained 15-, 30-, 45-, 60-, 90-, 120, 150-, 180-, 210- and 240-min following the first sip. A 5-min expired gas sample was collected hourly to measure postprandial energy expenditure and substrate oxidation. Blood pressure was also measured hourly. Urine produced during the laboratory visit was collected and urea concentrations were analyzed.

### Design of the diets

The moderate-sugar (MODSUG) control diet was designed to be reflective of macronutrient and sugar intake in high-income countries.[56–58] The low-sugar (LOWSUG) diet was designed to meet public health guidelines advocating reduced free-sugar intake to <5% of total energy intake.[1,59] The low-carbohydrate (LOWCHO) diet was designed to restrict carbohydrate availability (<8% of energy) and promote ketogenesis, in line with the definition of a 'very low-carbohydrate ketogenic diet'.[60]

### Implementation and adherence

Participants were provided with feedback about habitual diet roughly one week prior to the baseline visit. For the MODSUG and LOWSUG groups, sources of free sugars were identified and a target in grams per day was provided to participants on an individual basis and adjusted weekly to achieve close to the 20% and below 5% targets respectively. For the LOWCHO group, sources of carbohydrates were identified, and advice was provided to reduce intake to below 50 g per day or 8% of habitual energy intake, whichever was lowest, based on recommendations to stick to a very-low carbohydrate ketogenic diet.[61] Participants in the LOWCHO group received additional posters and website recommendations to help them achieve the dietary targets.

Participants met with the lead researchers frequently throughout the intervention to improve adherence. Participants were required to record 7-day of diet data in the baseline period plus 7 days during week 4 and week 12, in addition to 3 days per week for all other weeks, which equates to 56% of all study days being recorded throughout the intervention. Adherence to this was good and participants often recorded dietary data above the required minimum, resulting in mean (SD) days of full food diary reported for each group of 59% (20%) for MODSUG, 60% (17%) for LOWSUG, and 70% (22%) for LOWCHO.

Participants received weekly feedback on macronutrient intake with total energy intake hidden using nutrition analysis software (Nutritics, Ireland). Participants sent a weekly measure of body mass using provided scales (Etekcity Digital Scales, USA) and fasting urinary acetoacetate concentrations (Ketostix, Ascencia Diabetes Care Holdings AG, Switzerland). Participants were partially reimbursed to reduce financial burden of participation; £18/week for MODSUG and LOWSUG, £26/week for LOWCHO, due to the relatively larger change in diet (thus inconvenience) compared to habitual. Other outcome measures indicating adherence included rearranging the energy balance equation, fasting serum beta-hydroxybutyrate (βOHB) concentrations during laboratory visits, and measures of respiratory exchange ratio (RER). Participants also wore a CGM for the first week and final 2 weeks of the intervention as an outcome measure and indicator of adherence.

### Physical activity

Free-living physical activity energy expenditure (PAEE) was measured using Actiheart 4 monitors (CamNtech Ltd., UK) attached to a modified heart rate strap. Activity monitors were individually calibrated using a treadmill exercise test modified from Brage et al.[62] Participants completed a walk consisting of four 5-min stages at 5.2 km $h^{-1}$ at progressive inclines of 0%, 3%, 6%, and 9%. In the last minute of each 5-min stage, heart rate was recorded at 11-, 22-, 33-, and 44- seconds, and expired breath was collected into a Douglas bag to measure energy expenditure according to the methods for indirect calorimetry. The speed and/or incline of the protocol were adjusted to reduce the intensity of the protocol for participants who felt unable to complete the protocol as prescribed. Heart rate and energy expenditure were plotted to enable linear interpolation of heart rates at 10 beat intervals and this information was entered into software v4.0.116 (CamNtech Ltd., UK). Physical activity was recorded in 1-min epochs and organized into intensity thresholds. Thresholds for physical activity intensities were defined and calculated for each participant as sedentary <1.5 METs, light ≥1.5 to <3.0 METs, moderate ≥3.0 to <6.0 METs, vigorous ≥6.0 to <10.2 METs, and very vigorous ≥10.2 METs.[63,64] Data were visually inspected to determine wear time and data loss. Days with substantial data loss (<40% trace available) were omitted from analyses. Percentage of 'interpolated' or 'lost' data was recorded. Sleeping heart rate was defined as the highest value of the lowest 30 min-by-minute heart rate reading across 24 h. Total daily physical activity was the primary outcome. To mitigate against bias, participants were not told that physical activity was the primary outcome and were told instead to focus on achieving diet targets and told not to explicitly engage in any new exercise programs.

### Anthropometry and body composition

Height was measured to the nearest 0.1 cm (Seca Ltd., Germany). Body mass was measured to the nearest 0.1 kg using digital weighing scales (TANITA Corp., Japan). Waist and hip circumference were measured to the nearest 0.5 cm using a handheld tape measure (Seca Ltd., Germany). Whole-body dual energy X-ray absorptiometry (DXA) scans were used to assess body composition (QDR Discovery W, Hologic, UK). For weekly body mass measures outside of the laboratory, all participants were provided with a set of bathroom scales (Etekcity, USA) following demonstration of correct use in the laboratory and were asked to send photographs of their measurements.

### Indirect calorimetry and substrate oxidation

Expired gas samples were collected into Douglas bags through a mouthpiece with participants wearing a nose clip (Hans Rudolph, USA). Expired fractions of $O_2$ and $CO_2$ were determined using paramagnetic and infrared gas analyzers (Mini HF 5200, Servomex Group Ltd., UK) and volume of expired gas was measured using a dry gas meter (Harvard Apparatus, UK), with volume of inspired air calculated using the Haldane transformation.[65] Concurrent concentrations of ambient O2 and CO2 were measured to account for fluctuations with inspired air.[66] Ambient temperature and saturated barometric pressure were measured to correct to standard temperature and pressure (dry). The gas analyzer was calibrated prior to each participant visit using a 2-point calibration of known gases; a low concentration gas of 0% O2 and 0% CO2 (99.99% grade Nitrogen) and a high concentration gas circa 20% O2 and 8% CO2 (BOC Ltd, UK).

Energy expenditure and substrate oxidation were determined using equations derived from Frayn and Jeukendrup & Wallis,[67,68] with adjustments for the contribution of glycogen during low-intensity exercise:

$$\text{Fat oxidation at rest and during exercise } (g \cdot min - 1) = (1.695 \text{ x } \dot{V}O2) - (1.701 \text{ x } \dot{V}CO2) \quad \text{(Equation 1)}$$

$$\text{Carbohydrate oxidation at rest } (g \cdot min - 1) = \left(4.55 \text{ x } \dot{V}CO2\right) - \left(3.21 \text{ x } \dot{V}O2\right) \quad \text{(Equation 2)}$$

$$\text{Carbohydrate oxidation during exercise } (g \cdot min - 1) = \left(4.344 \text{ x } \dot{V}CO2\right) - \left(3.061 \text{ x } \dot{V}O2\right) \quad \text{(Equation 3)}$$

At rest, these equations assume that glucose provides all carbohydrate for metabolism, whereas during low-intensity exercise carbohydrate metabolism is achieved by an equal contribution from glucose and glycogen. These equations were used with the assumption that the energy yields from fat, glucose, and glycogen are 9.75, 3.74, and 4.15 kcal $g^{-1}$ respectively.[68]

## Dietary assessment and energy intake

Participants were provided with portable weighing scales (SmartWeigh, China). For habitual assessment of energy and nutrient intake they recorded all food and caloric beverages ingested for 7 consecutive days. Throughout the intervention, participants recorded a minimum of 3 days each week other than weeks where 7 days were required (baseline, week 4, week 12). Participants chose whether they recorded diet with a paper food diary or by entering data into the Libro app on ‘blind mode’ (Nutritics, Ireland). Paper food diaries were manually entered and Libro diaries were exported directly into diet analysis software (Nutritics, Ireland). Data were exported and used to evaluate macronutrient composition and provide an estimate of energy intake with the assumed caloric values of each nutrient to be: starch (4.18 kcal $g^{-1}$, 17.5 kJ $g^{-1}$), sucrose (3.94 kcal $g^{-1}$, 16.5 kJ $g^{-1}$), lactose (3.94 kcal $g^{-1}$, 16.5 kJ $g^{-1}$), fiber (1.84 kcal $g^{-1}$, 7.7 kJ $g^{-1}$), fat (8.94 kcal $g^{-1}$, 37.4 kJ $g^{-1}$), protein (4.02 kcal $g^{-1}$, 16.8 kJ $g^{-1}$), and alcohol (7.07 kcal $g^{-1}$, 29.6 kJ $g^{-1}$).[69] Sugars assigned the caloric value of 3.94 kcal $g^{-1}$ were further partitioned into contributions from ‘fruit and vegetable sugars’, ‘liquid free-sugars’, ‘solid free-sugars’, and ‘milk sugars’ by visual inspection of food diaries. Fat assigned the value of 8.94 kcal $g^{-1}$ was partitioned into ‘saturated fat’ and ‘other fats’ by subtracting the value for saturated fat obtained from the nutrition analysis software by the total amount for fat. Foods included in the definition of ‘liquid free-sugars’ were sugar-sweetened beverages, fruit juice, smoothies (homemade or branded), non-dairy milks (e.g., almond milk), flavored milks, alcoholic beverages, soups, milkshakes, and lassi. Excluded from this definition (i.e., categorized as solid free sugars) were passata, tinned goods (e.g., baked beans), yoghurts, condiments (e.g., tomato ketchup and hoisin sauce), kefir, energy gels, cream, syrups, honey, ice cream, and custard. This is based on guidance on adhering to SACN guidelines for reporting.[2]

A second assessment of energy intake was calculated by rearranging the energy balance equation, using measures of fat mass and fat free mass obtained from DXA scans, as validated previously (Racette et al., 2012; Sanghvi et al., 2015). It is worth noting that the energy expenditure values were extrapolated from 7 days of physical activity recording to reflect the time between measurements. The equations used were:

$$\text{Energy intake } \left(\text{Kcal } d^{-1}\right) = \frac{\Delta \text{ Energy stores } \left(\text{Kcal } d^{-1}\right)}{\Delta \text{ time } (d)} \quad \text{(Equation 4)}$$

Where:

$$\Delta \text{ Energy stores (kcal)} = \left(\Delta \text{ fat mass g} \times 9.3 \left(\text{kcal} \cdot g^{-1}\right)\right) + \left(\Delta \text{ fat free mass g} \times 1.1 \left(\text{kcal} \cdot g^{-1}\right)\right) \quad \text{(Equation 5)}$$

## DIT and food quotient

Dietary induced thermogenesis was estimated where appropriate using average values for each macronutrient of 7.5% for carbohydrate, 2% for fat, 25% for protein, and 20% for alcohol.[70] Food quotient was estimated using an equation presented by Hall et al.[50] with the addition of alcohol[71]:

$$\text{Food quotient} = \frac{(\text{CHO} \times 1.0) + (\text{FAT} \times 0.71) + (\text{PRO} \times 0.835) + (\text{EtOH} \times 0.67)}{\text{Total energy intake}} \quad \text{(Equation 6)}$$

Where CHO is energy from carbohydrate intake, FAT is energy from fat intake, PRO is energy from protein intake, and EtOH is energy from alcohol intake.

## Food preference, appetite, and mood

An ‘alternative forced choice task’ was used to assess food preference. The choice task consisted of 18 plates of food individually photographed on a white plate or transparent bowl. Two foods appeared on the screen and participants were asked to select which food they would ‘choose to eat right now’. New combinations of foods appeared until participants had chosen between all possible combinations. Foods were distinguished into three categories, matched at six levels of energy density: sweet high-carbohydrate

foods, non-sweet high-carbohydrate foods, non-sweet low carbohydrate foods (Table S9). Relative preference for these 3 food categories was measured out of 100%. Statements related to appetite and mood were measured using visual analogue scales (VAS). Each scale had a statement followed with a 100 mm horizontal line with ‘Not at all’ at 0 mm and ‘Extremely’ at 100 mm.

### Submaximal exercise test

Participants completed graded exercise protocols on the treadmill at baseline, week 4, and week 12. This enabled measurement of changes to energy expenditure and substrate oxidation throughout, but also mirrored the Actiheart calibration protocol to allow re-calibration of the Actiheart to potential individual changes in the heart rate/energy expenditure relationship across the study. The test comprised four 5-min walking stages with treadmill speed fixed at 5.2 km $h^{-1}$ and gradients of 0%, 3%, 6%, and 9%. During the last minute of each stage expired air was obtained to measure energy expenditure and substrate oxidation, heart rate was measured at 11-, 22-, 33-, and 44-s using a heart rate monitor (Polar Electro, UK), and ratings of perceived exertion (RPE) were measured using Borg’s 6–20 scale.[72]

### Blood pressure and heart rate

Systolic and diastolic blood pressure, and heart rate, were measured using an automated monitor (Diagnostec EW3106, Panasonic, Japan) with participants resting for at least 10 min in a semi-supine position.

### Continuous glucose monitoring

Interstitial glucose concentrations were measured using continuous glucose monitors (CGMs) (Freestyle Libre Pro, Abbott Diabetes Care Ltd., UK) with participants blinded to glucose readings. Mean interstitial glucose concentrations for the wear period were calculated to reduce day-to-day within participant variability. Summative indices included days in use (n), time active (%), mean glucose (mmol·$L^{-1}$), glucose management indicator (%), glycemic variability (CV %), and time in range (%) as recommended by consensus statements.[73–75] Thresholds for time in range were based on consensus recommendations.[73] Glucose management indicator (GMI) is a method of estimating glycated hemoglobin concentrations from CGM.[74]

### Adipose tissue biopsies

Subcutaneous abdominal adipose tissue biopsies were obtained lateral to the umbilicus via needle aspiration. The abdominal region was sterilized with Videne (10% [w/w] Povidone-Iodine giving 1% [w/w] available Iodine; Ecolab; UK) and ~5 mL anesthetic (1% lidocaine hydrochloride, Hameln Pharmaceuticals, UK) was administered subcutaneously in the local area. A 14 G needle (Monoject, Covidien, Ireland) attached to a 50 mL syringe (BD, USA) with ~10 mL sterile saline (0.9% Sodium Chloride) solution (B. Braun, USA) was inserted into the area and a vacuum was created. Samples were cleaned on 100 μm gauze with 0.9% Sodium Chloride solution (B. Braun, USA), weighed to the nearest 0.1 mg, and snap-frozen in liquid nitrogen.

### Skeletal muscle biopsies

Skeletal muscle biopsies were obtained from the *vastus lateralis* using the Bergstrom needle technique with suction.[76] The quadriceps area was sterilized with Videne (10% [w/w] Povidone-Iodine giving 1% [w/w] available Iodine; Ecolab; UK) and anesthetic (1% lidocaine hydrochloride, Hameln Pharmaceuticals, UK) was administered subcutaneously and around the muscle fascia. A small (~0.5 cm) incision was made through skin and muscle fascia using a scalpel blade (Swann Morton, UK). A sterile Bergstrom needle was inserted into the muscle belly and 2–3 snips were made with suction applied through a 100 mL syringe (BD, USA) attached to the needle via a sterile catheter (Pennine Healthcare, UK). Muscle tissue samples were snap-frozen in liquid nitrogen and weighed to the nearest 0.1 mg.

### Blood sample processing and analyses

Blood samples were collected into two tubes: EDTA and clotting activator (Sarstedt, Germany) for the collection of blood plasma and serum. EDTA tubes were centrifuged immediately, and clotting activator tubes left at room temperature for 15 min to clot before being centrifuged. Tubes were centrifuged at 4000 × g for 10 min at 4°C. Aliquots were placed on dry ice and stored at −80°C.

Serum glucose, triglycerides (TAG), glycerol, non-esterified fatty acids (NEFA), lactate, beta-hydroxybutyrate (βOHB), total cholesterol, high-density lipoprotein cholesterol (HDL-C), and low-density lipoprotein cholesterol (LDL-C), apolipoprotein B (ApoB), urea, and albumin concentrations were measured using an automated analyzer (RX Daytona, Randox Laboratories, UK) using commercially available kits for each analyte (Randox Laboratories, UK). Reported TAG values in the present paper have been blanked for glycerol based on recommendations for clinical research.[77] Serum insulin and leptin were measured using enzyme-linked immunosorbent assay (ELISA) kits (Mercodia AB, Sweden). Serum lipopolysaccharide binding protein (LBP) was measured using a commercially available ELISA kit (R&D Systems, USA). All ELISA plates were quantified using a SPECTROstar Nano plate reader (BMG Labtech, Germany). Fibroblast growth factor 21 (FGF21), C-peptide, ghrelin, total glucagon-like peptide 1 (GLP-1), and C-reactive protein (CRP) were measured on a QuickPlex SQ 120 (Meso Scale Discovery Inc, USA) using commercially available electro-chemiluminescent multiplex kits (Meso Scale Discovery Inc, USA). All samples for a participant were measured on the same run or plate. Samples producing values below the lower limit of detection were assigned the value of the lower detectable concentration, which was necessary for some samples with βOHB, insulin, and leptin.

Fasting plasma samples from baseline and week 4 were analyzed using targeted high-throughput nuclear magnetic resonance (NMR) spectroscopy (Nightingale Health Ltd., Finland), described in detail by Julkunen et al.[78] Week 4 was chosen based on higher sample size and compliance at this time-point, as well as cost constraints.

### Urine sample processing and analyses

Urine samples were collected by participants during laboratory visits. The first sample was aliquoted and stored on dry ice. Then 5 mL of 10% thymol-isopropanol was added to the remainder of the sample as a preservative. Urine samples were combined and mixed, a 1.5 mL sample was stored, and total urinary volume was measured and discarded. Urinary urea concentrations were measured using commercially available assay kits on an automated analyzer (RX Daytona, Randox Laboratories Ltd., UK). Urinary urea was assumed to represent ~90% of urinary nitrogen excretion.[79] Plasma urea concentrations were also measured in the baseline and 240-min samples. These data were used to estimate the rate of protein oxidation.[80]

### Fecal sample collection

Fecal samples were collected by participants within 24 h of each laboratory visit. Samples were transported to the laboratory in an opaque cooler bag containing a frozen icepack. Fecal samples were homogenized, aliquoted, and stored at −80°C for later processing.

### Microbiome analyses

Total DNA was extracted from frozen human stool samples using the QIAamp Fast DNA Stool Mini Kit (Qiagen, Germany) with the addition of a bead beating step.[81] To minimize thawing of stool samples prior to addition of lysis buffer, ~200 mg of homogenized stool was stored directly in autoclaved bead-beating tubes and frozen at −80°C ready for DNA extraction. Tubes were placed in a Precellys Evolution homogenizer (Bertin Technologies, France) and underwent bead beading for 3 min at 6,000 rpm. Some participants were unable or unwilling to provide fecal samples and therefore microbiome data are presented for $n$ = 48 of 53 participants (91%) at baseline and Week 4 and $n$ = 41 of 46 participants (89%) at Week 12.

Extracted fecal DNA was prepared for sequencing using the Nextera XT DNA Library Preparation Kit (Illumina Inc., USA). Tagmentation and amplification were completed on an Applied Biosystems 2720 thermal cycler (Thermo Fisher Scientific, USA). Prior to amplification, manufacturer recommended DNA/RNA UD Indexes (Illumina Inc., USA) were added to each sample. After clean-up of the libraries, fragment size ranges of individual samples were checked using the Agilent High Sensitivity DNA Kit (Agilent Technologies, USA) on the Agilent 2100 Bioanalyzer (cat. G2939BA, Agilent Technologies, USA). The DNA concentration of each sample was determined using the Qubit dsDNA high-sensitivity assay (Thermo Fisher Scientific, USA) on the Qubit 4 Fluorometer (cat. Q33238, Thermo Fisher Scientific, USA). Based on these metrics the libraries were pooled at equal molarity, and the concentration of the final pooled libraries was determined by qPCR using the KAPA Library Quantification Kit (Kapa Biosystems, USA) on the LightCycler 480 (cat. 05015278001, Roche, Switzerland). The pooled libraries were sequenced with the 2 × 150 bp High Output Kit (cat. 20024908, Illumina Inc., USA) on the Illumina NextSeq 500 sequencing system.

Contaminating host (human) reads were removed using a reference library in Bowtie2 (version 2.3.4).[82] Resulting FastQ files were trimmed and quality filtered using Trim Galore (phred = 33, minimum length = 20).[83] Taxonomic classifications of trimmed reads were determined using Kraken2 and Bracken using the GTDB database (database downloaded June 2020).[84,85] Functional profiling was completed using the Human Microbiome Project Unified Metabolic Analysis Network 2 (HUMAnN2).[86] A total of $3.64 \times 10^8$ trimmed reads were used for classification after contaminant removal, averaging $7.92 \times 10^6$ reads per sample.

### Short-chain fatty acid measurement

The short-chain fatty acids acetate (C2), propionate (C3) and butyrate (C4) in plasma samples were measured using gas chromatography-mass spectrometry (GC-MS) following a modified version of the protocol developed by Zhang et al.[87] Concentrations of propionate and butyrate are low in human plasma,[88] and so modifications were made to the protocol to improve sensitivity and to ensure the correct analytes were quantified. A key modification made to the protocol was the use of a separate 13C labeled internal standard for each SCFA and calibrations were also based on internal standards which accounts for variation in sample preparation and matrix effects.[89]

The following internal standards were purchased from MilliporeSigma (USA): acetic acid-13C2 (cat. no. 282022), propionic acid-1-13C (cat. no. 2824448), and butyric acid-1,2-13C2 (cat. no. 491993). For each sample 200 μL of plasma was diluted 1:1 in HLPC grade water and added to 50 μL of 5 M hydrochloric acid to bring the pH to 2. Subsequently, 64 μL of internal standard mixture containing acetic acid-13C2 at 400 μM, propionic acid-1-13C at 40 μM and butyric acid-1,2-13C2 at 40 μM was added, resulting in a final concentration of 97 μM acetic acid-13C2, 9.7 μM propionic acid-1-13C and 9.7 μM butyric acid-1,2-13C2. For SCFA extraction, 500 μL of HPLC grade anhydrous diethyl ether was added to each sample. Samples were vortexed, incubated for 5 min at 4°C and then centrifuged at 10,000 × g for 5 min at 4°C. The supernatant diethyl ether layer from each sample was then transferred to a new 1.5 mL tube containing ~200 mg sodium sulfate to absorb any transferred water. The extraction process was repeated twice more to pool ~1.2 mL of diethyl ether containing SCFA in the tubes containing sodium sulfate, then 250 μL was transferred to a glass insert and 20 μL of N,O-Bis(trimethylsilyl)trifluoroacetamide (BSTFA) derivatization reagent was added. Samples were vortexed and incubated at 37°C for 2 h.

Samples were analyzed on an 8890 GC system with a 5977B mass selective detector (Agilent, USA) and an HB-5 ms capillary column (30 m × 0.25 mm × 0.25 μm film thickness) (Agilent, USA). The injector temperature was 260°C, the ion source temperature was 230°C, the quadrupole temperature was 150°C, and the GC/MS interface temperature was 280°C. Helium carrier gas flow rate was 1 mL/min throughout. After a 3 min solvent delay 2 μL of sample was injected without splitting. Column temperature was initially 40°C held for 2 min, then increased to 150°C by 15°C/min, held for 1 min and then further increased to 300°C by 30°C/min, then held for 5 min. Ionization was at 70 eV in electron impact mode. Analyte and internal standard responses were quantified in selected ion monitoring (SIM) mode. Target ions for acetic, propionic, and butyric acids were 117, 131 and 145 m/z, respectively, and for the 13C forms the target ions were 119, 132 and 147 m/z, respectively. For both forms of acetic and propionic acids the confirmative ion was 75 m/z. The confirmative ions for butyric acid were 75 and 117 m/z and for 13C butyric acid the confirmative ions were 75 and 119 m/z. Data were analyzed using the MassHunter Quantitative program (Agilent, USA), and the concentrations of each acid were calculated using internal standard methods with a seven-point matrix-matched linear calibration curve. Mean standard curve r2 values across all GC runs were 0.991 for acetic acid, 0.976 for propionic acid and 0.999 for butyric acid. Limits of detection were 0.27 μM, 0.24 μM and 0.04 μM for acetate, propionate, and butyrate, respectively. Limits of quantitation were 0.83 μM, 0.73 μM and 0.12 μM for acetate, propionate, and butyrate, respectively. Samples with concentrations below the limit of quantitation were excluded from analysis.

### Muscle glycogen

Frozen muscle samples were freeze-dried for 16 h at −55°C (Mechatech Systems, Bristol, UK), powdered using an agate pestle and mortar (Cole-Parmer, USA), aliquoted, and weighed. Samples were solubilized in 0.1 M NaOH and vortexed thoroughly, incubated at 80°C for 5 min using a dry thermostat, vortexed again and heated for another 5 min at 80°C. A 1:3 solution of 0.1 M HCl and citric acid buffer (0.2 M citric acid +0.2 M $Na_2HPO_4$, pH 5) was added to each sample and vortexed. Glycogen present in the supernatant was hydrolyzed using 200 mg•$mL^{-1}$ α-amyloglucosidase (~70 units•$mg^{-1}$) added to each sample. Samples were incubated for 1 h at room temperature on a slow tube rotator. Samples were centrifuged at 14000 x g for 5 min to pellet, and the supernatant was aliquoted to new tubes. Glucosyl units were determined using an enzymatic method adapted from Harris et al.,[90] with a 1:1 ratio of hexokinase and glucose-6-phosphate dehydrogenase (G-6-PDH; both 1 unit•$\mu L^{-1}$ with 2 μL each per sample). Concentrations of muscle glycogen were assessed in triplicate against a glucose standard curve after 20 min using a spectrophotometric plate reader (PHERAstar FS, BMG Labtech, USA).

### Muscle western blotting

Muscle samples were freeze-dried and powdered, and any blood or connective tissue was removed. Powdered samples were resuspended in radioimmunoprecipitation assay (50mM Tris-HCL pH 7.4, 150mM NaCL 0.5% Sodium deoxycholate, 0.1% SDS, and 0.1% NP-40; RIPA) buffer supplemented with protease inhibitors (Thermo Fisher) at a ratio of 100 μL $mg^{-1}$ and homogenized using a hand-held BioVortexer (BioSpec Products, USA). Samples were incubated for 1 h at 4°C with rotation, then centrifuged for 10 min at 20000 × g to remove insoluble material and the supernatant transferred to new 1.5 mL tubes and stored at −80°C. Protein content of each sample was determined using a BCA protein assay (Thermo Scientific, USA). Protein (50 μg per sample) was separated by SDS polyacrylamide gel electrophoresis (SDS-PAGE) on a 10% acrylamide gel over ~1 h at 200 V and transferred to a nitrocellulose membrane on a semi-dry transfer cell over 2.5 h at 150 mA. The membrane was blocked in a solution of 5% w/v skimmed milk powder and 0.1% Tween 20 in Tris-buffered saline (0.9%NaCL and 10mM Tris-HCL pH7.4), to make TBS-T for 30 min, washed thoroughly in TBS-T and incubated in primary antibody solution overnight at 4°C. Primary antibodies were diluted in 1% bovine serum albumin (BSA) in TBS-T with 0.02% sodium azide. Membranes were washed extensively in TBS-T and incubated in secondary antibody with rocking for 1 h at room temperature. Anti-rabbit IgG-HRP conjugate and anti-mouse IgG-HRP conjugate secondary antibodies were diluted 1:4000 in 5% milk powder (Marvel) TBS-T blocking solution. Membranes were washed extensively and developed using enhanced chemiluminescence (ECL) Prime or Select fluid according to the manufacturer's instructions (Cytiva, USA). Images were captured using an EPI Chemi Darkroom II (Ultra Violet Products Co., USA) and signals were quantified using Image Studio Lite software (v.5.2, LI-COR Biosciences, USA). Samples from the same participant were run on the same gel. Western blot data were normalized to β-actin as a housekeeper and expressed as fold-change from baseline for intra-group comparisons.

### Adipose tissue RNA extraction

Adipose tissue biopsy samples (100 g–400 g) were homogenized in 1 mL of QIAzol (Qiagen; Crawley, UK). Subsequently, 200 μL of chloroform was added and after vigorously mixing, the aqueous phase containing the total RNA was collected, mixed with 1.5 volume of ethanol before purifying the total RNA using a miRNeasy RNA extraction kit (Qiagen, Crawley, UK), following the manufacturer's instructions. Total RNA (250–500 ng) was reverse-transcribed using Applied Biosystems high-capacity cDNA reverse transcription kit (Applied Biosystems, Warrington, UK).

### Adipose tissue mRNA content by qPCR

Gene expression was analyzed by reverse transcription quantitative polymerase chain reaction (qPCR) using SYBR Green dye (Luna Universal qPCR Master Mix (New England Biolab, UK)). The qPCR analysis was performed on a StepOne qPCR machine (Applied Biosystems; Warrington, UK) using primers designed using Primer-BLAST NCBI (Primer designing tool (nih.gov)) and synthesized

by Merck, UK. Expression levels of the following genes was measured: GLUT 4, AKT1, AKT2, INSR, TBC1D1, TBC1D4, PDK4, PPARγ, GPR109/HCAR, CPT1A, IRS1, IRS2, SREBP1c, Leptin, Adiponectin, LPL, FAS, HSL, FABP4 (see Table S10 for primer sequences). Data obtained from the qPCR analysis were normalized using the internal calibrators 18s, Actin, and PPIA and differences in gene expression pre-to post-intervention were calculated using the ΔΔ comparative threshold (Ct) method. Prior to statistical analysis, any outliers were systematically removed from the dataset using the ROUT method with a significance level ($\alpha$) set at 1% in GraphPad software (Prism 10).

## QUANTIFICATION AND STATISTICAL ANALYSES

Sample size estimations were calculated using G*Power 3.1 software.[91] Using data from the Bath Breakfast Project,[16] the mean ± SD physical activity energy expenditure for the fasting *vs.* breakfast groups during the morning (when differences in carbohydrate availability between groups were present) were 311 ± 124 kcal *vs.* 492 ± 227 kcal (Cohen's d effect size = 0.998). A between-subject design with 20 participants in each group would provide an >85% chance (power) of detecting the expected effect with an $\alpha$-level of 0.05, so the aim was to recruit 60 participants.

Prism v9.5.0 (GraphPad Software Inc., USA) and SPSS v25 (IBM, USA) were used for statistical analyses. Data were checked for normal distribution by visual inspection of residual plots. As there was no clear indication of nonnormal distribution, the data were presented as means ± SD (and means ±95%CI). Total (tAUC) and incremental area under the curve (iAUC) were calculated using the Time Series Response Analyser.[92] Figures were drawn using Prism v9.5.0 (GraphPad Software Inc., USA). For all outcomes with quantitative units at week 4 and week 12, ANCOVA was used to assess differences between groups with baseline values as the covariate.[93] Unadjusted means are presented for baseline outcomes, but ANCOVA-adjusted means (and mean differences *vs.* MODSUG) are reported for week 4 and week 12 unless otherwise stated. Since skeletal muscle protein and adipose mRNA levels were expressed as the fold-change from baseline, one-way ANOVAs were used at week 4 and week 12 respectively to detect differences between groups. *Post-hoc* comparisons were made according to the principle of closed testing to assess the effect of sugar restriction (LOWSUG vs. MODSUG) or ketogenic carbohydrate restriction (LOWCHO vs. MODSUG).[94] Data from Visual Analogue Scales were assessed by repeated-measures ANOVA of within-group comparisons due to their subjective nature.[95] Figure legends state whether means or ANCOVA-adjusted means are presented for each outcome. Simple linear regression and Pearson correlation coefficients were used to assess linear associations between outcomes where appropriate. Significance was accepted at $p \leq 0.05$. Data are presented as mean and 95% confidence intervals (CI) unless otherwise stated.

Microbiome taxonomic composition data were analyzed at the phylum, genus and species levels and functional potential was analyzed using normalized pathway abundances not stratified by species. Output tables of raw reads were filtered to remove taxa that were not present in at least 5% of samples, and the PERFect permutational filtering R tool was then used to remove low-signal taxa.[96] Alpha diversity was calculated at the species level using the Shannon index to account for richness and evenness.[97] Beta diversity analysis was conducted at the species level based on Robust Aitchison distances to account for the sparse and compositional nature of microbiome data.[98] Nonmetric multidimensional scaling (NMDS) plots were used to visualize Beta diversity. Permutational multivariate analysis of variance (PERMANOVA) was used to test for differences in the Beta diversity of species and functional pathways between groups at each timepoint and between timepoints within each group, with permutations constrained to the level of participant where required to account for repeated measures sampling. Alpha and Beta diversity analyses were conducted using the vegan R package version 2.6–2.[99] Differential abundance of taxa and functional pathways from within and between each group was assessed using ALDEx2 and expressed as estimated effect size of change per taxon and per pathway.[100] Where multiple comparisons were made, $p$ values were adjusted using the Benjamini-Hochberg method and statistical significance was accepted at $q \leq 0.1$.

## ADDITIONAL RESOURCES

The study was registered at clinicaltrials.gov (NCT03574987).

Cell Reports Medicine, Volume *5*

## Supplemental information

# Ketogenic diet but not free-sugar restriction alters glucose tolerance, lipid metabolism, peripheral tissue phenotype, and gut microbiome: RCT

**Aaron Hengist, Russell G. Davies, Jean-Philippe Walhin, Jariya Buniam, Lucy H. Merrell, Lucy Rogers, Louise Bradshaw, Alfonso Moreno-Cabañas, Peter J. Rogers, Jeff M. Brunstrom, Leanne Hodson, Luc J.C. van Loon, Wiley Barton, Ciara O'Donovan, Fiona Crispie, Orla O'Sullivan, Paul D. Cotter, Kathryn Proctor, James A. Betts, Françoise Koumanov, Dylan Thompson, and Javier T. Gonzalez**

**SUPPLEMENTAL INFORMATION**

**Title:** Ketogenic diet (but not free-sugar restriction) alters glucose tolerance, lipid metabolism, peripheral tissue phenotype and gut microbiome: RCT.

**Author list:** Aaron Hengist*[#a], Russell G Davies[#a], Jean-Philippe Walhin[a], Jariya Buniam[a,b], Lucy H Merrell[a], Lucy Rogers[a], Louise Bradshaw[a], Alfonso Moreno-Cabañas[a], Peter J Rogers[c], Jeff M Brunstrom[c], Leanne Hodson[d], Luc JC van Loon[e], Wiley Barton[f,g,h], Ciara O'Donovan[f,g], Fiona Crispie[f,g], Orla O'Sullivan[f,g,h], Paul D Cotter[f,g,h], Kathryn Proctor[a], James A Betts[a], Françoise Koumanov[a], Dylan Thompson[a], Javier T Gonzalez*[a]

This document contains 6 supplemental figures and 9 supplemental tables.

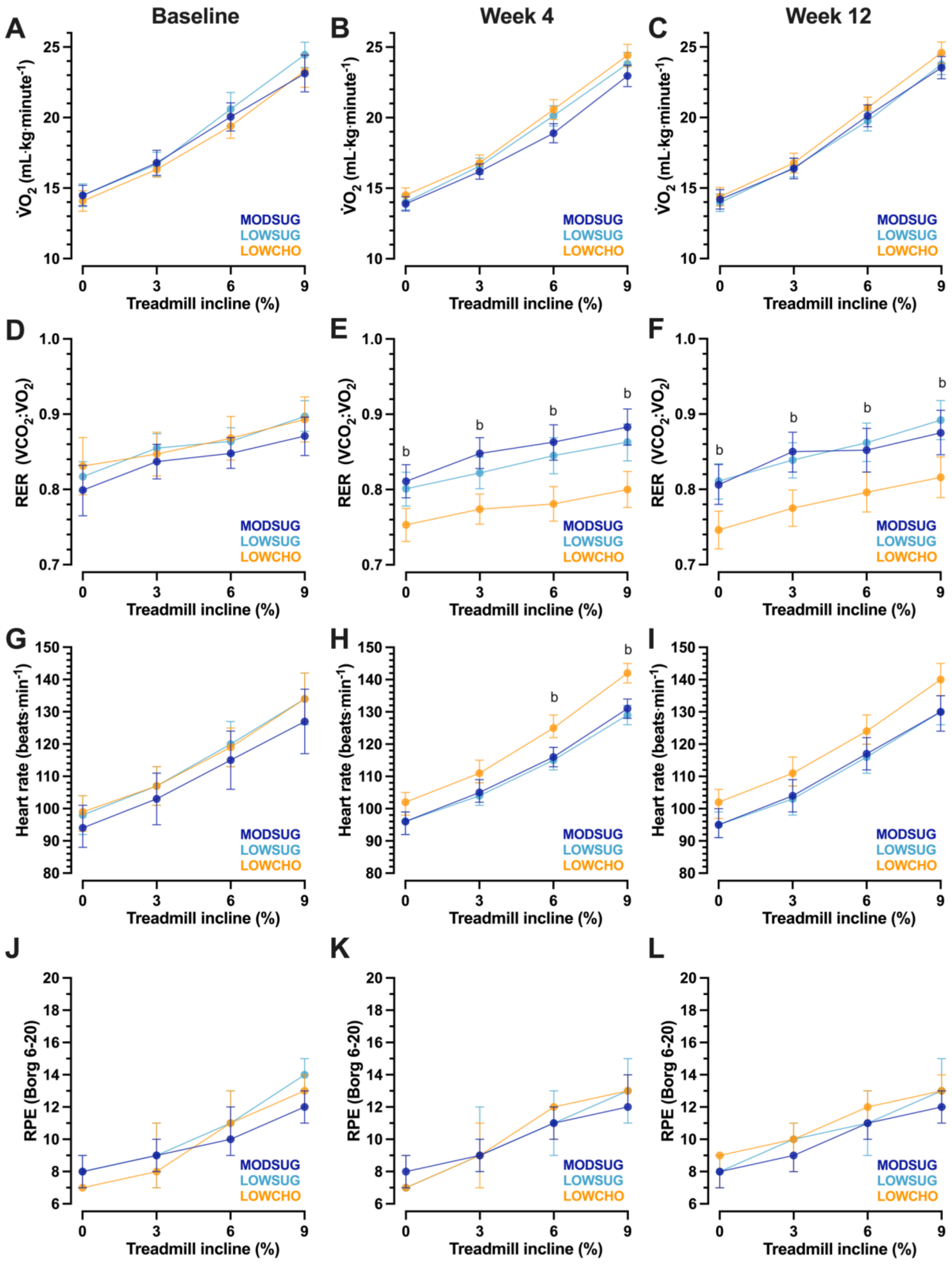


**Figure S1. Physiological responses during incremental exercise tests at baseline and following 4 weeks and 12 weeks of moderate-sugar diet (MODSUG), low-sugar diet (LOWSUG), or low-carbohydrate diet (LOWCHO) in healthy men and women. Related to Figure 1.** Relative $\dot{V}O_2$ (**A-C**), respiratory exchange ratio (RER) (**D-F**), heart rate (**G-I**), and rate of perceived exertion (RPE) (**J-L**) during exercise at 5.2 km·h$^{-1}$ and incremental inclines from 0 to 9%. Data shown are mean (95% CI) for Baseline and ANCOVA-adjusted mean (95% CI) for Week 4 and Week 12. Week 4 n=52, Week 12 n=45. $^{b}p \leq 0.05$ MODSUG *vs* LOWCHO.

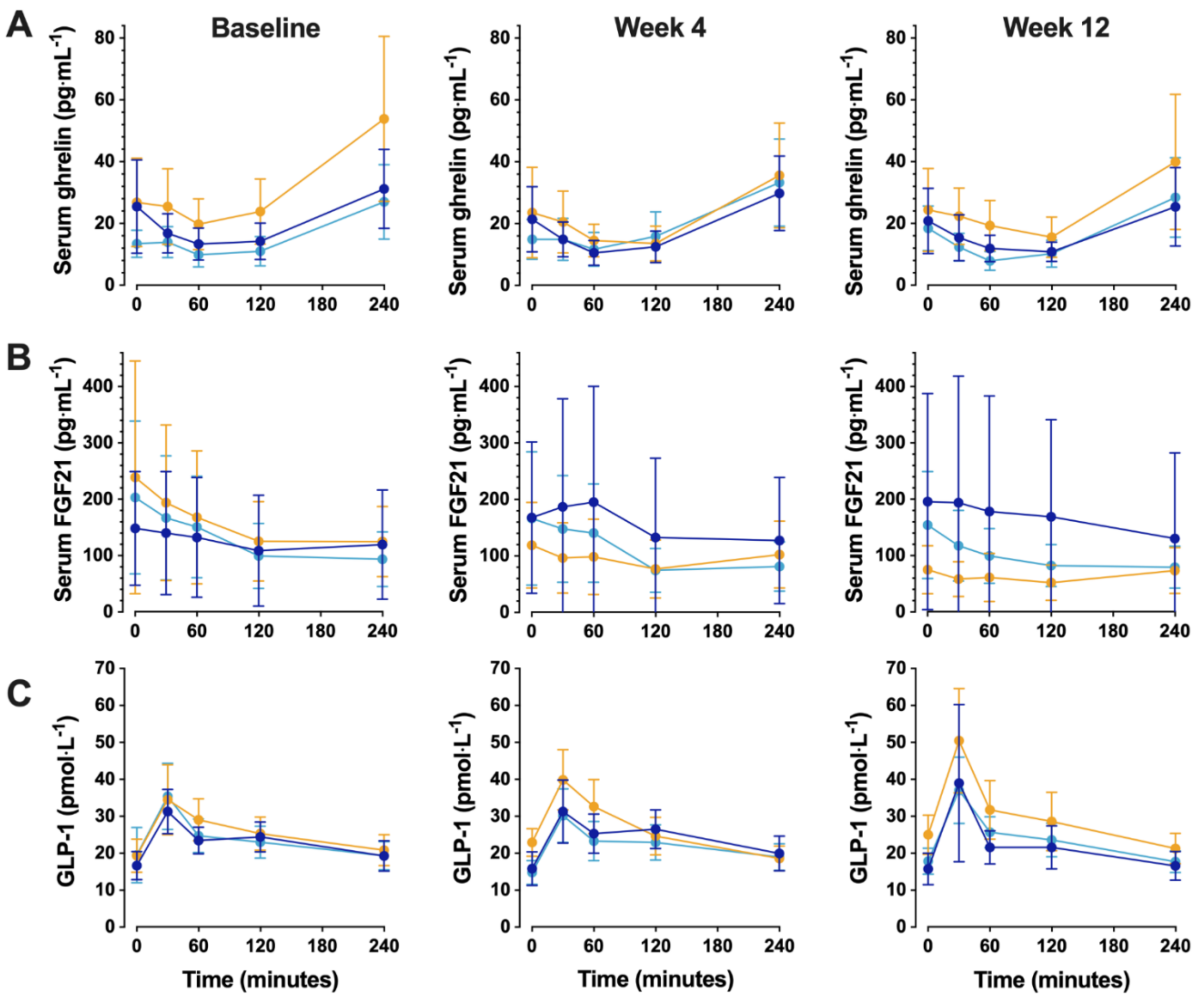


**Figure S2. Serum ghrelin, fibroblast growth factor 21 (FGF21), and glucagon-like peptide-1 (GLP-1) responses to mixed meal tolerance tests (mean [range] 502 [331 to 715 kcal]; 54% carbohydrate [23% of which sugars], 31% fat, 15% protein) at baseline, week 4, and week 12 of moderate sugar (MODSUG), free-sugar restriction (LOWSUG), or ketogenic carbohydrate restricted (LOWCHO) diets. Related to Figure 7.** Unadjusted postprandial concentrations of serum ghrelin (**A**), FGF21 (**B**) GLP-1 (**C**). Week 4 n=43-50; week 12 n=38-42.

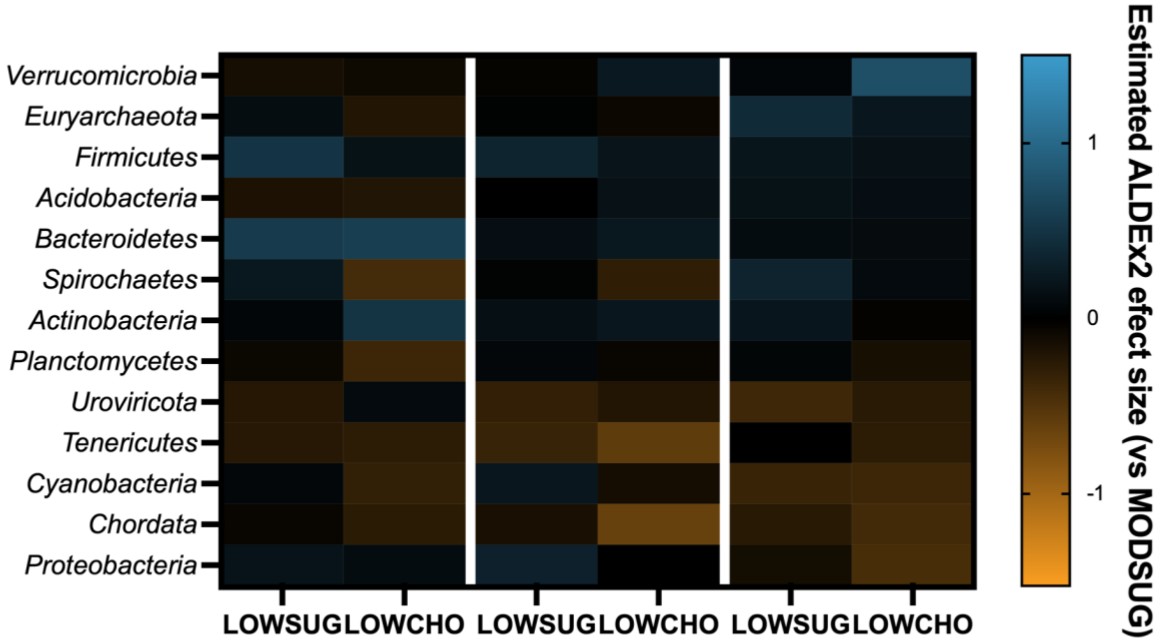


**Figure S3. Differences in relative abundance of the gut microbiome at the phylum level at baseline, week 4, and week 12 free-sugar restriction (LOWSUG), or ketogenic carbohydrate restricted (LOWCHO) diets compared with moderate sugar (MODSUG) Related to Figure 5.** Estimated effect size of change in center log ratio abundance of gut microbiome phyla vs MODSUG. Week 4 *n*=48, week 12 *n*=41.

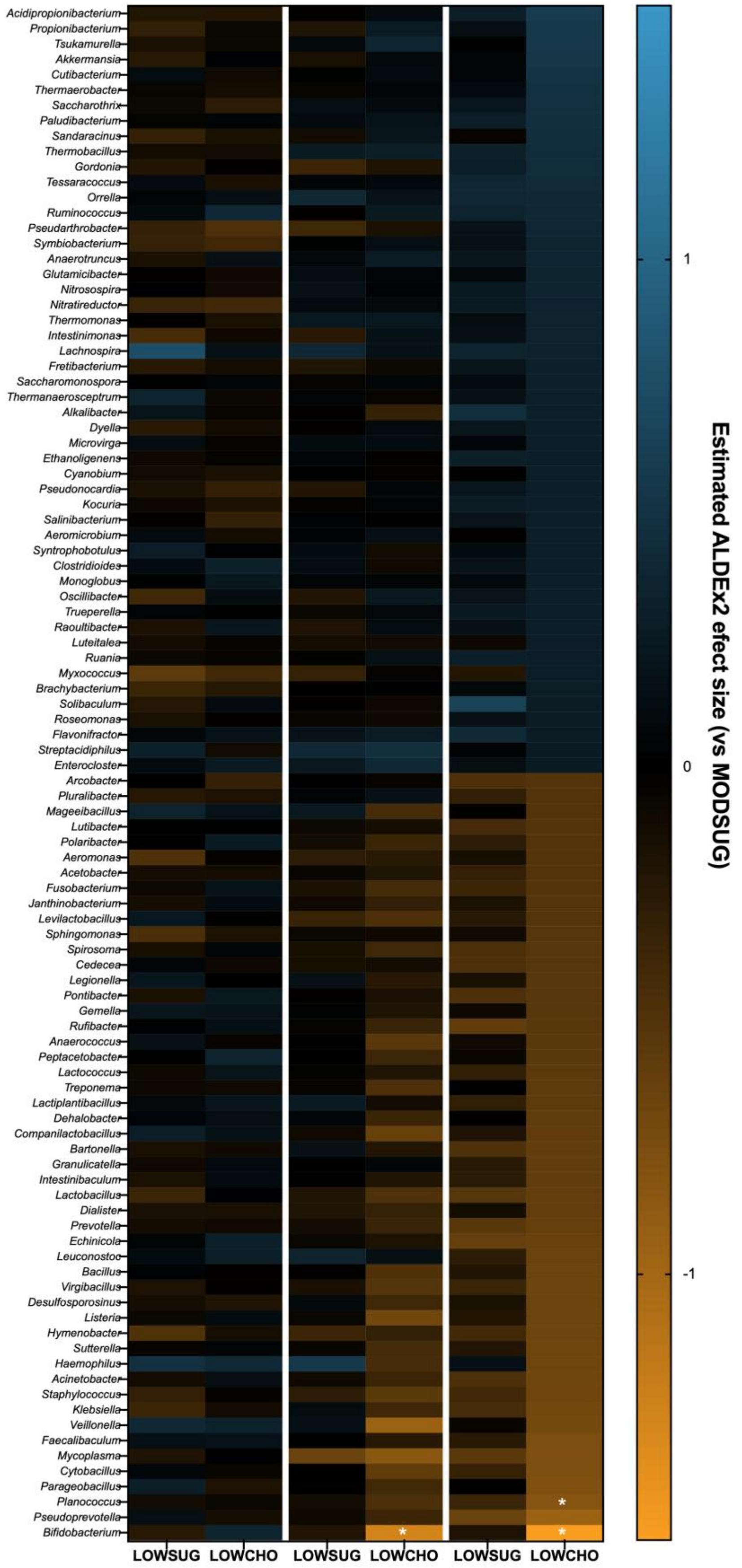


**Figure S4. Differences in relative abundance of the gut microbiome at the genus level at baseline (left-hand), week 4 (center), and week 12 (right-hand) with free-sugar restriction (LOWSUG), or ketogenic carbohydrate restricted (LOWCHO) diets compared with moderate sugar (MODSUG). Related to Figure 5.** Fifty largest increases and 50 largest decreases in estimated effect size of change in center log ratio abundance vs MODSUG are presented. Week 4 *n*=48, week 12 *n*=41.

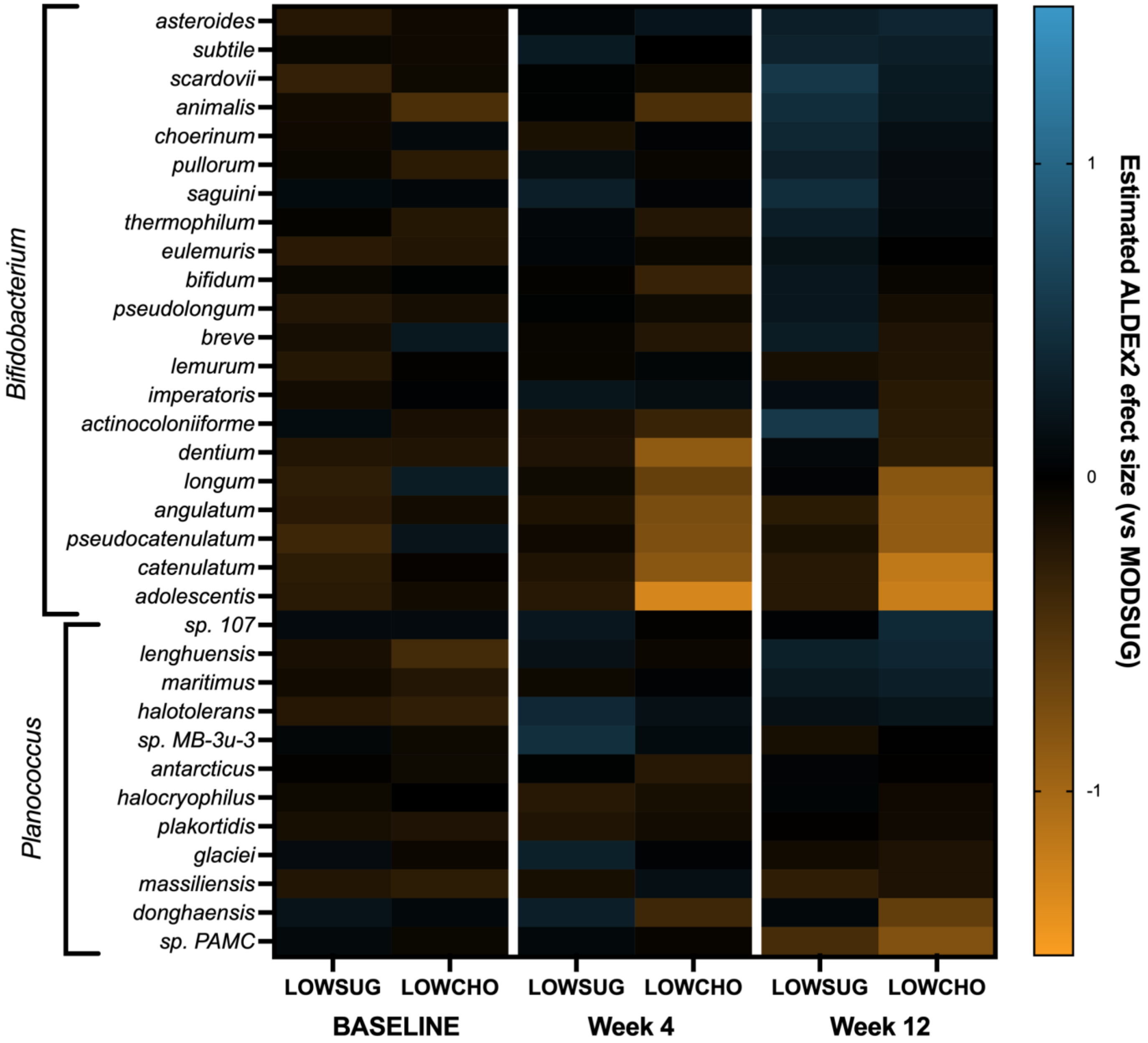


**Figure S5. Differences in relative abundance of the gut microbiome at the species level for *Bifidobacterium* and *Planoccocus* at baseline (left-hand), week 4 (center), and week 12 (right-hand) with free-sugar restriction (LOWSUG), or ketogenic carbohydrate restricted (LOWCHO) diets compared with moderate sugar (MODSUG). Related to Figure 5.** Estimated effect size of change in center log ratio abundance of gut microbiome species vs MODSUG. Week 4 *n*=48, week 12 *n*=41.

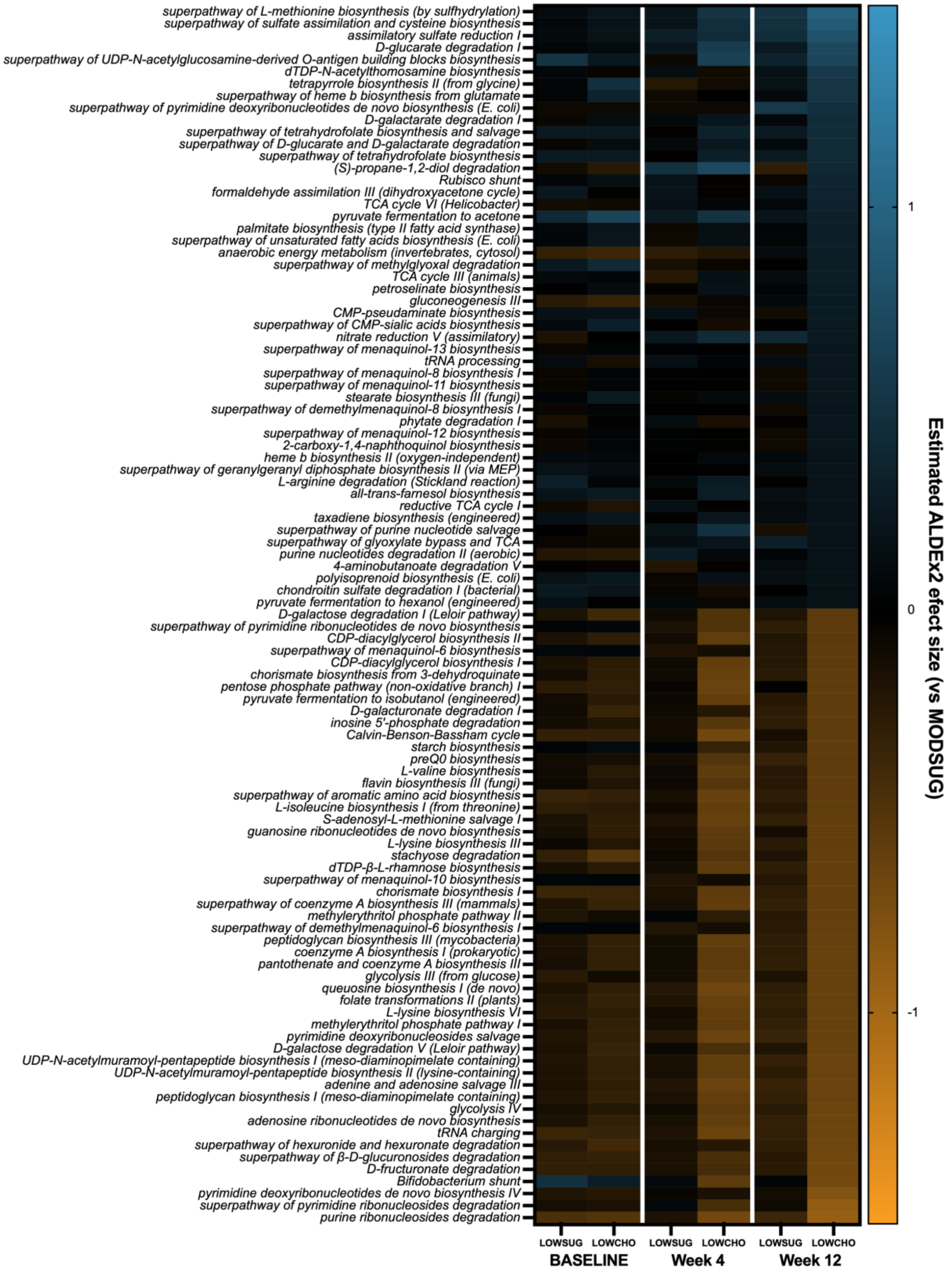


**Figure S6. Differences in HUMANn2 metabolic pathways abundance at baseline (left-hand), week 4 (center), and week 12 (right-hand) with free-sugar restriction (LOWSUG), or ketogenic carbohydrate restricted (LOWCHO) diets compared with moderate sugar (MODSUG). Related to Figure 5.** Fifty largest increases and 50 largest decreases in estimated effect size of change in center log ratio abundance vs MODSUG are presented. Week 4 *n*=48, week 12 *n*=41.

**Table S1. Baseline characteristics for participants following a moderate-sugar control (MODSUG), low-sugar (LOWSUG), or low-carbohydrate diets (LOWCHO). Related to Figure 1.**

| | Week 4 completers | | | Week 12 completers | | |
|---|---|---|---|---|---|---|
| | **MODSUG** | **LOWSUG** | **LOWCHO** | **MODSUG** | **LOWSUG** | **LOWCHO** |
| *n* (female, male) | 18 (11, 7) | 17 (10, 7) | 18 (9, 9) | 13 (8, 5) | 16 (10, 6) | 16 (7, 9) |
| Age (years) | 39 ± 17 | 31 ± 16 | 33 ± 14 | 41 ± 16 | 32 ± 16 | 34 ± 14 |
| Height (m) | 1.72 ± 0.1 | 1.71 ± 0.05 | 1.71 ± 0.1 | 1.72 ± 0.11 | 1.71 ± 0.06 | 1.72 ± 0.1 |
| Body mass (kg) | 72.1 ± 10.8 | 69.4 ± 7 | 73.4 ± 13.4 | 73.3 ± 11.3 | 69.4 ± 7.2 | 75.5 ± 12.8 |
| BMI ($kg·m^{-2}$) | 24.1 ± 2.7 | 23.5 ± 2 | 24.8 ± 3 | 24.6 ± 2.8 | 23.6 ± 2.1 | 25.2 ± 2.9 |
| Waist circumference (cm) | 82 ± 9.2 | 79.3 ± 6.1 | 83.9 ± 9.3 | 83.3 ± 10.3 | 79 ± 6.1 | 85.8 ± 8 |
| Hip circumference (cm) | 101.2 ± 5.4 | 99.4 ± 5.5 | 101.5 ± 6.9 | 101.8 ± 5.4 | 99.5 ± 5.7 | 102.4 ± 6.7 |
| Waist: hip (ratio) | 0.8 ± 0.05 | 0.79 ± 0.04 | 0.82 ± 0.07 | 0.81 ± 0.06 | 0.79 ± 0.04 | 0.83 ± 0.06 |
| Fat free mass (kg) | 52 ± 12 | 51 ± 7 | 51 ± 12 | 53 ± 14 | 50 ± 7 | 53 ± 12 |
| Fat mass (kg) | 18 ± 7 | 17 ± 7 | 20 ± 7 | 18 ± 8 | 17 ± 7 | 20 ± 7 |
| Android fat mass (g) | 1387 ± 900 | 1275 ± 746 | 1677 ± 797 | 1477 ± 1008 | 1267 ± 770 | 1769 ± 799 |
| Gynoid fat mass (g) | 3216 ± 1168 | 3119 ± 1259 | 3625 ± 1243 | 3216 ± 1135 | 3160 ± 1289 | 3641 ± 1314 |
| Bone mineral density ($g·cm^{-2}$) | 1.13 ± 0.11 | 1.15 ± 0.06 | 1.16 ± 0.12 | 1.15 ± 0.13 | 1.15 ± 0.06 | 1.17 ± 0.12 |
| Systolic blood pressure (mmHg) | 119 ± 11 | 121 ± 7 | 117 ± 10 | 120 ± 11 | 121 ± 8 | 116 ± 10 |
| Diastolic blood pressure (mmHg) | 77 ± 6 | 77 ± 6 | 76 ± 5 | 79 ± 6 | 77 ± 7 | 75 ± 5 |
| **Energy intake** | | | | | | |
| Starch ($g·d^{-1}$) | 155 ± 74 | 161 ± 75 | 123 ± 59 | 155 ± 83 | 160 ± 77 | 123 ± 59 |
| Fruit and vegetable sugars ($g·d^{-1}$) | 19 ± 13 | 19 ± 13 | 16 ± 9 | 20 ± 13 | 20 ± 13 | 18 ± 9 |
| Milk sugars ($g·d^{-1}$) | 5 ± 6 | 4 ± 4 | 3 ± 5 | 2 ± 3 | 4 ± 4 | 2 ± 6 |
| Liquid free sugars ($g·d^{-1}$) | 19 ± 26 | 14 ± 17 | 10 ± 9 | 20 ± 31 | 14 ± 18 | 10 ± 10 |
| Solid free sugars ($g·d^{-1}$) | 52 ± 24 | 55 ± 29 | 44 ± 17 | 48 ± 24 | 57 ± 29 | 44 ± 13 |
| Fat ($g·d^{-1}$) | 93 ± 36 | 82 ± 17 | 84 ± 27 | 93 ± 40 | 82 ± 18 | 87 ± 26 |
| Protein ($g·d^{-1}$) | 91 ± 42 | 87 ± 24 | 89 ± 45 | 93 ± 49 | 87 ± 25 | 94 ± 45 |
| Ethanol ($g·d^{-1}$) | 19 ± 18 | 10 ± 10 | 10 ± 12 | 18 ± 20 | 9 ± 10 | 10 ± 12 |
| Fiber ($g·d^{-1}$) | 25 ± 9 | 26 ± 14 | 21 ± 8 | 26 ± 10 | 27 ± 14 | 23 ± 7 |
| **Energy expenditure** | | | | | | |
| PAEE ($kcal·d^{-1}$) | 1368 ± 677 | 1285 ± 515 | 1200 ± 674 | 1255 ± 716 | 1200 ± 399 | 1286 ± 733 |
| Step count ($steps·d^{-1}$) | 9609 ± 4939 | 10485 ± 3680 | 8951 ± 5430 | 9983 ± 4976 | 10125 ± 3810 | 8951 ± 5430 |
| RMR ($kcal·d^{-1}$) | 1671 ± 353 | 1637 ± 247 | 1651 ± 365 | 1665 ± 368 | 1634 ± 255 | 1694 ± 386 |
| Protein oxidation ($g·min^{-1}$) | 0.02 ± 0.01 | 0.02 ± 0 | 0.02 ± 0.01 | 0.03 ± 0.01 | 0.02 ± 0 | 0.03 ± 0.01 |
| Sleeping heart rate ($beats·min^{-1}$) | 49 ± 6 | 52 ± 8 | 52 ± 5 | 50 ± 6 | 53 ± 9 | 53 ± 4 |

Data are means ± SD.

**Table S2. Baseline circulating biochemistry and muscle glycogen concentrations for participants following moderate-sugar control (MODSUG), low-sugar (LOWSUG), or low-carbohydrate diets (LOWCHO). Related to Figure 4.**

| | Week 4 completers | | | Week 12 completers | | |
|---|---|---|---|---|---|---|
| | **MODSUG** | **LOWSUG** | **LOWCHO** | **MODSUG** | **LOWSUG** | **LOWCHO** |
| **Fasting** | | | | | | |
| Total cholesterol (mmol·L$^{-1}$) | 4.77 ± 1.36 | 4.48 ± 1.19 | 4.52 ± 0.86 | 4.75 ± 1.62 | 4.65 ± 1.17 | 4.45 ± 0.85 |
| HDL-C (mmol·L$^{-1}$) | 1.36 ± 0.42 | 1.4 ± 0.37 | 1.29 ± 0.25 | 1.27 ± 0.32 | 1.47 ± 0.37 | 1.26 ± 0.25 |
| LDL-C (mmol·L$^{-1}$) | 2.7 ± 1.26 | 2.56 ± 1.04 | 2.49 ± 0.72 | 2.68 ± 1.44 | 2.62 ± 1.02 | 2.46 ± 0.68 |
| ApoB (mg·dL$^{-1}$) | 82 ± 26 | 71 ± 24 | 73 ± 29 | 81 ± 30 | 72 ± 23 | 72 ± 27 |
| Albumin (g·L$^{-1}$) | 41 ± 3 | 42 ± 3 | 42 ± 2 | 41 ± 3 | 42 ± 3 | 42 ± 2 |
| WBC count (n·10$^{9}$·L$^{-1}$) | 4.9 ± 1.4 | 5.3 ± 1.3 | 8.1 ± 11.2 | 4.7 ± 1.5 | 5.3 ± 1.3 | 5.4 ± 1.2 |
| RBC count (n·10$^{12}$·L$^{-1}$) | 4.6 ± 0.4 | 4.4 ± 0.4 | 4.5 ± 0.4 | 4.5 ± 0.3 | 4.4 ± 0.4 | 4.5 ± 0.4 |
| Hemoglobin (g·dL$^{-1}$) | 13.9 ± 1.1 | 13.3 ± 1.3 | 13.4 ± 1.1 | 13.9 ± 0.9 | 13.3 ± 1.4 | 13.5 ± 1.1 |
| Hematocrit (%) | 39 ± 2 | 39 ± 3 | 39 ± 2 | 39 ± 2 | 38 ± 3 | 39 ± 2 |
| Platelet count (n·10$^{9}$·L$^{-1}$) | 220 ± 49 | 229 ± 58 | 212 ± 48 | 211 ± 49 | 219 ± 43 | 207 ± 45 |
| Leptin (ng·mL$^{-1}$) | 12 ± 11 | 14 ± 13 | 14 ± 14 | 13 ± 13 | 14 ± 13 | 14 ± 14 |
| Ghrelin (pg·mL$^{-1}$) | 27 ± 25 | 13 ± 8 | 27 ± 27 | 18 ± 15 | 12 ± 7 | 27 ± 28 |
| FGF21 (pg·mL$^{-1}$) | 155 ± 185 | 189 ± 265 | 247 ± 385 | 188 ± 212 | 204 ± 272 | 420 ± 1099 |
| GLP-1 (pg·mL$^{-1}$) | 16 ± 7 | 19 ± 14 | 20 ± 8 | 13 ± 4 | 20 ± 14 | 20 ± 9 |
| Acetate (μmol·L$^{-1}$) | 36 ± 33 | 27 ± 14 | 30 ± 15 | 32 ± 23 | 28 ± 17 | 30 ± 16 |
| Propionate (μmol·L$^{-1}$) | 3.5 ± 2.3 | 3 ± 1.9 | 2.2 ± 1.2 | 3.2 ± 1.2 | 3.1 ± 0.5 | 1.6 ± 0.5 |
| Butyrate (μmol·L$^{-1}$) | 0.55 ± 0.37 | 0.6 ± 0.43 | 0.51 ± 0.26 | 0.55 ± 0.37 | 0.54 ± 0.36 | 0.51 ± 0.28 |
| CRP (mg·L$^{-1}$) | 0.83 ± 0.74 | 1.03 ± 1.11 | 0.91 ± 0.87 | 1.05 ± 0.85 | 0.95 ± 1.07 | 0.98 ± 0.92 |
| LBP (mg·L$^{-1}$) | 11.2 ± 5.5 | 18 ± 8.4 | 15.3 ± 12.7 | 10.8 ± 6.1 | 17.7 ± 8.5 | 14.8 ± 13.2 |
| **Postprandial** | | | | | | |
| Glucose iAUC (mmol·L$^{-1}$·240 min) | 188 ± 75 | 179 ± 156 | 195 ± 118 | 191 ± 84 | 177 ± 157 | 200 ± 119 |
| Insulin iAUC (nmol·L$^{-1}$·240 min) | 31 ± 22 | 36 ± 16 | 34 ± 18 | 32 ± 25 | 35 ± 17 | 34 ± 18 |
| C-peptide iAUC (ng·mL$^{-1}$·240 min) | 253 ± 161 | 259 ± 98 | 314 ± 129 | 271 ± 155 | 240 ± 97 | 330 ± 124 |
| Lactate iAUC (mmol·L$^{-1}$·240 min) | 66 ± 25 | 67 ± 30 | 61 ± 29 | 65 ± 23 | 68 ± 31 | 58 ± 23 |
| βOHB AUC (mmol·L$^{-1}$·240 min) | 18 ± 5 | 21 ± 10 | 20 ± 8 | 17 ± 3 | 21 ± 10 | 20 ± 8 |
| TAG iAUC (mmol·L$^{-1}$·240 min) | 53 ± 60 | 31 ± 23 | 42 ± 34 | 56 ± 65 | 30 ± 23 | 46 ± 35 |
| NEFA AUC (mmol·L$^{-1}$·240 min) | 56 ± 15 | 56 ± 18 | 57 ± 24 | 56 ± 15 | 57 ± 18 | 57 ± 25 |
| Glycerol AUC (mmol·L$^{-1}$·240 min) | 11 ± 9 | 8 ± 7 | 10 ± 8 | 8 ± 7 | 8 ± 7 | 10 ± 8 |
| Ghrelin AUC (pg·mL$^{-1}$·240 min) | 4920 ± 2979 | 3755 ± 2666 | 7762 ± 6611 | 3993 ± 1714 | 3357 ± 2350 | 8271 ± 6763 |
| FGF21 AUC (pg·mL$^{-1}$·240 min) | 29 ± 45 | 27 ± 33 | 36 ± 41 | 36 ± 53 | 29 ± 34 | 58 ± 125 |
| iAUC GLP-1 (pg·mL$^{-1}$·240 min) | 1609 ± 872 | 1450 ± 1206 | 1558 ± 1462 | 1763 ± 940 | 1422 ± 1190 | 1577 ± 1478 |

| | | | | | | |
|---|---|---|---|---|---|---|
| **CGMS** | | | | | | |
| Mean glucose (mmol·L$^{-1}$) | 4.8 ± 0.5 | 4.7 ± 0.6 | 4.6 ± 0.3 | 5 ± 0.3 | 4.5 ± 0.4 | 4.6 ± 0.3 |
| Glucose CV (%) | 10.9 ± 3.4 | 11.6 ± 2.4 | 12.3 ± 5.3 | 11 ± 3.9 | 11.2 ± 2.8 | 11.6 ± 5.4 |
| Glucose SD (mmol·L$^{-1}$) | 0.5 ± 0.1 | 0.5 ± 0 | 0.5 ± 0.2 | 0.5 ± 0.1 | 0.5 ± 0.1 | 0.5 ± 0.2 |
| **Skeletal muscle** | | | | | | |
| Glycogen (mmol·kg dm$^{-1}$) | 278 ± 78 | 254 ± 140 | 276 ± 77 | 265 ± 76 | 220 ± 155 | 296 ± 76 |

Data are means ± SD.

**Table S3. Self-reported daily nutrient intake during 12 weeks of moderate-sugar diet (MODSUG), low-sugar diet (LOWSUG), or low-carbohydrate diet (LOWCHO). Related to Figure 1.**

| | Weeks 1 to 4 | | | Weeks 5 to 12 | | |
|---|---|---|---|---|---|---|
| | **MODSUG** | **LOWSUG** | **LOWCHO** | **MODSUG** | **LOWSUG** | **LOWCHO** |
| Starch ($g \cdot d^{-1}$) | 151 ± 68 | 153 ± 60 | 11 ± 5 | 144 ± 65 | 160 ± 64 | 15 ± 6 |
| Fruit and vegetable sugars ($g \cdot d^{-1}$) | 17 ± 12 | 19 ± 15 | 6 ± 2 | 17 ± 12 | 19 ± 18 | 6 ± 2 |
| Milk sugars ($g \cdot d^{-1}$) | 4 ± 4 | 3 ± 3 | 0 ± 2 | 3 ± 4 | 4 ± 4 | 0 ± 1 |
| Liquid free sugars ($g \cdot d^{-1}$) | 33 ± 23 | 2 ± 3 | 0 ± 1 | 35 ± 22 | 2 ± 4 | 1 ± 1 |
| Solid free sugars ($g \cdot d^{-1}$) | 67 ± 23 | 17 ± 6 | 8 ± 2 | 64 ± 29 | 19 ± 7 | 11 ± 4 |
| Fat ($g \cdot d^{-1}$) | 94 ± 37 | 87 ± 18 | 135 ± 51 | 89 ± 40 | 80 ± 15 | 145 ± 54 |
| Protein ($g \cdot d^{-1}$) | 91 ± 42 | 86 ± 22 | 96 ± 32 | 86 ± 32 | 84 ± 24 | 97 ± 39 |
| Ethanol ($g \cdot d^{-1}$) | 10 ± 8 | 7 ± 9 | 5 ± 8 | 11 ± 9 | 7 ± 8 | 4 ± 6 |
| Fiber ($g \cdot d^{-1}$) | 26 ± 9 | 26 ± 11 | 14 ± 5 | 23 ± 9 | 26 ± 11 | 15 ± 6 |

Data are means ± SD.

**Table S4. Resting metabolic rate, protein oxidation, sleeping heart rate, fasting blood pressure and daily step count at week 4, and week 12 of moderate-sugar diet (MODSUG), low-sugar diet (LOWSUG), or low-carbohydrate diet (LOWCHO) in healthy men and women. Related to Figure 1.**

| | Week 4 | | | Week 12 | | |
|---|---|---|---|---|---|---|
| | MODSUG | LOWSUG | LOWCHO | MODSUG | LOWSUG | LOWCHO |
| Resting metabolic rate ($g \cdot min^{-1}$) | 1658 (1597 to 1719) | 1674 (1611 to 1737) | 1609 (1548 to 1670) | 1653 (1498 to 1807) | 1594 (1450 to 1738) | 1685 (1545 to 1824) |
| Resting metabolic $rate_{adjFFM}$ ($g \cdot min^{-1}$) | 1653 (1591 to 1715) | 1669 (1605 to 1732) | 1620 (1555 to 1684) | 1641 (1484 to 1799) | 1614 (1456 to 1772) | 1687 (1543 to 1831) |
| Protein oxidation ($g \cdot min^{-1}$) | 0.025 (0.018 to 0.032) | 0.032 (0.025 to 0.038) | 0.033 (0.027 to 0.040) | 0.030 (0.019 to 0.041) | 0.028 (0.019 to 0.037) | 0.036 (0.027 to 0.045) |
| Sleeping heart rate ($beats \cdot min^{-1}$) | 52 (51 to 54) | 51 (49 to 52) | 53 (51 to 55) | 53 (51 to 55) | 51 (50 to 53) | 55[b] (54 to 57) |
| Systolic blood pressure (mmHg) | 117 (114 to 120) | 115 (112 to 117) | 115 (112 to 118) | 116 (112 to 120) | 116 (113 to 120) | 119 (116 to 123) |
| Diastolic blood pressure (mmHg) | 75 (73 to 78) | 76 (73 to 78) | 75 (73 to 78) | 76 (74 to 79) | 76 (74 to 79) | 76 (74 to 78) |
| Step count ($steps \cdot d^{-1}$) | 8589 (7102 to 10075) | 8835 (7341 to 10329) | 9330 (7717 to 10942) | 8634 (7324 to 9943) | 8242 (6931 to 9552) | 9109 (7640 to 10579) |

Data are mean (95% CI) from ANCOVA with baseline values as the covariate ($_{adjFFM}$ indicates the change in FFM also added as a covariate). Step count for week 4 reflect the average over weeks 1 to 4 and for week 12 reflect the average over weeks 5 to 12.

**Table S5. Body composition following 4 weeks and 12 weeks of moderate-sugar diet (MODSUG), low-sugar diet (LOWSUG), or low-carbohydrate diet (LOWCHO) in healthy men and women. Related to Figure 2.**

| | Week 4 | | | Week 12 | | |
|---|---|---|---|---|---|---|
| | MODSUG | LOWSUG | LOWCHO | MODSUG | LOWSUG | LOWCHO |
| Waist:hip (ratio) | 0.81 (0.80 to 0.82) | 0.81 (0.80 to 0.82) | 0.79 (0.78 to 0.80) | 0.82 (0.80 to 0.84) | 0.82 (0.81 to 0.84) | 0.80 (0.79 to 0.82) |
| Fat free mass (kg) | 51.8 (51.3 to 52.4) | 51.8 (51.3 to 52.4) | 50.7[b] (50.3 to 51.4) | 52.0 (51.2 to 52.9) | 52.6 (51.8 to 53.4) | 51.4 (50.7 to 52.2) |
| Fat mass (kg) | 18.8 (18.4 to 19.2) | 17.8[a] (17.4 to 18.3) | 17.2[b] (16.8 to 17.6) | 19.5 (18.6 to 20.3) | 17.3[a] (16.6 to 18.1) | 16.6[b] (15.8 to 17.3) |
| Android fat mass (g) | 1455 (1387 to 1526) | 1335[a] (1268 to 1402) | 1312[b] (1247 to 1377) | 1593 (1473 to 1714) | 1337[a] (1235 to 1438) | 1269[b] (1168 to 1371) |
| Gynoid fat mass (g) | 3296 (3205 to 3387) | 3170 (3082 to 3259) | 3085[b] (2998 to 3172) | 3427 (3266 to 3588) | 3001[a] (2867 to 3134) | 2963[b] (2828 to 3098) |
| Bone mineral density ($g \cdot cm^{-2}$) | 1.15 (1.14 to 1.16) | 1.15 (1.14 to 1.16) | 1.15 (1.14 to 1.16) | 1.18 (1.16 to 1.20) | 1.16 (1.14 to 1.18) | 1.16 (1.15 to 1.18) |

Data shown are ANCOVA-adjusted mean (95% CI) for Week 4 and Week 12 (with baseline values as the covariate). [a]$p < 0.05$ LOWSUG vs MODSUG, [b]$p < 0.05$ LOWCHO vs MODSUG. Week 4 n=53, Week 12 n=45. DXA-derived measures of tissue mass and bone density.

**Table S6. Fasting circulating biochemical and hematology markers following 4 weeks and 12 weeks of moderate-sugar diet (MODSUG), low-sugar diet (LOWSUG), or low-carbohydrate diet (LOWCHO) in healthy men and women. Related to Figure 3.**

| | Week 4 | | | Week 12 | | |
|---|---|---|---|---|---|---|
| | MODSUG | LOWSUG | LOWCHO | MODSUG | LOWSUG | LOWCHO |
| Fasting glucose ($mmol \cdot L^{-1}$) | 5.44 (5.21 to 5.68) | 5.20 (4.96 to 5.45) | 4.98[b] (4.74 to 5.21) | 5.83 (5.49 to 6.18) | 5.39 (5.11 to 5.68) | 5.51 (5.22 to 5.79) |
| Fasting insulin ($pmol \cdot L^{-1}$) | 29 (24 to 34) | 28 (23 to 34) | 39 (24 to 34) | 33 (26 to 40) | 25 (20 to 31) | 27 (21 to 32) |
| Fasting c-peptide ($pg \cdot mL^{-1}$) | 549 (450 to 649) | 600 (497 to 702) | 496 (395 to 596) | 564 (445 to 683) | 596 (496 to 695) | 538 (438 to 638) |
| Fasting lactate ($mmol \cdot L^{-1}$) | 1.00 (0.87 to 1.12) | 0.99 (0.86 to 1.12) | 0.94 (0.81 to 1.06) | 1.03 (0.87 to 1.19) | 0.88 (0.75 to 1.02) | 0.90 (0.77 to 1.04) |
| Fasting βOHB ($mmol \cdot L^{-1}$) | 0.12 (0.00 to 0.39) | 0.23 (0.00 to 0.50) | 1.28[b] (1.01 to 1.54) | 0.14 (0.00 to 0.37) | 0.21 (0.02 to 0.40) | 0.82[b] (0.62 to 1.01) |
| Fasting FGF21 ($pg \cdot mL^{-1}$) | 196 (116 to 277) | 170 (87 to 253) | 97 (14 to 180) | 196 (139 to 254) | 138 (90 to 185) | 94[b] (44 to 143) |
| Fasting ghrelin ($pg \cdot mL^{-1}$) | 18.2 (14.1 to 22.2) | 22.1 (17.8 to 26.4) | 19.4 (15.5 to 23.4) | 21.7 (17.7 to 25.6) | 24.4 (20.9 to 27.8) | 18.0 (14.7 to 21.4) |
| Fasting albumin ($g \cdot L^{-1}$) | 41.6 (40.4 to 42.8) | 41.9 (40.7 to 43.1) | 41.7 (40.5 to 42.8) | 43.0 (40.5 to 45.4) | 42.1 (40.1 to 44.1) | 43.0 (40.9 to 45.0) |
| Fasting TAG ($mmol \cdot L^{-1}$) | 0.74 (0.59 to 0.88) | 0.74 (0.59 to 0.89) | 0.90 (0.76 to 1.05) | 0.68 (0.43 to 0.92) | 0.71 (0.52 to 0.90) | 0.93 (0.74 to 1.12) |
| Fasting NEFA ($mmol \cdot L^{-1}$) | 0.49 (0.40 to 0.59) | 0.53 (0.43 to 0.63) | 0.70[b] (0.61 to 0.80) | 0.58 (0.46 to 0.71) | 0.54 (0.44 to 0.65) | 0.62[b] (0.52 to 0.72) |
| Fasting glycerol ($mmol \cdot L^{-1}$) | 0.04 (0.04 to 0.05) | 0.05 (0.04 to 0.06) | 0.06 (0.05 to 0.07) | 0.04 (0.03 to 0.06) | 0.04 (0.03 to 0.05) | 0.05 (0.04 to 0.07) |
| Fasting total cholesterol ($mmol \cdot L^{-1}$) | 4.6 (4.3 to 4.8) | 4.3 (4.0 to 4.5) | 5.0* (4.8 to 5.3) | 4.9 (4.5 to 5.2) | 4.2* (3.9 to 4.5) | 5.2 (4.9 to 5.5) |
| Fasting LDL-C ($mmol \cdot L^{-1}$) | 2.7 (2.5 to 2.9) | 2.3* (2.1 to 2.5) | 2.9 (2.7 to 3.2) | 2.9 (2.5 to 3.2) | 2.3* (2.1 to 2.6) | 3.0 (2.7 to 3.1) |
| Fasting HDL-C ($mmol \cdot L^{-1}$) | 1.3 (1.2 to 1.4) | 1.3 (1.2 to 1.4) | 1.3 (1.2 to 1.4) | 1.4 (1.2 to 1.5) | 1.3 (1.2 to 1.4) | 1.4 (1.3 to 1.5) |
| Fasting apoB ($mg \cdot dL^{-1}$) | 75 (67 to 83) | 71 (63 to 79) | 92[b] (84 to 100) | 80 (67 to 94) | 74 (63 to 85) | 89 (78 to 100) |
| Adipo-IR ($mmol \cdot L^{-1}$ x $pmol \cdot L^{-1}$) | 13.0 (9.1 to 16.9) | 15.3 (11.3 to 19.3) | 21.3[b] (17.5 to 25.2) | 18.6 (13.8 to 23.4) | 14.4 (10.5 to 18.4) | 15.6 (11.6 to 19.6) |
| WBC count ($n \cdot 10^9 \cdot L^{-1}$) | 5.2 (4.8 to 5.7) | 4.7 (4.3 to 5.1) | 4.5 (4.1 to 4.9) | 5.1 (4.5 to 5.7) | 5.1 (4.6 to 5.6) | 4.7 (4.2 to 5.1) |
| RBC count ($n \cdot 10^{12} \cdot L^{-1}$) | 4.42 (4.31 to 4.54) | 4.38 (4.26 to 4.49) | 4.49 (4.38 to 4.60) | 4.52 (4.40 to 4.64) | 4.38 (4.28 to 4.49) | 4.45 (4.35 to 4.55) |
| Hemoglobin ($g \cdot dL^{-1}$) | 13.2 (12.9 to 13.6) | 13.2 (12.9 to 13.6) | 13.6 (13.2 to 13.9) | 13.7 (13.3 to 14.1) | 13.1 (12.7 to 13.5) | 13.4 (13.1 to 13.8) |
| Hematocrit (%) | 38.7 (37.6 to 39.8) | 38.6 (37.5 to 39.7) | 39.0 (38.0 to 40.1) | 39.4 (38.3 to 40.5) | 38.0 (37.1 to 38.9) | 38.8 (37.9 to 39.7) |
| Platelet count ($n \cdot 10^9 \cdot L^{-1}$) | 227 (213 to 240) | 208 (195 to 222) | 210 (198 to 223) | 216 (195 to 237) | 216 (197 to 234) | 205 (187 to 222) |

Data shown are ANCOVA-adjusted mean (95% CI) for Week 4 and Week 12. [a]$p < 0.05$ LOWSUG vs MODSUG, [b]$p < 0.05$ LOWCHO vs MODSUG. βOHB = beta-hydroxybutyrate, FGF21 = fibroblast growth factor-21, TAG = triglyceride, NEFA = non-esterified fatty acid, WBC = white blood cell, RBC = red blood cell. Week 4 n=52, week 12 n=43.

**Table S7. Outcomes from continuous glucose monitoring during at baseline, week 1, and week 11-12 of moderate-sugar diet (MODSUG), low-sugar diet (LOWSUG), or low-carbohydrate diet (LOWCHO) in healthy men and women. Related to Figure 3.**

| | **Week 1** | | | **Week 12** | | |
|---|---|---|---|---|---|---|
| | MODSUG | LOWSUG | LOWCHO | MODSUG | LOWSUG | LOWCHO |
| Wear time (days) | 5 (1) | 5 (1) | 6 (1) | 10 (3) | 11 (2) | 11 (3) |
| Mean glucose ($mmol\cdot L^{-1}$) | 4.5 (4.4 to 4.7) | 4.7 (4.5 to 4.8) | 4.1[b] (4.0 to 4.2) | 4.8 (4.3 to 5.2) | 4.8 (4.4 to 5.3) | 4.5 (4.2 to 4.8) |
| Glucose CV (%) | 18.3 (16.0 to 20.5) | 15.3 (12.9 to 17.7) | 13.8[b] (11.7 to 15.9) | 18.4 (16.3 to 20.6) | 16.7 (14.4 to 19.0) | 15.8 (14.2 to 17.5) |
| Glucose management indicator (%) | 5.3 (5.2 to 5.3) | 5.3 (5.3 to 5.4) | 5.1[b] (5.0 to 5.1) | 5.4 (5.2 to 5.6) | 5.4 (5.2 to 5.6) | 5.2 (5.1 to 5.4) |
| Time spent above 13.9 $mmol\cdot L^{-1}$ (%) | 0 (0 to 0) | 0 (0 to 0) | 0 (0 to 0) | 0 (0 to 0) | 0 (0 to 0) | 0 (0 to 0) |
| Time spent between 13.9 and 10.1 $mmol\cdot L^{-1}$ (%) | 0 (0 to 0) | 0 (0 to 0) | 0 (0 to 0) | 1 (0 to 2) | 0 (0 to 0) | 0 (0 to 0) |
| Time spent between 10.0 and 3.9 $mmol\cdot L^{-1}$ (%) | 84 (71 to 97) | 86 (76 to 95) | 58 (45 to 70) | 88 (80 to 97) | 82 (67 to 97) | 74 (62 to 86) |
| Time spent between 3.8 and 3.0 $mmol\cdot L^{-1}$ (%) | 11 (5 to 17) | 14 (4 to 24) | 38 (27 to 49) | 10 (2 to 19) | 16 (3 to 30) | 21 (12 to 30) |
| Time spent below 3.0 $mmol\cdot L^{-1}$ (%) | 5 (0 to 14) | 0 (0 to 0) | 4 (2 to 6) | 1 (0 to 1) | 2 (0 to 3) | 5 (1 to 8) |

Wear time is mean (SD). Mean glucose, glucose CV, and glucose management indicator are ANCOVA-adjusted mean (95% CI) for Week 4 and Week 12 (with baseline values as the covariate). Time spent in range are unadjusted mean (95% CI) for all time-points. [a]$p < 0.05$ LOWSUG vs MODSUG, [b]$p < 0.05$ LOWCHO vs MODSUG. Week 1 n=39, Week 12 n=32.

**Table S9. Nutrition information of foods included in the alternative forced choice task used to measure relative food preference. Related to Figure 7.**

| Description | Energy density ($kcal \cdot g^{-1}$) | Fat (g per 100 g) | CHO (g per 100 g) | Sugars (g per 100 g) | Protein (g per 100 g) |
|---|---|---|---|---|---|
| ***High-carbohydrate sweet*** | | | | | |
| Banana | 1.03 | 0.5 | 23.2 | 20.9 | 1.2 |
| Strawberry trifle | 1.47 | 6.0 | 21.0 | 19.3 | 2.0 |
| Toffee and vanilla ice cream | 1.84 | 6.5 | 28.3 | 22.4 | 2.8 |
| Apple pie | 2.70 | 10.5 | 40.0 | 21.1 | 2.7 |
| Chocolate on toast | 3.46 | 12.5 | 49.8 | 21.5 | 7.4 |
| Chocolate digestive biscuits | 4.95 | 23.6 | 62.2 | 29.5 | 6.7 |
| ***High-carbohydrate savory*** | | | | | |
| Rice with sweetcorn | 1.17 | 1.2 | 22.6 | 1.2 | 4.1 |
| Tomato and basil pasta | 1.46 | 4.1 | 22.7 | 4.6 | 3.7 |
| Noodles with katsu sauce | 1.86 | 6.2 | 29.0 | 4.7 | 2.7 |
| French fries | 2.47 | 9.6 | 36.1 | 0.9 | 2.4 |
| Garlic bread | 3.39 | 13.0 | 44.8 | 2.7 | 9.7 |
| Cheese crackers | 5.00 | 23.0 | 63.5 | 8.8 | 7.7 |
| ***Low-carbohydrate savory*** | | | | | |
| Vegetarian chicken slices | 1.19 | 2.6 | 4.5 | 0.9 | 16.3 |
| Hard-boiled egg | 1.43 | 9.6 | 0.5 | 0.5 | 14.1 |
| Tuna and mayonnaise | 1.86 | 11.4 | 0.5 | 0.2 | 20.1 |
| Vegetarian bacon slices | 2.14 | 15.7 | 5.4 | 2.6 | 10.1 |
| Edam cheese | 3.12 | 24.0 | 0.5 | 0.5 | 24.0 |
| Peanuts and edamame beans | 4.70 | 37.4 | 7.2 | 4.8 | 21.7 |

**Table S10. RNA expression primer sequences. Related to Figure 6.**

| primers | | Sequence (5'-3') |
|---|---|---|
| FABP4 | Forward | ACTGGGCCAGGAATTTGACG |
| | Reverse | CTCGTGGAAGTGACGCCTT |
| IRS1 | Forward | AGTCTGTCGTCCAGTAGCACCA |
| | Reverse | ACTGGAGCCATACTCATCCGAG |
| IRS2 | Forward | CCACCATCGTGAAAGAGTGAAGA |
| | Reverse | CTGAAACAGTGCTGAGCGTC |
| TBC1D4 | Forward | ATGAGAGGTCGGCTTGGAAGTG |
| | Reverse | CGGAATCCTCTTCGGGAAACGT |
| GLUT4 | Forward | ACTGGACGAGCAACTTCATC |
| | Reverse | GAGGACCGCAAATAGAAGGAA |
| TBC1D1 | Forward | CACCCAGTGCCACTCGATTT |
| | Reverse | TGGCTTTATTACCCCGGGAC |
| Akt 1 | Forward | CGAGCTGTTCTTCCACCTGT |
| | Reverse | TAATGTGCCCGTCCTTGTCC |
| Akt 2 | Forward | CTGCGGAAGGAAGTCATCATT |
| | Reverse | GGTCGTGGGTCTGGAAGG |
| INSR | Forward | CGTCCCCAGAAAAACCTCTTC |
| | Reverse | ACGGCCACCGTCACATTC |
| PDK4 | Forward | TGGAGCATTTCTCGCGCTAC |
| | Reverse | ACAGGCAATTCTTGTCGCAAA |
| PPIA | Forward | CCCACCGTGTTCTTCGACATT |
| | Reverse | GGACCCGTATGCTTTAGGATGA |
| Leptin | Forward | TTTTGTCAAGTGTCATATGTAGGTGTC |
| | Reverse | CTCCCTTCTGCCCAAACATTC |
| Adiponectin | Forward | TGCCCAAAGAGGAGAGAGGAA |
| | Reverse | TCAGAAACAGGCACACAACTCA |
| SREBP1c | Forward | TTCTGACAGCCATGAAGACAG |
| | Reverse | CCGCATCTACGACCAGTG |
| HSL | Forward | TCAGTGTCTAGGTCAGACTGG |
| | Reverse | AGGCTTCTGTTGGGTATTGGA |
| FAS | Forward | CAGAGTCGGAGAACTTGCAG |
| | Reverse | GGAGGCATCAAACCTAGACAG |
| Actin | Forward | CCTCGCCTTTGCCGATCC |
| | Reverse | CATCACGCCCTGGTGCC |
| 18S | Forward | AACCCGTTGAACCCCATT |
| | Reverse | CCATCCAATCGGTAGTAGCG |
| LPL | Forward | GGACTGAGAGTGAAACCCATAC |
| | Reverse | GGAAGGAGTAGGTCTTATTTGTGG |
| PPARɣ | Forward | GATCCAGTGGTTGCAGAT |
| | Reverse | GAGGGAGTTGGAAGGCTCTTC |
| CPT1A | Forward | TCCAGTTGGCTTATCGTGGTG |
| | Reverse | CTAACGAGGGGTCGATCTTGG |
| GPR109A/HCAR2 | Forward | GCCGATCCAGAATGGCGG |
| | Reverse | TTCGTGCCACTGGAAGGTAT |
